# Prickly waterlily and rigid hornwort genomes shed light on early angiosperm evolution

**DOI:** 10.1038/s41477-020-0594-6

**Published:** 2020-02-24

**Authors:** Yongzhi Yang, Pengchuan Sun, Leke Lv, Donglei Wang, Dafu Ru, Ying Li, Tao Ma, Lei Zhang, Xingxing Shen, Fanbo Meng, Beibei Jiao, Lanxing Shan, Man Liu, Qingfeng Wang, Zhiji Qin, Zhenxiang Xi, Xiyin Wang, Charles C. Davis, Jianquan Liu

**Affiliations:** 1 Key Laboratory of Bio-Resource and Eco-Environment of Ministry of Education & State Key Laboratory of Hydraulics & Mountain River Engineering, College of Life Sciences, Sichuan University, Chengdu, China; 2 State Key Laboratory of Grassland Agro-Ecosystem, Institute of Innovation Ecology, Lanzhou University, Lanzhou, China; 3 School of Life Sciences, North China University of Science and Technology, Tangshan, China; 4 Institute of Insect Sciences, College of Agriculture and Biotechnology, Zhejiang University, Hangzhou, China; 5 Key Laboratory of Aquatic Botany and Watershed Ecology, Wuhan Botanical Garden, Chinese Academy of Sciences, Wuhan, China; 6 Department of Organismic and Evolutionary Biology, Harvard University Herbaria, Cambridge, MA USA

**Keywords:** Phylogenomics, Phylogenetics, Genome evolution

## Abstract

Angiosperms represent one of the most spectacular terrestrial radiations on the planet^1^, but their early diversification and phylogenetic relationships remain uncertain^2–5^. A key reason for this impasse is the paucity of complete genomes representing early-diverging angiosperms. Here, we present high-quality, chromosomal-level genome assemblies of two aquatic species—prickly waterlily (*Euryale ferox*; Nymphaeales) and the rigid hornwort (*Ceratophyllum demersum*; Ceratophyllales)—and expand the genomic representation for key sectors of the angiosperm tree of life. We identify multiple independent polyploidization events in each of the five major clades (that is, Nymphaeales, magnoliids, monocots, Ceratophyllales and eudicots). Furthermore, our phylogenomic analyses, which spanned multiple datasets and diverse methods, confirm that *Amborella* and Nymphaeales are successively sister to all other angiosperms. Furthermore, these genomes help to elucidate relationships among the major subclades within Mesangiospermae, which contain about 350,000 species. In particular, the species-poor lineage Ceratophyllales is supported as sister to eudicots, and monocots and magnoliids are placed as successively sister to Ceratophyllales and eudicots. Finally, our analyses indicate that incomplete lineage sorting may account for the incongruent phylogenetic placement of magnoliids between nuclear and plastid genomes.

## Abstract (web summary)

Genome assemblies of two aquatic species, prickly waterlily and rigid hornwort, clarify the early diversification and phylogeny of plants. Multiple independent polyploidization events are inferred in each of the five major angiosperm clades.

## Main

The angiosperms, or flowering plants, represent one of the most diverse and species-rich clades on Earth. They provide the vast majority of food consumed by humans and contribute substantially to global photosynthesis and carbon sequestration^1^. The origin of angiosperms was famously coined ‘an abominable mystery’ owing to their sudden appearance and rapid diversification^2–5^. To date, angiosperms include more than 350,000 species^6^ and occupy nearly every habitat from forests and grasslands to sea margins and deserts; angiosperms encompass a considerable variety of life forms, including trees, herbs, submerged aquatics and epiphytes. Resolving early angiosperm phylogeny is therefore critical for our understanding of such diversifying processes^1,7^.

Decades of efforts have greatly resolved the angiosperm phylogeny, illuminating their evolutionary history and helping to delineate major groups^2–5^. It has been identified that the three early-diverging angiosperm orders Amborellales, Nymphaeales and Austrobaileyales, which constitute remarkable morphological disparity and low species diversity, represent the earliest diverged angiosperm lineages^8^ (that is, the so-called ANA grade). However, the vast majority of angiosperms belong to the Mesangiospermae clade, which includes approximately 99% of all extant angiosperms. Eudicots and monocots are the two largest Mesangiospermae subclades, including around 75% and 22% of all species, respectively^9^; magnoliids represent a third subclade with about 9,000 species^10^; and the remaining two subclades, Chloranthales and Ceratophyllales, are morphologically unusual with only 77 and 7 species, respectively^10–12^. Despite the elucidation and the strong support for each of the five subclades of Mesangiosperms^4,13^, phylogenetic relationships among these clades remain uncertain, and different topologies have been proposed on the basis of various morphological^14^ and/or molecular lines of evidence^13,15–18^ (Supplementary Fig. 1).

Genomic data provide a rich and convincing means to resolve such evolutionary uncertainties. Despite the availability of numerous sequenced genomes from eudicots and monocots, early-diverging angiosperms remain poorly sampled, therefore inhibiting insights into these fundamental questions. To date, no nuclear genome has been sequenced for the four key orders—Austrobaileyales, Ceratophyllales, Chloranthales and Nymphaeales—which exhibit diverse life histories, extreme morphological variation and great evolutionary divergence. This lack of critical taxon sampling probably exacerbates phylogenetic uncertainty when inferring early angiosperm relationships. For example, nuclear genomes of three magnoliids (that is, *Cinnamomum kanehirae*, *Liriodendron chinense* and *Persea americana*) have been subsequently published^19–21^; however, phylogenetic analyses in these two studies resulted in conflicting placement of magnoliids relative to monocots and eudicots—that is, either monocots as the sister to a clade of magnoliids and eudicots, or magnoliids as the sister to monocots and eudicots^19–22^. Moreover, cases of deep phylogenetic incongruence between nuclear and organellar genomes have been recently reported in angiosperms^18–24^, but their causation (such as hybridization and incomplete lineage sorting (ILS)) has not been fully evaluated.

**Fig. 1:**
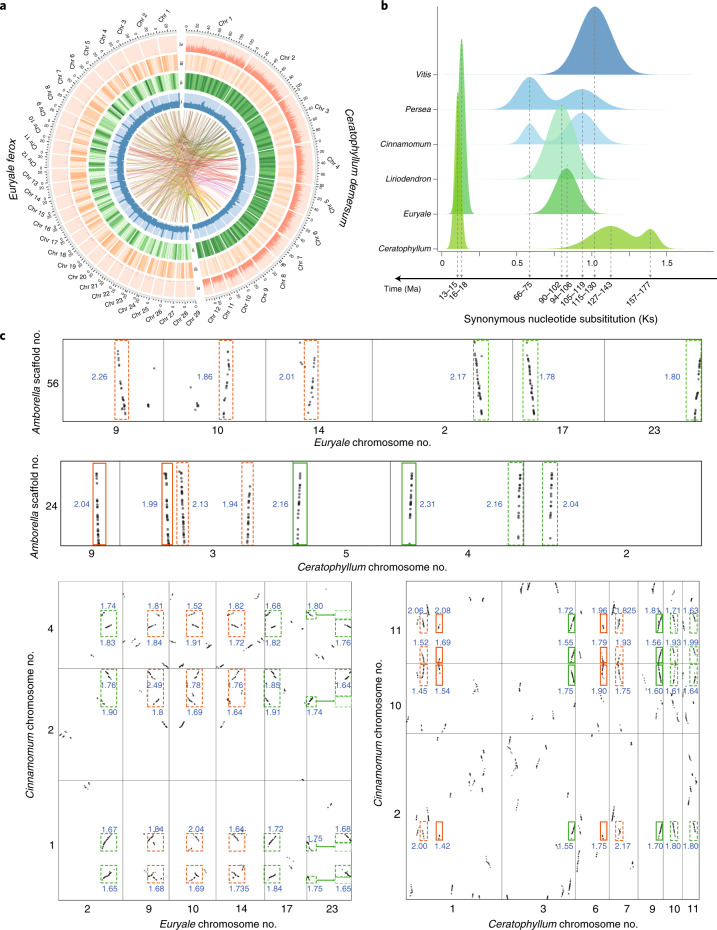
Comparative genomics analyses. **a**, The genomic features of *E. ferox* (pseudomolecules size: 721.2 Mb) and *C. demersum* (pseudomolecules size: 703.8 Mb). From inside to outside: GC content in 500-bp sliding windows (i; minimum–maximum, 0.2–0.8); repeat density in 10-kb sliding windows (ii; minimum–maximum: 0–1.0, coloured from white to dark green); gene density in 100-kb sliding windows (iii; minimum–maximum, 0–30, coloured from white to dark orange); and SNV density in 100-kb sliding windows (iv; minimum–maximum: 0–0.025). The links in the centre connect syntenic gene blocks that were detected using MCscan. Chr, chromosome. **b**, Distribution of average synonymous substitution levels (Ks) between syntenic blocks after evolutionary rate correction. **c**, Syntenic blocks (involving ≥10 colinear genes) between genomes. The corresponding median Ks value is shown for each block, and polyploidization events are represented by different colours.

Here we report the high-quality chromosomal-level genome assemblies of *E. ferox* Salisb. (prickly waterlily; estimated genome size of 768.2 Mb) and *C. demersum* L. (rigid hornwort; estimated genome size of 777.2 Mb), which are representatives of the two aquatic lineages Nymphaeales and Ceratophyllales, respectively (Supplementary Fig. 2, Supplementary Table 1). A total of 31.7 Gb of Oxford Nanopore Technologies (ONT) long reads and 47.4 Gb of Illumina short reads were generated for *Euryale*, and 80.5 Gb of ONT long reads and 46.4 Gb of short reads were generated for *Ceratophyllum* (Supplementary Fig. 3, Supplementary Table 2). ONT long reads were de novo assembled into contigs using the Canu assembler^25^, and two rounds of polishing were applied to the assembled contigs using Pilon^26^ with the Illumina short reads. The resulting genome assemblies of *Euryale* and *Ceratophyllum* were 725.2 Mb (N50 size of 4.75 Mb, where N50 corresponds to the minimum contig length needed to cover 50% of the genome) and 733.3 Mb (N50 size of 1.56 Mb), respectively (Supplementary Table 3). Moreover, a total of 84.4 Gb and 133.9 Gb of Hi-C data were generated using the Illumina platform for *Euryale* and *Ceratophyllum*, respectively. Assembled contigs were then clustered into 29 and 12 pseudochromosomes for *Euryale* and *Ceratophyllum*, respectively, using LACHESIS^27^ (Fig. 1a, Supplementary Tables 2 and 4). Both genome assemblies showed a high contiguity, completeness and accuracy (Fig. 1a, Supplementary Fig. 4, Supplementary Tables 5–7), and matched the chromosome counts obtained from cytological studies^28^. Using a combination of homology-based and transcriptome-based approaches, 40,297 and 30,138 protein-coding genes were predicted in the genomes of *Euryale* and *Ceratophyllum*, respectively (Supplementary Fig. 5, Supplementary Table 8). Moreover, 78.3% and 71.2% of all of the predicted protein-coding genes were clustered into gene families for *Euryale* and *Ceratophyllum*, respectively (Supplementary Note 1, Supplementary Fig. 6, Supplementary Table 9), and 85.6% and 89.8% of all of the predicted protein-coding genes were successfully annotated by at least one database (that is, SwissProt, TrEMBL, InterPro, GO or KEGG) for *Euryale* and *Ceratophyllum*, respectively (Supplementary Table 10). Furthermore, despite the similar genome size of these two species, the percentage of predicted repetitive elements was much higher in the genome of *Ceratophyllum* (that is, 38.35% versus 63.08% for *Euryale* and *Ceratophyllum*, respectively; Supplementary Table 11).

By constructing the distribution of synonymous substitutions per synonymous site (Ks) using syntenic paralogues within each genome, we detected two and three polyploidization events in the genomes of *Euryale* and *Ceratophyllum*, respectively (Supplementary Fig. 7, Supplementary Table 12). After correction for evolutionary rate^29^, the two polyploidization events in the genome of *Euryale* were estimated to occur at approximately 16–18 million and 94–106 million years ago (Ma), respectively; the three polyploidization events in *Ceratophyllum* were estimated to occur approximately 13–15 Ma, 127–143 Ma and 157–177 Ma, respectively (Fig. 1b). Furthermore, we identified polyploidization events in the genomes of *C. kanehirae*, *P. americana*, *L. chinense*, *Oryza sativa* and *Vitis vinifera*. Interestingly, the *Cinnamomum* and *Persea* genomes share two recent polyploidization events, and multiple independent polyploidization events have occurred in each of five major clades (that is, Nymphaeales, magnoliids, monocots, Ceratophyllales and eudicots; Fig. 1b, Supplementary Fig. 7), paralleling recent studies demonstrating that whole-genome duplication (WGD) is a widespread and potentially important evolutionary feature in angiosperms^30,31^. To better elucidate the polyploidy of our newly assembled genomes, we conducted a more focused comparative genomic analysis using *Amborella*, *Cinnamomum*, *Liriodendron* and *Vitis* as placeholders. Syntenic depth ratios of 6:1, 6:4, 6:2 and 6:3 were inferred in the *Euryale*–*Amborella*, *Euryale*–*Cinnamomum*, *Euryale*–*Liriodendron* and *Euryale*–*Vitis* comparisons, respectively, and 8:1, 8:4, 8:2 and 8:3 in the *Ceratophyllum*–*Amborella*, *Ceratophyllum*–*Cinnamomum, Ceratophyllum*–*Liriodendron* and *Ceratophyllum*–*Vitis* comparisons, respectively (Fig. 1c, Supplementary Figs. 8 and 9). On the basis of the syntenic relationships between and within each species, our analyses collectively demonstrate that *Euryale* underwent an ancient WGD followed by one whole-genome triplication, and *Ceratophyllum* has undergone three WGDs.

**Fig. 2:**
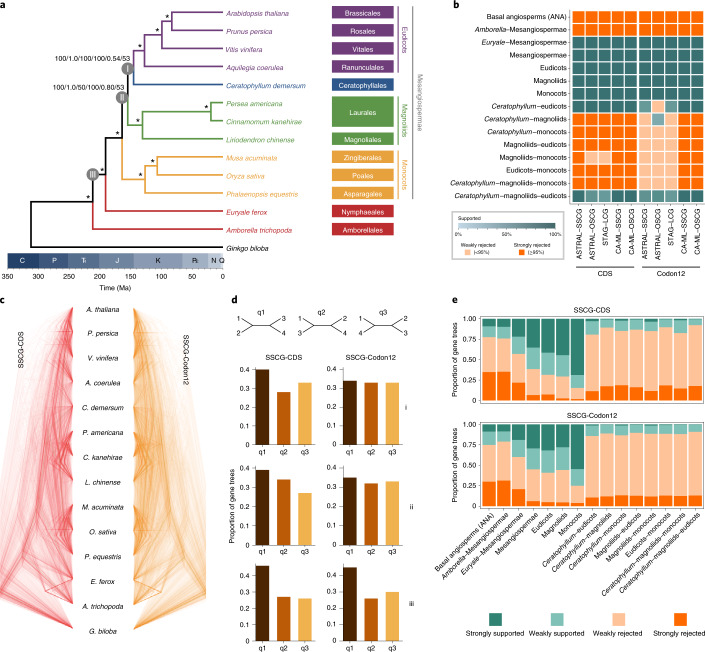
Phylogenomic analyses of early-diverging angiosperms. **a**, Chronogram of early-diverging angiosperms on the basis of the dataset SSCG-CDS inferred using MCMCTree. Bootstrap support percentages and posterior probabilities are indicated for each internal branch (from left to right, SSCG-CDS concatenation analysis using maximum likelihood (CA-ML), SSCG-CDS ASTRAL, LCG-CDS STAG, SSCG-Codon12 CA-ML, SSCG-Codon12 ASTRAL, LCG-Codon12 STAG), and an asterisk indicates 100 bootstrap support percentages and 1.0 posterior probabilities in all analyses. i, ii and iii indicate each internal branch. C, Carboniferous; J, Jurassic; K, Cretaceous; N, Neogene; P, Permian; , Triassic; Q, Quaternary; , Palaeogene. **b**, Species tree analysis using DiscoVista. Rows correspond to focal splits, and the spectrum indicates the support value for splits that are compatible with a species tree. Teal indicates themonophyly of a clade, and the different shades of teal indicate the level of its bootstrap support percentage (0 to 100%). Orange indicates rejection of a clade, and a 95% cut-off (instead of astandard 75%) was selected for strong rejection due to higher support values with genome-scale data. **c**, Superimposed ultrametric gene trees in a consensus DensiTree plot. The datasets SSCG-CDS and SSCG-Codon12 are shown in red and orange, respectively. **d**, The frequency of three topologies (q1–q3) around focal internal branches of ASTRAL species trees in the datasets SSCG-CDS and SSCG-Codon12. Each internal branch (labelled i, ii and iii) with four neighbouring branches can lead to three possible topologies (for example, q1, q2 and q3). **e**, Gene tree compatibility. The portion of gene trees for which focal splits are highly (or weakly) supported (or rejected). Weakly rejected splits are those that are not in the tree but are compatible if low support branches (below 75%) are contracted.

For the first time, the genomic taxon sampling represents two of the three orders in the ANA grade and four of the five subclades of Mesangiospermae. To resolve early angiosperm phylogeny, a total of 1,374 single-copy nuclear genes (SSCGs) were first identified with SonicParanoid^32^ using whole-genome sequences from 14 seed plants—that is, four eudicots (*Aquilegia coerulea*, *Arabidopsis thaliana*, *Prunus persica* and *Vitis*), three monocots (*Musa acuminate*, *Oryza* and *Phalaenopsis equestris*), three magnoliids (*Cinnamomum*, *Liriodendron* and *Persea*), *Ceratophyllum*, two ANA-grade species (*Amborella* and *Euryale*) and one gymnosperm (*Ginkgo biloba*; Supplementary Table 13). Aligned protein-coding regions were concatenated and analysed using two methods—(1) including all three codon positions (SSCG-CDS) and (2) including only the first and second codon positions (SSCG-Codon12). Moreover, for coalescent-based analyses, gene trees were individually estimated from each of the two datasets (SSCG-CDS and SSCG-Codon12), which were then input into ASTRAL^33^ for species tree inference (Supplementary Fig. 10). Our estimated gene trees are generally well supported (Supplementary Fig. 11), and both concatenation and coalescent analyses produced an identical strongly supported topology (Fig. 2a,b, Supplementary Figs. 12–14). Here, *Amborella* and *Euryale* were placed as successively sister to all other angiosperms, and monocots and magnoliids were inferred as successively sister to *Ceratophyllum* and eudicots. Our phylogenetic placement of magnoliids differs from APG, but is consistent with other studies that used various molecular markers, such as the plastid inverted repeat region^34^ and transcriptome data^18,35^. To avoid potential errors in orthology inference, we also extracted single-copy genes using OrthoMCL^36^ from the 14 seed plants described above as well as another gymnosperm (*Picea abies*). Only those genes sampled from at least 11 species were selected for downstream analyses, and a total of 2,302 single-copy genes (OSCG) were retained with an average of 1,859 genes for each species. Concatenation and coalescent analyses were similarly conducted as got those described above, and corroborated our phylogenetic findings (Fig. 2a, Supplementary Fig. 15). Furthermore, we took advantage of the newly developed species-tree inference method STAG^37^, which was designed to leverage gene trees estimated from multi-copy gene families. Only those genes sampled from all 15 seed plants were included, and a total of 2,356 low-copy genes (LCG) were retained. Species trees inferred from datasets including all three codon positions (LCG-CDS) and the first and second codon positions only (LCG-Codon12) were topologically identical to the ones described above (Fig. 2a, Supplementary Fig. 16), suggesting that our findings are robust.

Despite the fact that the same set of phylogenetic relationships was consistently recovered when nuclear genes were analysed simultaneously, topological conflicts among gene trees were widely observed as visualized using DensiTree^38^ (Fig. 2c). A major discordance was identified in the datasets SSCG-CDS and SSCG-Codon12 involving the relationship between *Amborella* and Nymphaeales (Fig. 2c). For the datasets SSCG-CDS and SSCG-Codon12, 46.3% and 44.5% of all 1,374 gene trees supported *Amborella* and *Euryale* as successively sister to all other angiosperms, respectively; 27.2% and 26.0% supported *Amborella* as sister to *Euryale*, respectively; and the other 26.5% and 29.5% supported *Euryale* alone as the first lineage of angiosperms, respectively (Fig. 2d, Supplementary Figs. 17–19). We also summarized gene tree discordance using DiscoVista^39^, and similar results were observed for the datasets SSCG-CDS and SSCG-Codon12—that is, a substantial fraction of gene trees were incongruent with species trees regarding the placement of *Amborella* and Nymphaeales (Fig. 2e, Supplementary Figs. 17–19). Moreover, conflicting phylogenetic placements of Ceratophyllales were observed in the gene trees. For the datasets SSCG-CDS and SSCG-Codon12, 39.7% and 34.2% of 1,374 gene trees supported *Ceratophyllum* as sister to eudicots, respectively; 27.6% and 32.9% supported *Ceratophyllum* as sister to monocots, respectively; and the other 32.7% and 32.9% supported *Ceratophyllum* as sister to magnoliids, respectively (Fig. 2d, Supplementary Figs. 17–19). These analyses indicate that there is probably substantial ILS during early angiosperm evolution and greatly highlight the phylogenomic complexity of resolving early-diverging angiosperms.

Furthermore, phylogenetic analyses of these 15 seed plants inferred from 72 concatenated plastid genes strongly support magnoliids as the first diverging lineage of Mesangiospermae (Supplementary Fig. 20, Supplementary Table 14). This placement of magnoliids is incongruent with our nuclear phylogeny, but consistent with a recent study that analysed 2,881 plastomes^4^. Thus, what might account for this deep phylogenetic incongruence between nuclear and plastid genomes? As multiple independent polyploidization events were identified in magnoliids, monocots, Ceratophyllales and eudicots (Fig. 1), allopolyploidization or hybridization is one probable source of genomic discordance. We first assessed putative hybridization events in our phylogeny using PhyloNetworks. Although three cases of hybridization were inferred, none involved the three species of magnoliids (that is, *Cinnamomum*, *Liriodendron* and *Persea*; Supplementary Fig. 21). Furthermore, very short internal branches among four subclades of Mesangiospermae were observed in all our analyses, corresponding to an estimated divergence time of around 20 Ma (Supplementary Figs. 12–16, 20 and 22). We therefore tested whether ILS might better explain this discordance. We simulated 20,000 gene trees under the multispecies coalescent model^40^ on the basis of the ASTRAL tree inferred from the dataset SSCG-CDS. We found considerable agreement between simulated and empirical gene trees (overall correlation coefficient, Spearman’s *ρ* = 0.97, *P* < 0.01; Supplementary Fig. 23), suggesting that the multispecies coalescent model is a good fit to our data. Here the relative frequencies of various topologies, including the topology inferred from plastomes, were consistent with frequencies of ILS as estimated from our coalescent analyses (Supplementary Fig. 23c,d). These results indicate that ILS may well account for the incongruent placement of magnoliids between nuclear and plastid genomes. Finally, as sparse taxon sampling could result in these discordant results^41^, we increased our taxon sampling in the nuclear phylogeny by adding taxa with published genomes. A total of 612 ‘mostly’ single-copy orthologous genes (SCOG) were extracted from 213 nuclear genomes, which included 211 angiosperms representing 33 orders and 67 families as well as two gymnosperms as outgroups (Supplementary Table 15), and the average number of genes per taxon was 545. Coalescent analyses of the datasets SCOG-CDS and SCOG-Codon12 recovered the same relationships among the four subclades of Mesangiospermae (Supplementary Figs. 24–26), suggesting that our results are robust to additional taxa sampling.

In summary, the high-quality genomes of prickly waterlily and rigid hornwort greatly help to clarify phylogenetic relationships of early-diverging angiosperms. Moreover, these genomic resources are essential for future comparative investigations of genic evolution that underpin the morphological, physiological and ecological diversification of early angiosperms (Supplementary Notes 1–3, Supplementary Figs. 27–28, Supplementary Tables 16–22).

## Methods

### Plant materials and DNA sequencing

Fresh leaves of *E. ferox* and whole plants of *C. demersum* were obtained for DNA extraction and sequencing. Total genomic DNA was extracted using the CTAB method^42^. The library for ONT sequencing was constructed using large (>20 kb) DNA fragments with the Ligation Sequencing Kit 1D (SQK-LSK108), and sequenced using the GridION X5 platform. Adapters and low-quality nucleotides (that is, mean quality score <7) were trimmed. Paired-end libraries with an insertion size of 350 bp were constructed according to the manufacturer’s protocols and sequenced using the Illumina HiSeq 2500 System. Illumina reads were filtered using following criteria: (1) containing more than 5% unidentified nucleotides, (2) more than 65% of bases with a Phred quality score <7 and (3) more than 10 bp adapter sequences (allowing 2 bp mismatches). For the high-throughput chromosome conformation capture (Hi-C) analysis, fresh leaves were fixed in formaldehyde solution (1%), and chromatin was cross-linked and digested using the restriction enzyme HindIII. The 5′ overhangs were filled-in with biotinylated nucleotides, and free blunt ends were ligated. After ligation, crosslinks were reversed and the DNA was purified to remove protein. Purified DNA was treated to remove biotin that was not internal to ligated fragments. DNA was sheared into fragments of ~350 bp, and sequenced using the Illumina platform.

### Genome size estimation

Genome size was estimated using the *k*-mer analysis of Illumina 150-bp paired-end reads. The *k*-mer depth-frequency distribution was generated using SOAPec^43^ (v.2.0.1, https://sourceforge.net/projects/soapdenovo2/) with the following parameters: -k 17 -q 33 -t 10. The genome size was then calculated according to the following formula^44^: genome size = *k*-mer coverage/mean *k*-mer depth (Supplementary Table 1).

### Genome assembly

ONT long reads were de novo assembled using the Canu assembler^25^ (v.1.7, https://github.com/marbl/canu/), and two rounds of polishing were applied to the assembled contigs using Pilon^26^ (v.1.22, https://github.com/broadinstitute/pilon/) with the Illumina short reads. HiC-Pro^45^ v.2.10.0 was used to evaluate the quality of Hi-C data. Valid interaction pairs were mapped to the contigs and anchored to the pseudochromosomes using LACHESIS^27^ (https://github.com/shendurelab/LACHESIS).

### Transcriptome sequencing and assembly

For each species, total RNA was extracted from various plant organs (roots, leaves and stems), and residual DNA was removed using the DNA-free DNA Removal Kit. A total of 18.69 Gb and 5.85 Gb of reads were generated using the Illumina platform for *E. ferox* and *C. demersum*, respectively. Transcripts were assembled from filtered reads using Trinity^46^ v.2.8.4 with additional parameters including ‘--trimmomatic --normalize_reads’.

### Annotation of repetitive elements

To annotate repetitive elements, we utilized a combination of evidence-based and de novo approaches. Genome assemblies were first searched using RepeatMasker^47^ (v.4.0.7, http://repeatmasker.org/) against the Repbase database (http://www.girinst.org/repbase). Next, a de novo repetitive-element library was constructed using RepeatModeler (v.1.0.11, http://repeatmasker.org/RepeatModeler.html). This de novo repetitive-element library was then utilized by RepeatMasker to annotate repetitive elements. Results from these two runs of RepeatMasker were merged.

### Protein-coding gene prediction and functional annotation

The identification of protein-coding genes was based on transcriptome data and ab initio prediction. RNA transcripts were first mapped to the assembled genome using PASA^48^ (Program to Assemble Spliced Alignment v.2.3.3). Valid transcript alignments were clustered on the basis of mapping location and assembled into gene structures, and then the high-quality gene models were selected for training by AUGUSTUS^49^ v.3.2.3. Moreover, intron hints were generated using the script bam2hints provided by AUGUSTUS. Next, AUGUSTUS was utilized for ab initio gene prediction on the hard-masked genome assembly, and all of the predictions were integrated using EvidenceModeler^49^ (EVM, v.1.1.1) to generate consensus gene sets. For functional annotation, our predicted protein-coding genes were searched against the Swiss-Prot and TrEMBL databases, as well as the InterPro database using InterProScan^50^ release 5.33–72.0.

### Polyploidization analysis

Seven genomes were selected for our polyploidization analysis, that is, *A. trichopoda* (Amborellales; At), *C. demersum* (Ceratophyllales), *C. kanehirae* (magnoliids), *E. ferox* (Nymphaeales; Ef), *L. chinense* (magnoliids), *O. sativa* (monocots), *P. Americana* (magnoliids) and *V*. *vinifera* (eudicots; Vv). Colinear genes within each genome and between genomes were inferred using MCScan^51^ v.0.8 according to the combined information of gene similarity and gene order. Synonymous substitutions per synonymous site (Ks) between colinear genes were estimated using the Nei–Gojobori approach^52^ as implemented in the PAML^53^ package v.4.9 h. The median Ks values were selected to represent each syntenic block, and the probability density distribution curve of Ks was estimated using MATLAB with the kernel smoothing density function (ksdensity; bandwidth was typically set to 0.025). Multipeak fitting of the curve was performed using the Gaussian approximation function (cftool) in MATLAB, and the coefficient of determination (*R*^2^) was set as at least 0.95.

Furthermore, we performed a correction to the Ks values to distinguish the order of each polyploidization event using a similar method to a method used previously^54^. Here, supposing that the Ks values of colinear orthologues between two genomes *i* and *j* are $X_{i - j}:N\left( {\mu _{i - j},\sigma _{i - j}^2} \right)$, where *N* represents the normal distribution, *μ* represents the mean value and *σ* represents the standard deviation. We further supposing that the ratio of the evolutionary rate of species *i* to the assumed averaged evolutionary rate of angiosperms is *r*_*i*_, the correction coefficient *λ*_*i*_ is defined as $\lambda _i = \frac{1}{{r_i}}$ and, accordingly, the correction coefficient factor of *X*_*i* *−* *j*_ is defined as $\lambda _{ij} = \lambda _i \lambda _j$

The mean of the corrected *X*_*i* *−* *j-correction*_ can be inferred to be

$${\upmu}_{{\mathrm{i}} - {\mathrm{j}} - {\mathrm{correction}}} = \mu _{i - j}\lambda _i \lambda _j$$

For *E*[*tX*] = *tE*[*X*] and *D*[*tX*] = *t*^2^*D*[*X*]

we can get

$$X_{i - j - {\mathrm{correction}}}:N\left( {\mu _{i - j - {\mathrm{correction}}},\sigma _{i - j - correction}^2} \right) = N\left( {\lambda _i\lambda _j\mu _{i - j},\lambda _i^2\lambda _j^2\sigma _{i - j}^2} \right)$$

As *Amborella* and *Euryale* are basal angiosperms, the divergence between *Amborella* or *Euryale* and other plants occurred at the same time. Therefore, for genome *i*

$$\frac{{\mu _{{\mathrm{At}} - i - {\mathrm{correction}}}}}{{\mu _{{\mathrm{At}} - {\mathrm{Vv}} - {\mathrm{correction}}}}} = \frac{{\mu _{{\mathrm{At}} - i} \lambda _{{\mathrm{At}}}\lambda _i}}{{\mu _{{\mathrm{At}} - {\mathrm{Vv}}}\lambda _{{\mathrm{At}}}\lambda _{{\mathrm{Vv}}}}} = \frac{{\mu _{{\mathrm{At}} - i} \lambda _i}}{{\mu _{{\mathrm{At}} - {\mathrm{Vv}}} \lambda _{{\mathrm{Vv}}}}} = 1$$

$$\frac{{\mu _{{\mathrm{Ef}} - i - {\mathrm{correction}}}}}{{\mu _{{\mathrm{Ef}} - {\mathrm{Vv}} - {\mathrm{correction}}}}} = \frac{{\mu _{{\mathrm{Ef}} - i} \lambda _{{\mathrm{Ef}}} \lambda _i}}{{\mu _{{\mathrm{Ef}} - {\mathrm{Vv}}} \lambda _{{\mathrm{Ef}}} \lambda _{{\mathrm{Vv}}}}} = \frac{{\mu _{{\mathrm{Ef}} - i} \lambda _i}}{{\mu _{{\mathrm{Ef}} - {\mathrm{Vv}}} \lambda _{{\mathrm{Vv}}}}} = 1$$

$$\frac{{\lambda _i}}{{\lambda _{{\mathrm{Vv}}}}} = a_i = {\mathrm{mean}}\left\{ {\frac{{\mu _{{\mathrm{At}} - {\mathrm{Vv}}}}}{{\mu _{{\mathrm{At}} - i}}},\frac{{\mu _{{\mathrm{Ef}} - {\mathrm{Vv}}}}}{{\mu _{{\mathrm{Ef}} - i}}}} \right\}$$

$$\lambda _i = \lambda _{{\mathrm{Vv}}} a_i$$

The *a*_*i*_ represents the mean ratio value among the observed Ks peak between *Amboralla* and *Vitis*, or *Euryale* and *Vitis*. After the divergence from the other studied plants, *A*. *trichopoda* has not been affected by polyploidization anymore; thus, we assumed that the evolutionary rate of *Amborella* genes is relatively stable and, therefore, set *λ*_At_ = 1. The plant *i* with the slowest evolutionary rate is the most likely to have the same evolutionary rate as *Amborella*, that is, $\max \left\{ {\lambda _i} \right\} = 1$ and $\lambda _{{\mathrm{Vv}}} = \frac{{\max \left\{ {\lambda _i} \right\}}}{{\max \left\{ {a_i} \right\}}} = \frac{1}{{\max \left\{ {a_i} \right\}}}$. We determined the approximate value for *V*. *vinifera* (*λ*_Vv_) using the above estimator, and used it to assess the correction coefficient ratio for each species. The major-eudicot common hexaploidy 115–130 Ma (refs. ^55,56^), inferred by grape duplicated genes, was used as the reference to date the ages for the other polyploidization and speciation events (Supplementary Table 12).

### Phylogenetic analyses

To infer the phylogenetic placements of *E. ferox* and *C. demersum*, SSCGs were first identified using SonicParanoid^32^ v.1.0 from 14 seed plants (SSCG; Supplementary Table 13). For each gene, amino acid sequences were aligned using MAFFT^57^ v.7.402, and then DNA sequences were aligned according to the corresponding amino acid alignments using PAL2NAL^58^ v.14. For datasets SSCG-CDS and SSCG-Codon12, the maximum likelihood (ML) trees were inferred from concatenated gene sequences using IQ-TREE^59^ v.1.6.9, which automatically selected the best-fit substitution model using ModelFinder^60^. Bootstrap support was estimated using 1,000 replicates of the ultrafast bootstrap approximation^61^ (-bb 1000 -m MFP). For coalescent-based analyses, gene trees were first estimated using IQ-TREE; the gene trees were then utilized by ASTRAL v.5.6.1 to infer species trees with quartet scores and posterior probabilities. Furthermore, SSCGs were identified using OrthoMCL^36^ v.2.0.9 (OSCG) with one more Gymnosperm (*P. abies*; Supplementary Table 13). Species trees were inferred from the datasets OSCG-CDS and OSCG-Codon12 using concatenation and coalescent methods as described above. Finally, we extracted low-copy genes from 15 seed plants (LCG). Here, each gene was required to include at least 1 sequence from each of the 15 species and less than 5 homologous sequences per species. For the datasets LCG-CDS and LCG-Codon12, gene trees were first estimated using IQ-TREE^59^; these gene trees were then utilized to construct species trees using STAG^37^ v.1.0.0.

For plastid genes, the 72 CDS of protein-coding genes were extracted from 15 seed plants (Supplementary Table 14), and aligned using MAFFT and PAL2NAL as described above. The ML trees were inferred from concatenated gene sequences using RAxML^62^ v.7.2.8 with 100 bootstraps.

For expanded taxon sampling, sequence similarity was first assessed for all of the amino acid sequences from 213 species (211 angiosperms and 2 gymnosperms; Supplementary Table 15) using MMseqs2^63^ with an *E-*value threshold of 1 × 10^−5^, and then grouped using a Markov cluster algorithm^64^. Here, each gene was required to include sequences from more than 180 species. Next, ‘mostly’ single-copy orthologous genes (SCOG) were identified using a tree‐based method^65,66^. Each gene was aligned using MAFFT and PAL2NAL as described above, and species trees were inferred from datasets SCOG-CDS and SCOG-Codon12 using ASTRAL.

### Visualizations of gene-tree discordance

Gene trees were first converted to ultrametric trees using the R package Phybase^67^, and then superimposed using DensiTree^38^ (Fig. 2c). Quartet frequencies of the internal branches in the species tree were calculated using ASTRAL^33^ with the parameter ‘-t=2’ (Supplementary Figs. 17 and 19). Furthermore, the analysis of gene-tree compatibility was conducted using DiscoVista^39^ v.1.0. Here, a total of 15 species groups were considered, and 7 of which are identified in our species tree, including: (1) 12 Mesagiospermae, (2) 4 eudicots, (3) 3 magnollids, (4) 3 monocots, (5) *Euryale* and all Mesangiospermaes, (6) Ceratophyllaceae and eudicots, and (7) Magnoliids and (Ceratophyllaceae and eudicots). The other 8 species groups were: (1) basal angiosperms (that is, *Ambrorella* and *Euryale*), (2) *Ambrorella* and Mesangiospermae, (3) Ceratophyllaceae and magnoliids, (4) Ceratophyllaceae and monocots, (5) magnoliids and eudicots, (6) magnoliids and monocots, (7) eudicots and monocots, and (8) Ceratophyllaceae, magnoliids and monocots (Fig. 2e, Supplementary Fig. 18). Bootstrap support values of at least 75% were interpreted as highly supported^68^ (Fig. 2e).

### Divergence time estimation

Divergence time was estimated for the dataset SSCG-CDS using the program MCMCTree in the PAML^53^ package v.4.9 h. After a burn-in of 5,000,000 iterations, the MCMC process was performed 20,000 times with sample frequency of 5,000. Convergence was assessed using two independent runs. We used the following age constraints in our estimation procedure: the divergence between angiosperms and gymnosperms (330–289 Ma; http://www.timetree.org/), the crown group of angiosperms (267–132.9 Ma)^4^, the crown group of monocots (184–113 Ma)^4^ and the crown group of eudicots (161–125 Ma)^4^.

### Hybridization inference and ILS simulation

Hybridization was detected for the dataset OSCG-CDS using the maximum pseudolikelihood estimation of phylogenetic networks, as implemented in PhyloNetworks^69^ v.0.9.0. The maximum allowed number of hybridizations was set from hmax=0 to hmax=10, each with 100 runs. The ILS simulation was performed as previously described^70^. We simulated 200,000 gene trees under the multispecies coalescent model using the R function sim.coaltree.sp as implemented in the package Phybase^67^ v.1.5. The internal branch lengths of the ASTRAL tree were used for the simulation, and all terminal branches were set to 1 (as 1 allele was generated for each species). It should be noted that internal branch lengths (in coalescent units) in our simulation might have been overestimated, as the cause of gene tree heterogeneity was assumed to result from only ILS. Gene-tree quartet frequencies were calculated for simulated and empirical datasets, and the correlation test was performed using the cor.test function in R.

### Demographic inference

The pairwise sequentially Markovian coalescent (PSMC) model^71^ v.0.6.4-r49 was used to infer the demographic history of seven species, that is, *A*. *trichopoda* (Amborellales), *E. ferox* (Nymphaeales), *C. demersum* (Ceratophyllales), *C. kanehirae* (magnoliids), *L*. *chinense* (magnoliids), *P*. *equestris* (monocots) and *V*. *vinifera* (eudicots). The genome of *E*. *ferox* showed very low heterozygosity (about 0.02%; Supplementary Table 22) and, therefore, two individuals were included in the PSMC analysis^72^. For each species, whole-genome resequencing data (at least 30-fold coverage) were obtained from NCBI (Supplementary Table 22). Reads were mapped to the assembled genome, and the consensus sequences were extracted. The analysis was performed using the following parameters: -N25 -t15 -r5 -p ‘4+25×2+4+6’. Here, for *A. trichopoda*, *C kanehirae*, *L. chinense* and *V*. *vinifera*, the generation time and mutation rate were obtained from previous studies^7,19,20,73^. For other three species (that is, *E. ferox*, *C. demersum* and *P*. *equestris*), the mutation rate was first estimated using r8s^74^. Furthermore, as *E. ferox* is an annual species, the generation time was set to 1. For perennial species, as the generation time is difficult to determine precisely^75^, we tested the generation time for both 3 and 5 years, and similar results were obtained.

### Reporting Summary

Further information on research design is available in the Nature Research Reporting Summary linked to this article.

## Supplementary information

Supplementary Information Supplementary notes, figures, tables and references.

Reporting Summary

## Data Availability

All of the raw sequence reads used in this study have been deposited at NCBI under the BioProject accession numbers PRJNA552436 (*E. ferox*) and PRJNA552433 (*C. demersum*). The assemblies and annotations are available from the CoGe comparative genomics platform at https://genomevolution.org/CoGe/GenomeInfo.pl?gid=56574 (*E. ferox* chromosome assembly), https://genomevolution.org/CoGe/GenomeInfo.pl?gid=56571 (*E. ferox* contig assembly), https://genomevolution.org/CoGe/GenomeInfo.pl?gid=56572 (*C. demersum* chromosome assembly) and https://genomevolution.org/CoGe/GenomeInfo.pl?gid=56569 (*C. demersum* contig assembly).

## Code availability

The custom scripts have deposited in GitHub (https://github.com/yongzhiyang2012/Euryale_ferox_and_Ceratophyllum_demersum_genome_analysis).
