# Supplementary material for: Prickly waterlily and rigid hornwort genomes shed light on early angiosperm evolution
Source: Nat Plants. 2020 Feb 24;6(3):215–22. doi: 10.1038/s41477-020-0594-6 (PMC8075997; doi:10.1038/s41477-020-0594-6)
Supplement: Supplementary file 1 — Supplementary notes, figures, tables and references. [file 41477_2020_594_MOESM1_ESM.pdf]

nature plants

SUPPLEMENTARY INFORMATION

https://doi.org/10.1038/s41477-020-0594-6

In the format provided by the authors and unedited.

Prickly waterlily and rigid hornwort genomes shed light on early angiosperm evolution

Yongzhi Yang[1,2,7], Pengchuan Sun[3,7], Leke Lv[1], Donglei Wang[1], Dafu Ru[1], Ying Li[2], Tao Ma[1], Lei Zhang[1], Xingxing Shen[4], Fanbo Meng[3], Beibei Jiao[3], Lanxing Shan[3], Man Liu[3], Qingfeng Wang[5], Zhiji Qin[3], Zhenxiang Xi[1]✉, Xiyin Wang[3]✉, Charles C. Davis[6]✉ and Jianquan Liu[1,2]✉

[1]Key Laboratory of Bio-Resource and Eco-Environment of Ministry of Education & State Key Laboratory of Hydraulics & Mountain River Engineering, College of Life Sciences, Sichuan University, Chengdu, China. [2]State Key Laboratory of Grassland Agro-Ecosystem, Institute of Innovation Ecology, Lanzhou University, Lanzhou, China. [3]School of Life Sciences, North China University of Science and Technology, Tangshan, China. [4]Institute of Insect Sciences, College of Agriculture and Biotechnology, Zhejiang University, Hangzhou, China. [5]Key Laboratory of Aquatic Botany and Watershed Ecology, Wuhan Botanical Garden, Chinese Academy of Sciences, Wuhan, China. [6]Department of Organismic and Evolutionary Biology, Harvard University Herbaria, Cambridge, MA, USA. [7]These authors contributed equally: Yongzhi Yang, Pengchuan Sun. ✉e-mail: zxi@scu.edu.cn; wangxiyin@vip.sina.com; cdavis@oeb.harvard.edu; liujq@nwipb.ac.cn

Supplementary Information

Prickly waterlily and rigid hornwort genomes shed light on early angiosperm evolution

Yang and Ma et al.

Supplementary Notes **2**
Supplementary Figures **6**
Supplementary Tables **36**
Supplementary References **75**

Supplementary Notes

Supplementary Note 1

For the gene family evolution analysis, a total of 316,967 genes from 14 species (12 angiosperms and 2 gymnosperms) were clustered into 28,710 gene families with an average of eleven genes per family (Supplementary Table 9). In addition, we found that 78.3% and 71.2% of all predicted protein-coding genes were clustered into gene families for *Euryale* and *Ceratophyllum*, respectively. A total of 1,139 unique gene families in *Euryale* were enriched in 76 GO categories, and their main functions were involved in response to stimulus, cellular metabolism, and macromolecule metabolism (Supplementary Table 16). A total of 1,016 unique gene families in *Ceratophyllum* were enriched in 78 GO categories, and their main functions were involved in defense response and cell wall modification (Supplementary Table 17). Using CAFÉ[1], we identified 6,501 and 3,473 expanded gene families, and 5,927 and 5,024 contraction gene families for *Euryale* and *Ceratophyllum*, respectively. We found that the expanded gene families in *Euryale* were mainly associated with defense responding, especially for the light and cell wall modification (Supplementary Table 18). For *Ceratophyllum*, however, the expanded gene families were mainly involved in transcription and primary metabolic process (Supplementary Table 19). The contracted gene families within these two species were mainly involved in the membrane component, hydrolase activity, and extracellular matrix related processes (Supplementary Tables 20 and 21). The unique and expanded gene families related to stimulus and regulation of transcription may have helped these two species adapt the new specialized aquatic environment when recolonizing the aquatic habitat after their ancestors had dwelled in the land for a long time. Especially, some cell-wall related gene families also experienced the rapid contractions (Supplementary Tables 20 and

21), which may explain the vascular cambium changes in these two aquatic lineages. Further studies are needed to confirm whether genomic changes may account for phenotypic and habitat changes of these two aquatic lineages.

Supplementary Note 2

For each gene tree, the bootstrap support for the internal branch in the data set SSCG-CDS were significantly greater than those in the data set SSCG-Codon12 (T-test, P<0.01) (Supplementary Fig. 11a,b), suggesting a higher resolution for the SSCG-CDS data set. In addition, we found a positive correlation between the branch length and bootstrap support (Spearman $P < 0.01$) (Supplementary Fig. 11c). Furthermore, we conducted a test of the null hypothesis that a short internal branch in the estimated species tree should be replaced by a polytomy[2]. Here, three internal branches were selected, i.e., branch A involving *Amborella* and *Euryale*, branch B involving monocots, magnoliids, Ceratophyllales, and eudicots, and branch C involving Magnoliids, Ceratophyllales, and eudicots (Supplementary Fig. 14a). We performed the test[2] using ASTRAL ('-t 10'), and randomly selected 50, 100, 150, …, and 1,300 gene trees. Each random sampling was repeated 20 times. We found that the null hypothesis was rejected for all three short internal branches in our species tree as the number of sampled genes in the SSCG-CDS data set increased to 400 (Supplementary Fig. 14). Finally, our SSCG and OSCG data sets consist of 1,374 and 2,302 single-copy genes, respectively. In contrast, fewer single-copy genes were phylogenetically analyzed in these recently published genome papers (i.e., 211, 235, 176, and 82 genes in the genome paper of *Cinnamomum*[3], *Liriodendron*[4], *Persea*[5], and *Piper*[6], respectively).

Supplementary Note 3

The major incongruence between nuclear and plastid phylogenies involves the placement of magnoliids. In the plastome phylogeny, magnoliids were placed as the basal lineage in Mesangiospermae (Supplementary Fig. 20). For nuclear genes, however, magnoliids were placed as sister to Ceratophyllales + eudicots (Supplementary Figs. 12–13, 15–16, 24–26). We first used PhyloNetworks[7] to detect all possible hybridization events in Mesangiospermae by allowing the number of hybridization events from one to ten. Only three potential hybridization events were detected: one between the core eudicots and monocots, one within eudicots, and one within monocots (Supplementary Fig. 21).

We used a simulation method[8] to explore whether such incongruence can be explained by incomplete lineage sorting (ILS). A total of 20,000 gene trees were simulated under the multispecies coalescent model based on our ASTRAL species tree. Gene tree quartet frequencies were calculated for simulated and empirical data sets, and we found a significant correlation between simulated and empirical gene trees ($r^2 = 0.97$, $P < 0.01$; Spearman's rho) (Supplementary Fig. 23a,b). We also found that the topology of the plastome phylogeny showed a similar frequency between simulated and empirical data sets (Supplementary Fig. 23c,d). These results support that ILS may well account for the incongruence identified here.

Using PSMC analyses, we additionally tested whether early angiosperms shared similar demographic histories in response to the recent climatic oscillations. All species examined here showed decreased effective population sizes during the Quaternary glaciation, and retained small effective population sizes without the occurrence of post-glacial expansion (Supplementary Fig. 28 and Supplementary Table 22). It remains unclear whether this is common for all early-diverging

angiosperms.

Furthermore, we found that multiple polyploidizations occurred within each of these early-diverging lineages (Supplementary Fig. 28). Polyploidy is often linked with key innovations and subsequent species diversification in major clades of angiosperms[9]. In addition, polyploid plants may have a higher adaptability and increased tolerance to different environmental conditions[10,11]. Thus, a broader phylogenetic sampling is required to better understand the evolutionary significance of polyploidy in species-poor lineages of early angiosperms[12].

Supplementary Figures

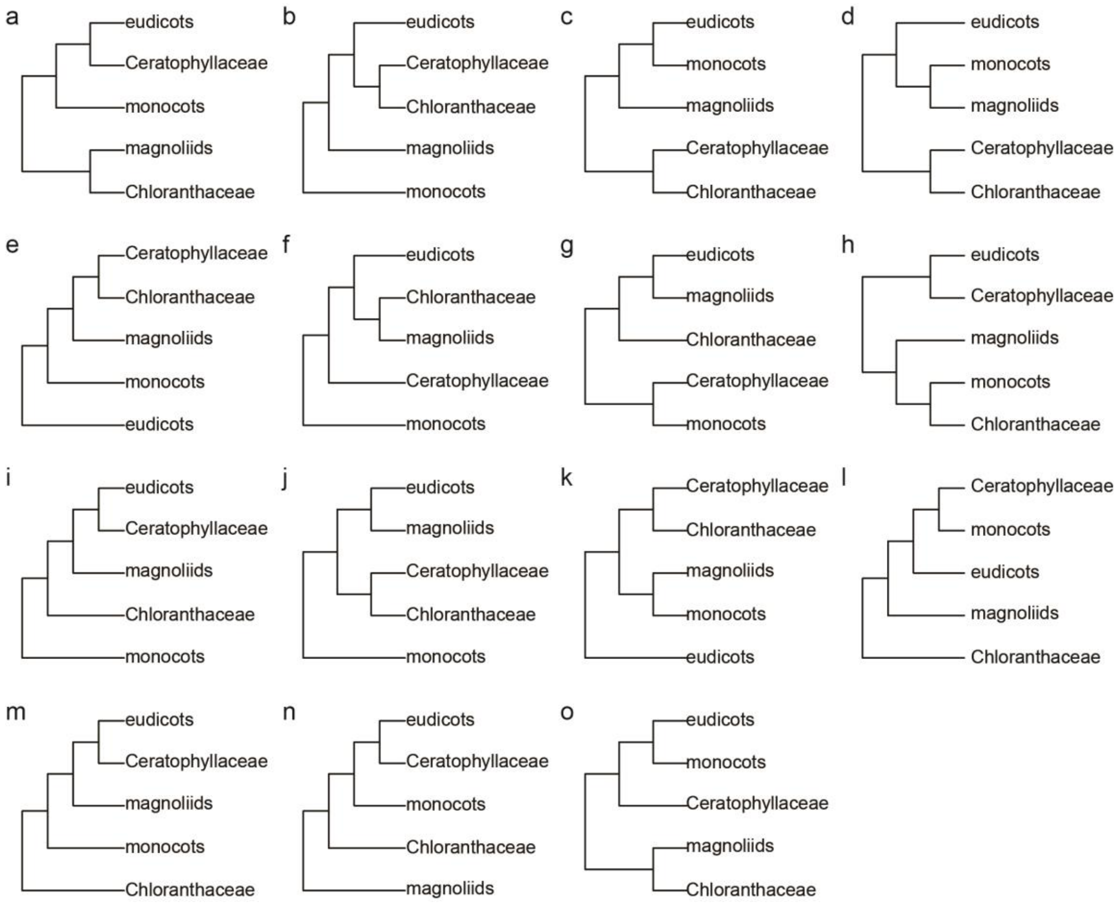

Supplementary Figure 1. The previously estimated alternative topologies within angiosperms. (a) refs 13-15; (b) ref 16; (c) ref 17; (d) ref 18; (e) ref 19; (f) ref 20; (g) refs 21-23; (h) refs 21,24; (i) ref 21; (j) ref 21; (k) ref 21; (l) refs 15,21; (m) ref 25; (n) ref 14; (o) ref 26.

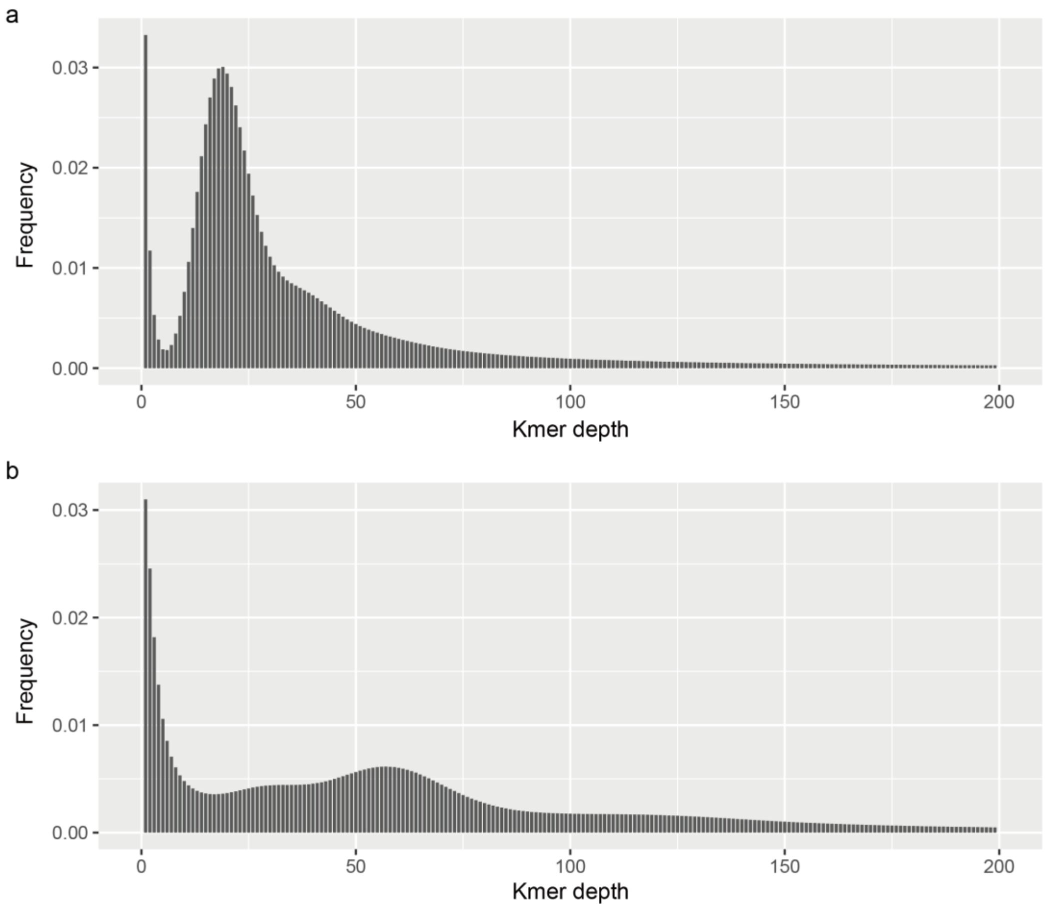

Supplementary Figure 2. 17-mer-based analysis to estimate the genome size of prickly waterlily (a) and hornwort (b) respectively.

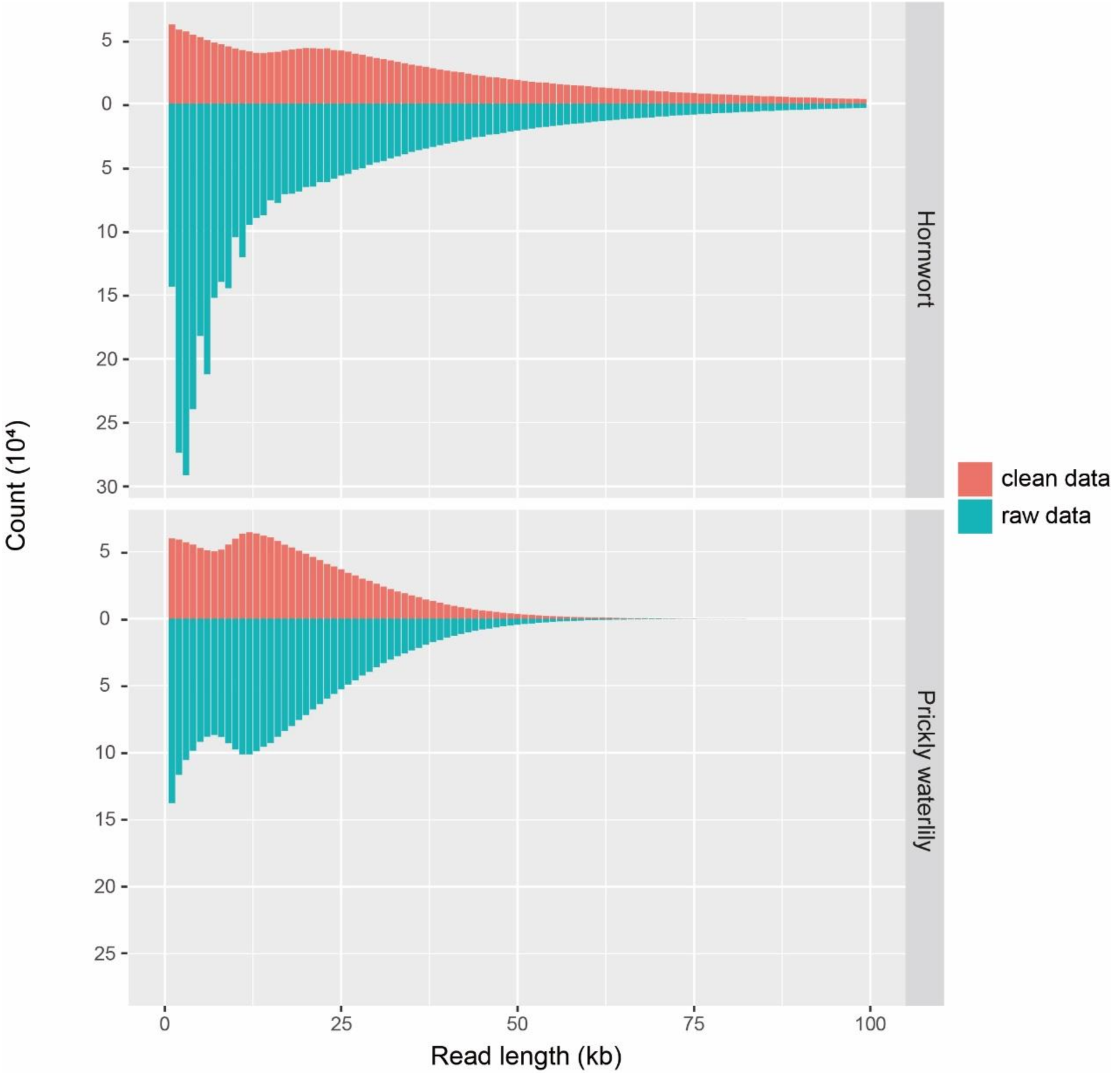

Supplementary Figure 3. The length distribution of ONT long reads. The blue bars represent the raw sequencing data and the red bars represent the cleaned data, which removed those reads with the mean quality score < 7.

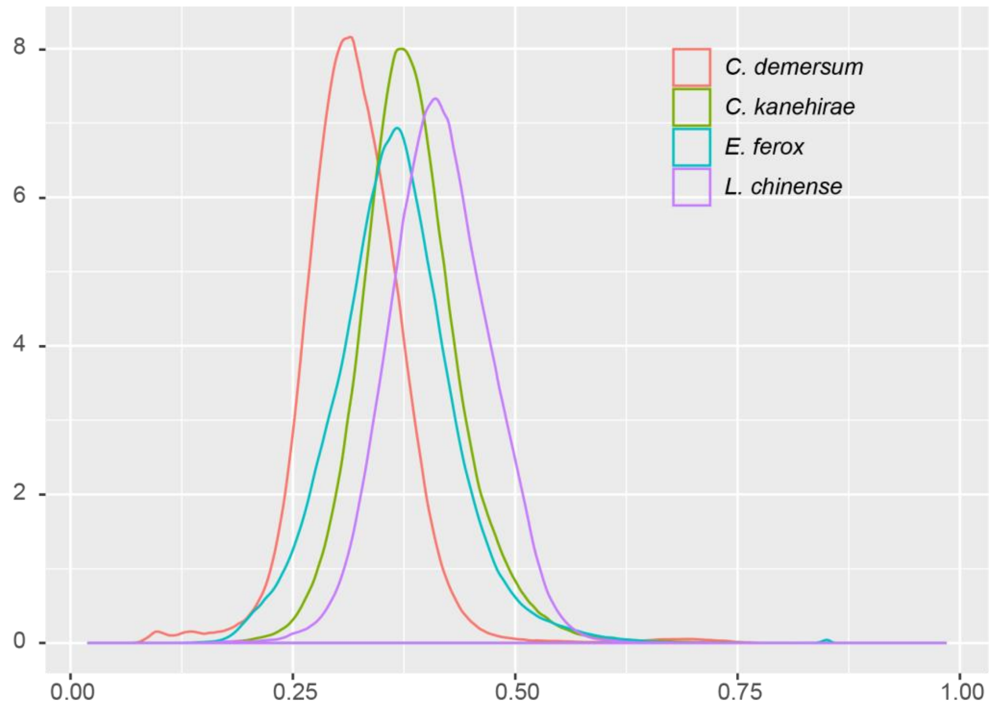

Supplementary Figure 4. The GC content distribution for the two species. The non-overlapping window size is 500bp.

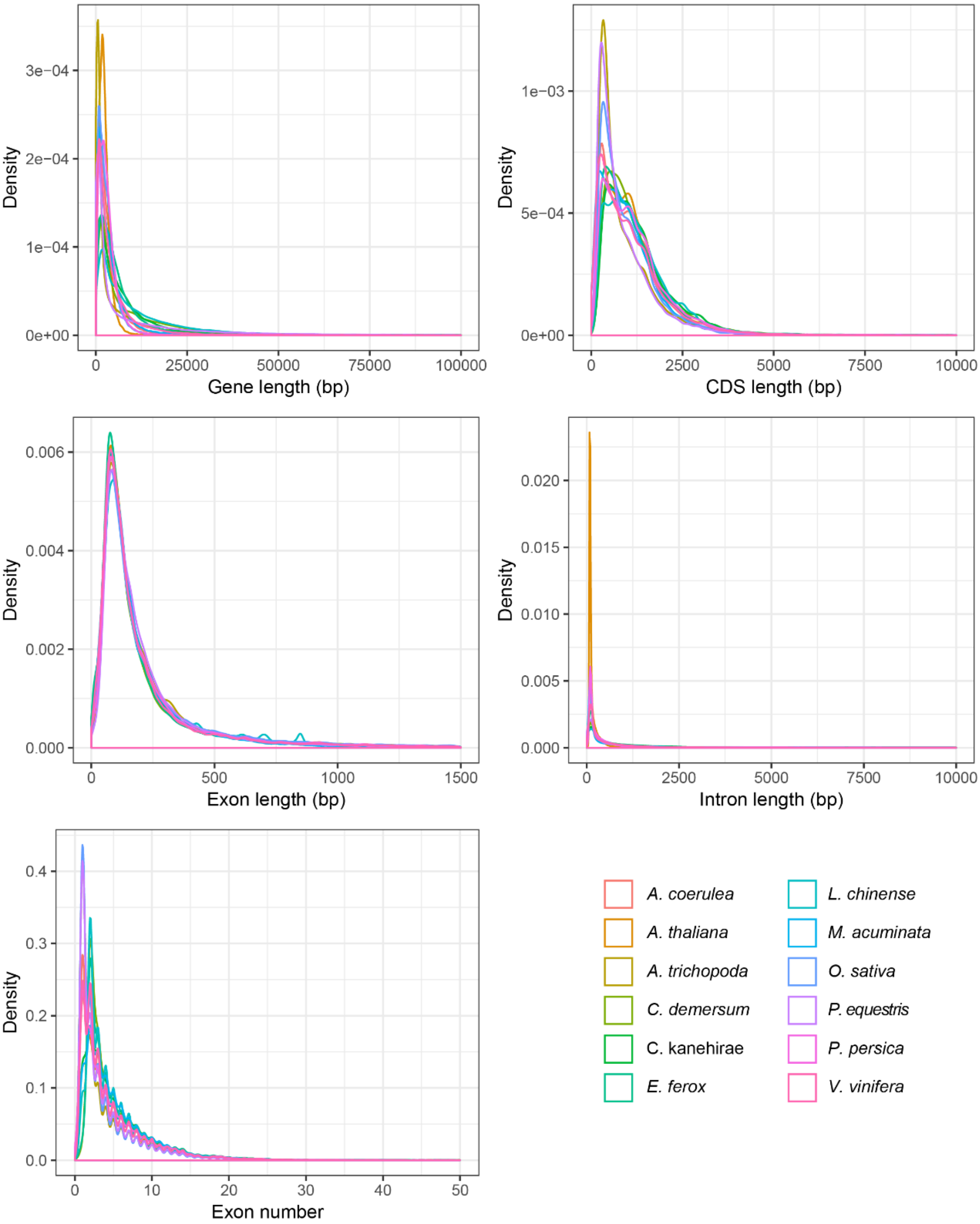

Supplementary Figure 5. Comparison of mRNA length (a), CDS length (b), Exon length (c), Intron length (d), and Exon number per gene (e) among the 12 angiosperm species. The x-axis represents length or number and the y-axis represents the density of genes.

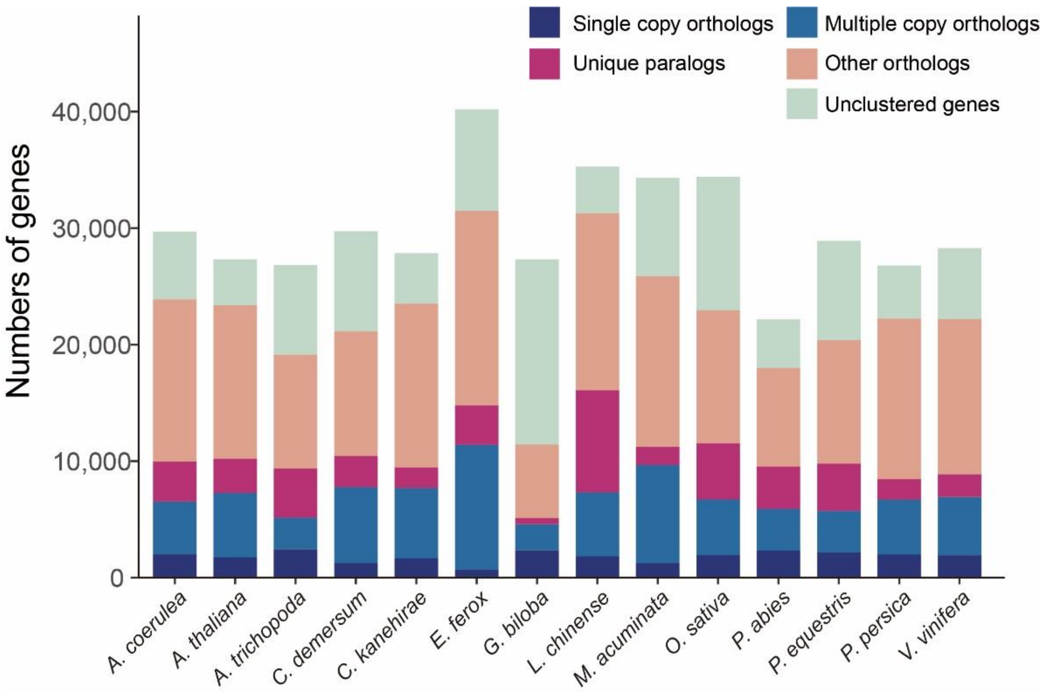

Supplementary Figure 6. The histogram of Gene orthology among 14 species.

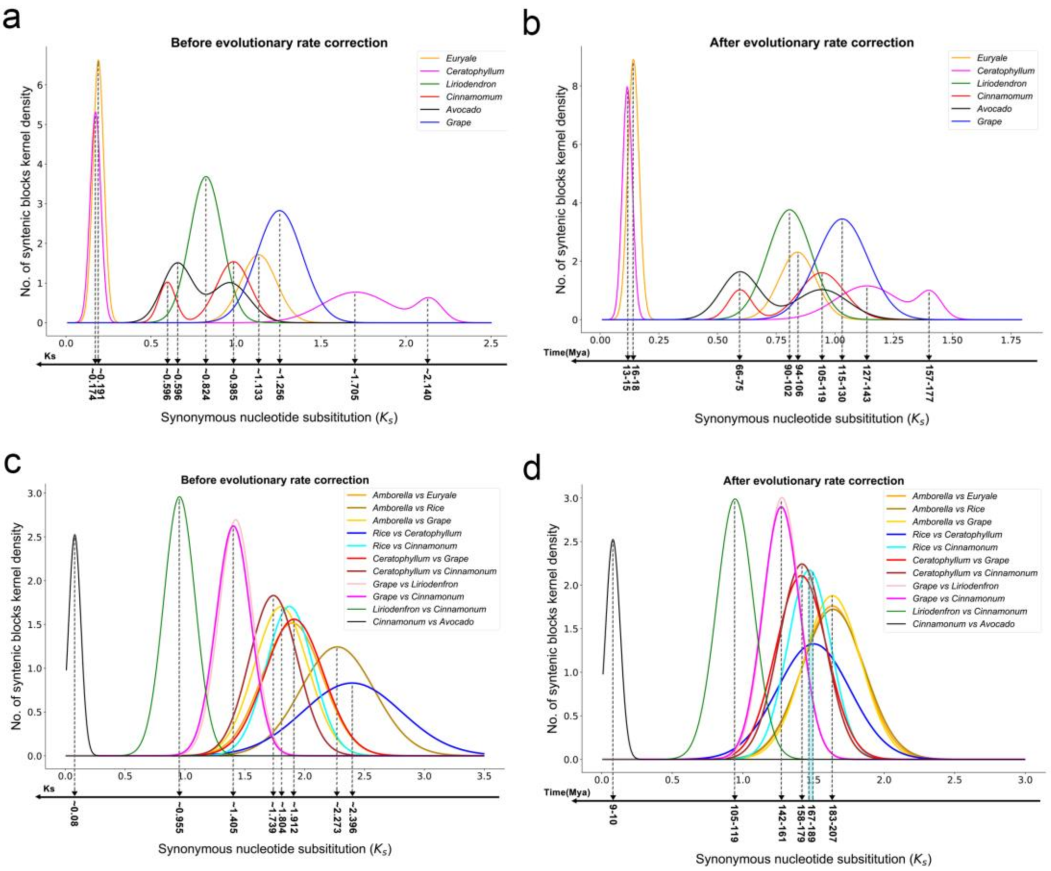

Supplementary Figure 7. The distribution of synonymous nucleotide substitutions (Ks) between collinear genes. (a) and (b) were showing the raw and corrected Ks distribution within one species (representing the polyploidization events), respectively. (c) and (d) were showing the raw and corrected Ks distribution between each two species (representing the speciation events).

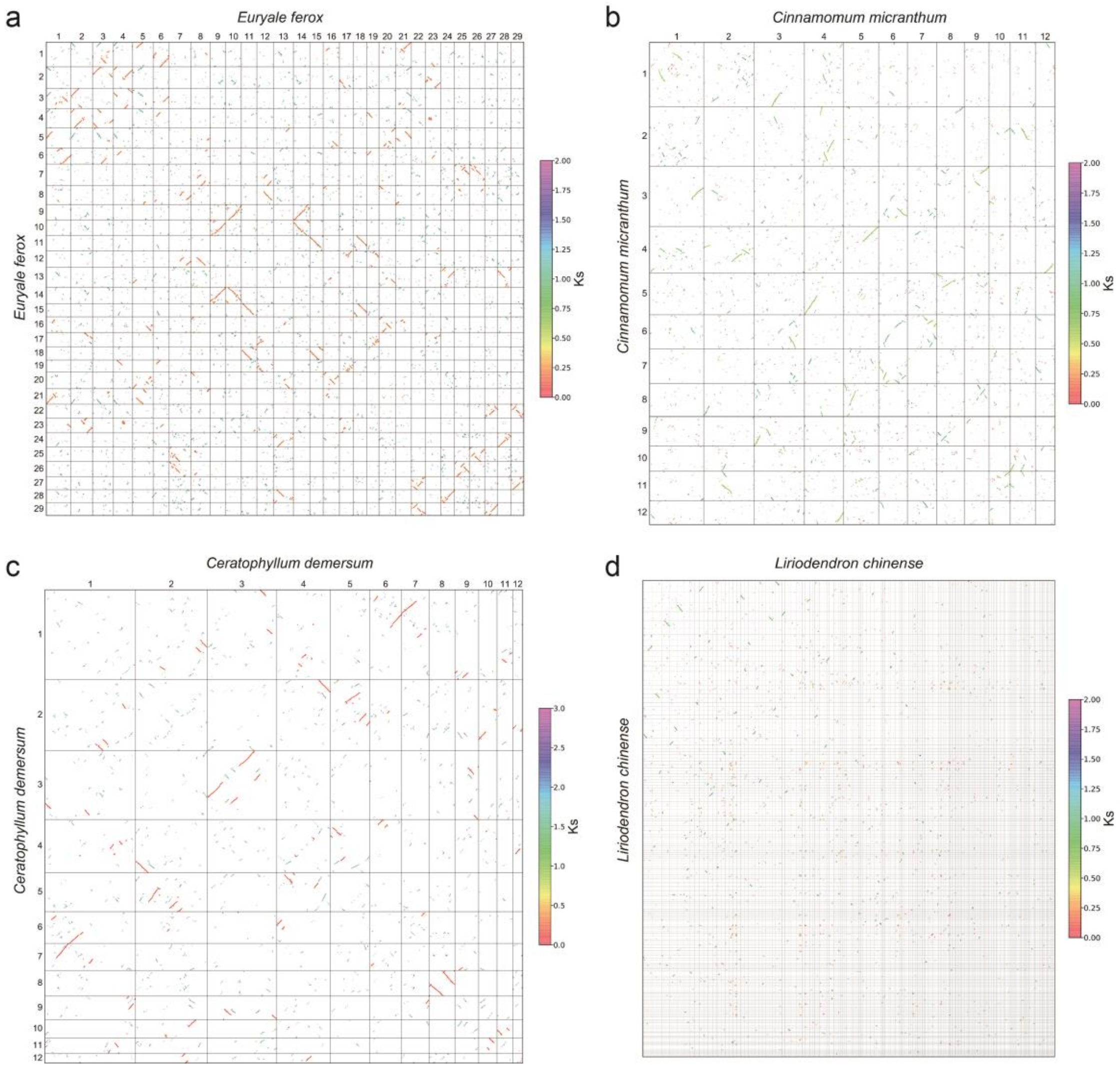

Supplementary Figure 8. Syntenic block dotplot within *Euryale, Ceratophylum, Lirioderon* and *Cinnamomum*. All the blocks should contain ≥ 5 syntenic gene pairs and the x and y axis represent the chromosomes, excepted the *Liriodendron* representing the scaffold and ordered by the scaffold's length. The color representing the mean Ks values of each syntenic block.

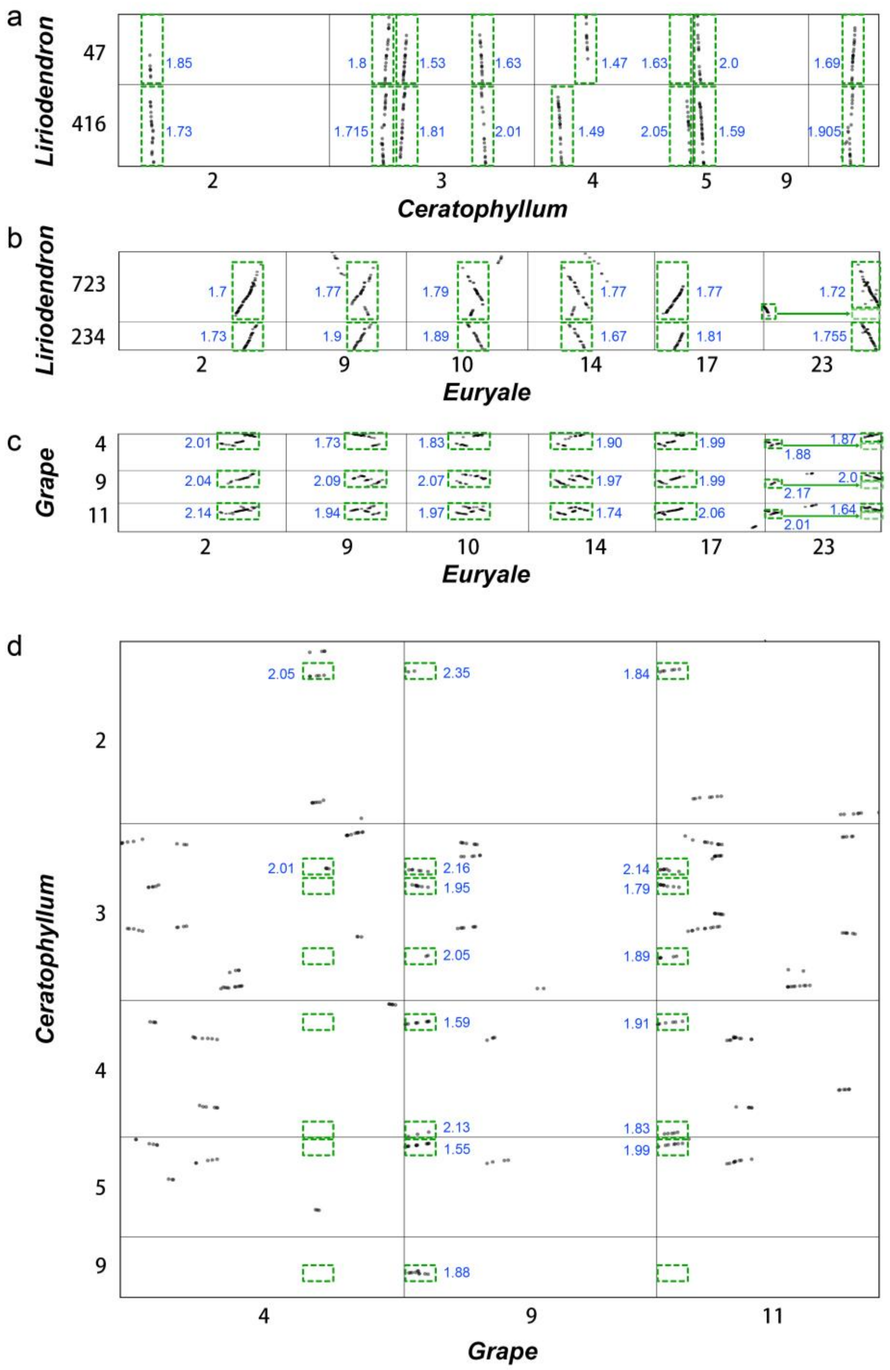

Supplementary Figure 9. Syntenic block dotplot among *Euryale, Ceratophylum, Lirioderon* and Grape. All the blocks should contain ≥ 10 syntenic gene pairs and the blue number along the dashed box representing the mean KS value of the syntenic block. (a) *Lirioderon – Ceratophylum* with the syntenic depth ratio of 2:8; (b) *Lirioderon – Euryale* with the syntenic depth ratio of 2:6; (c) Grape – *Euryale* with the syntenic depth ratio of 3:6; (d) Grape – *Ceratophylum* with the syntenic depth ratio of 3:8. The empty dashed boxes representing the candidate existing syntenic blocks, which may have lost after the polyploidization.

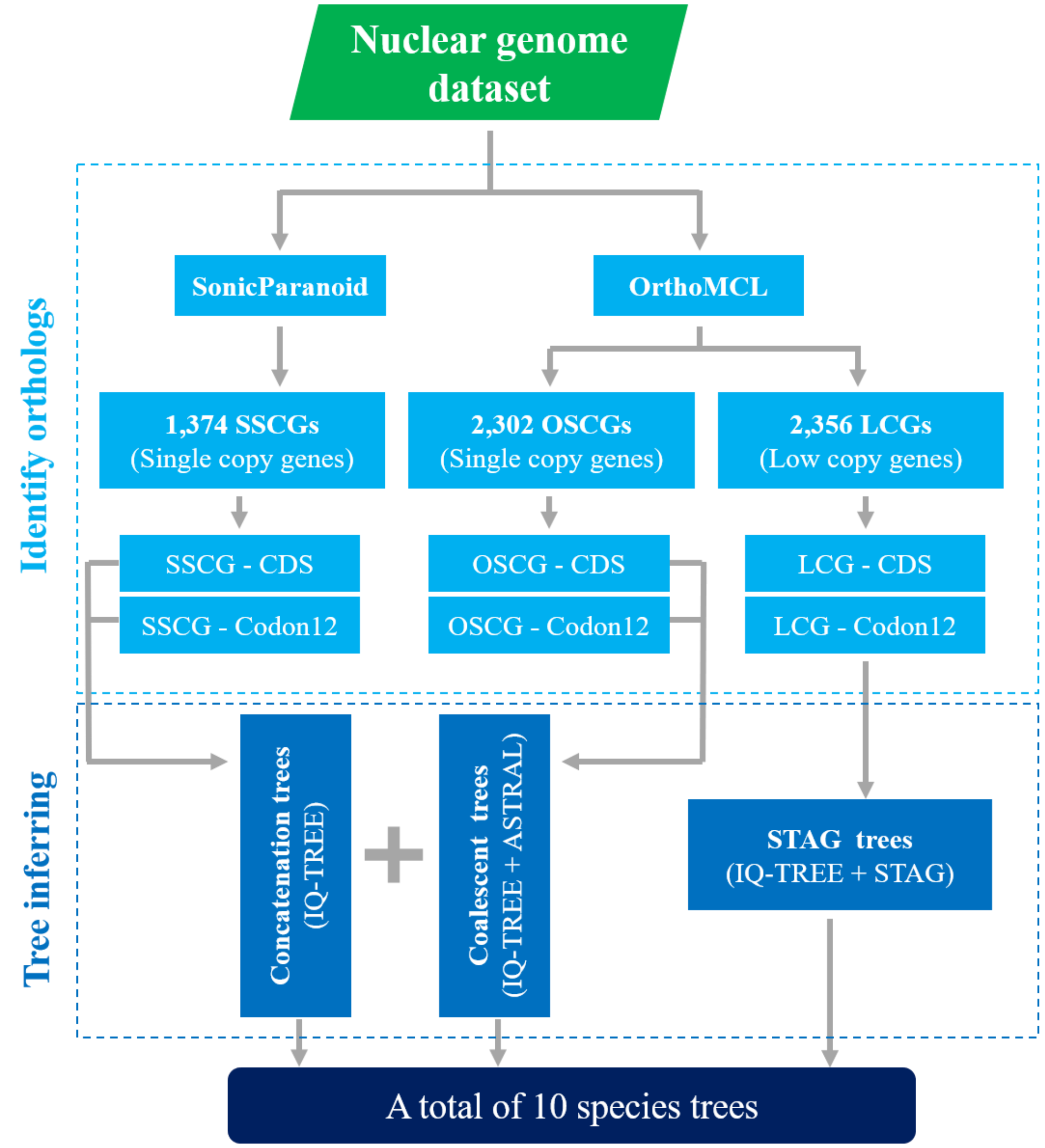

Supplementary Figure 10. The phylogeny analysis pipeline. SSCGs representing the single copy genes inferred by the SonicParanoid among the 14 species. OSCGs representing the single copy genes inferred by OrthoMCL among the 15 species, and only the SCGs containing more than 11 species were retained. LCGs representing the low copy gene families among the 15 species, and for each species containing less than five sequences.

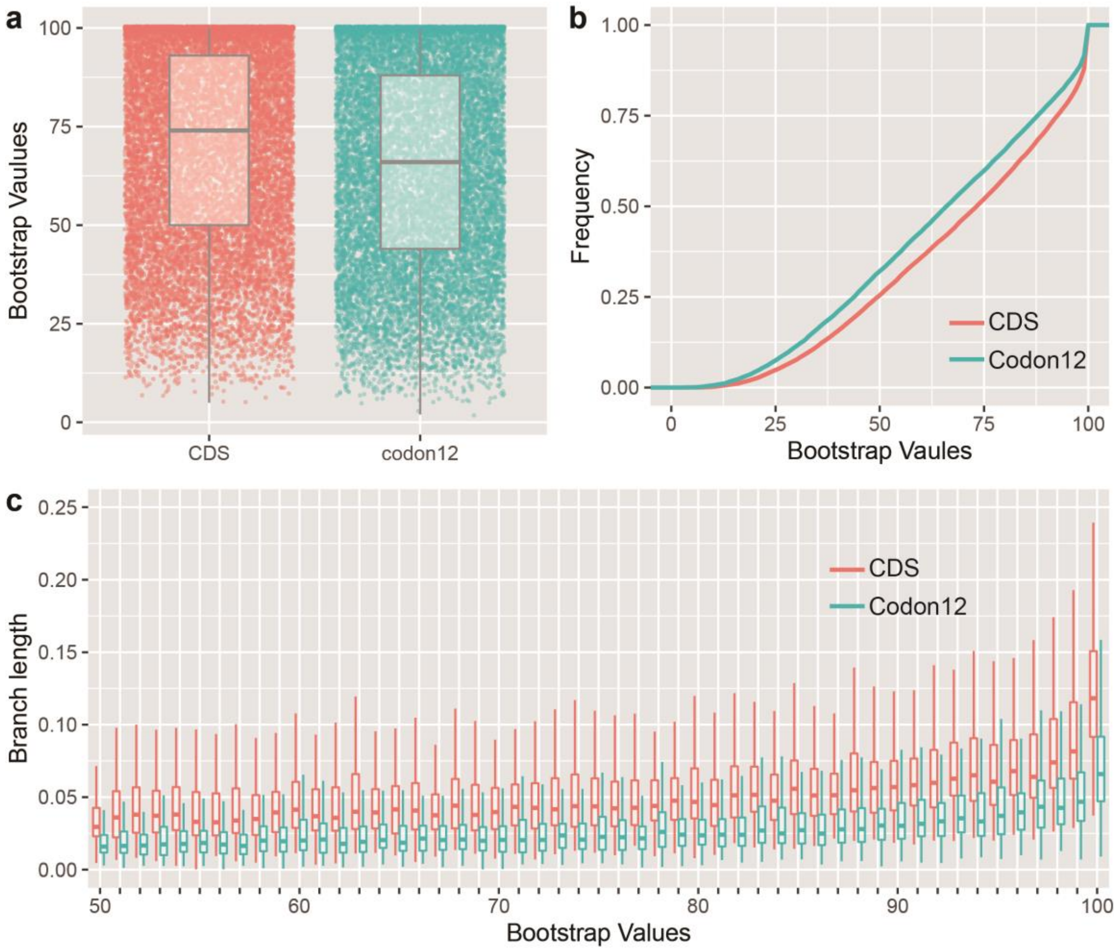

Supplementary Figure 11. Bootstrap support (BS) graphed against branch length within the 1,374 raw SSCGs trees. (a) The BS of each internal branch among the two datasets: CDS and Codon12. The paired sample t-test (two tailed, n=15,114 internal branches) were applied to test the BS difference between the CDS and Codon12, and a *P* value < 2.2e-16 were obtained. (b) Cumulative length distribution of the BS of internal branches. A total of 75.5% and 49.1% of the branches received a BS values greater than 50% and 75% in SCG-CDS dataset, respectively. A total of 68.9% and 41.2% of the branches received a BS values greater than 50% and 75% in SCG-CDS dataset, respectively. (c) The distribution of branch length within each bootstrap value (from 50 to 100) within the total of 1,374 SSCGs trees from CDS and Codon12 datasets. For each box plot the sample size n = the total number of branches with the corresponding BS values. We also found the significant correlation between the bootstrap (only considered the BS values ≥ 50) and branch length (SSCG-CDS: Spearman *P* < 0.01; SSCG-Codon12: Spearman *P* < 0.01). For the box plot, the line in the center of the box represent the median values, the edges of the box represent the first and third quartiles, and the whiskers above and below the box show the range of values.

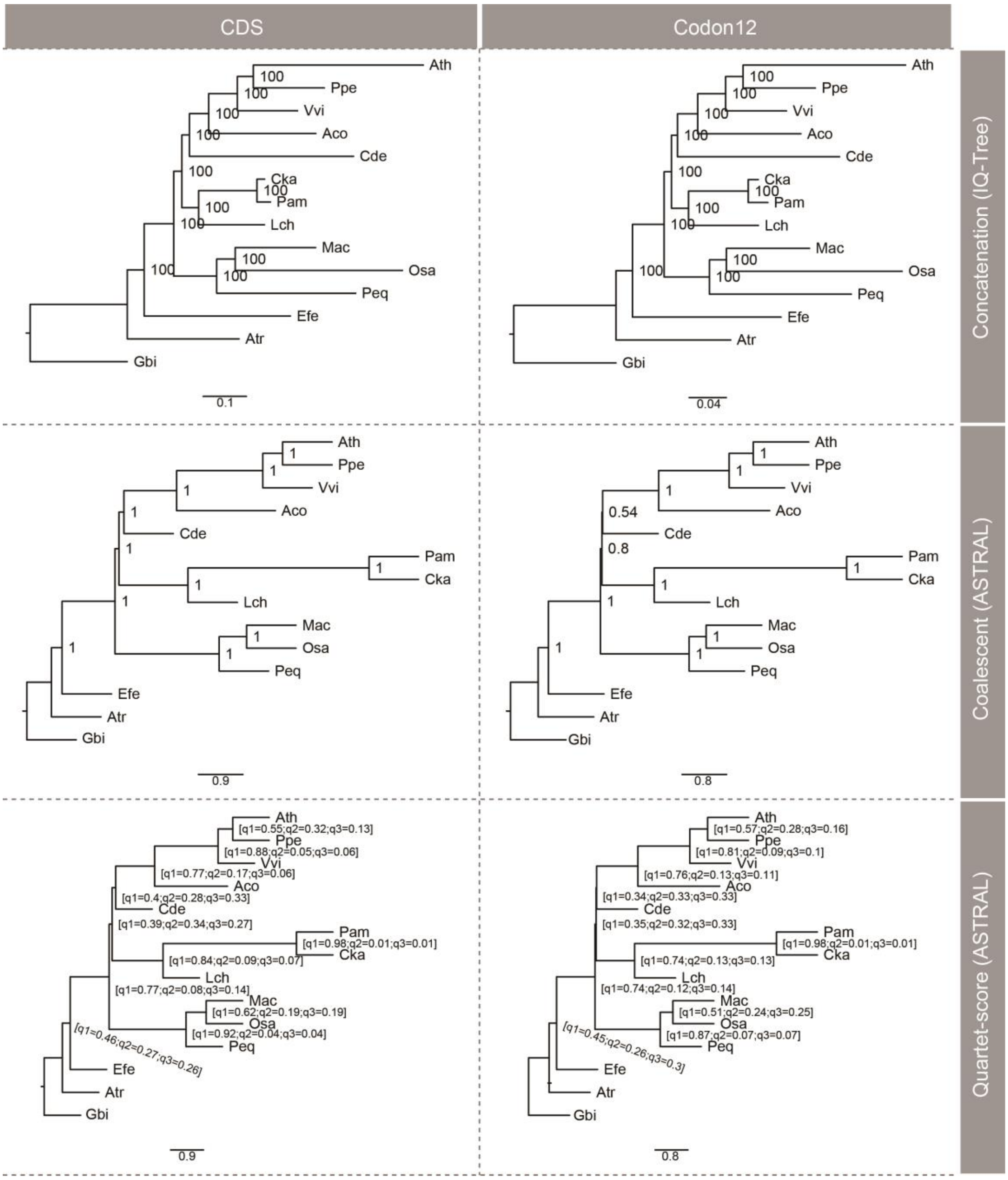

Supplementary Figure 12. Phylogenetic relationships inferred with the 1,374 SSCGs. The corresponding relationship between abbreviations and full names are listing as follow: Aco - *A. coerulea*, Ath - *A. thaliana*, Atr - *A. trichopoda*, Cde - *C. demersum*, Cka - *C. kanehirae*, Efe - *E. ferox*, Gbi - *G. biloba*, Lch - *L. chinense*, Mac - *M. acuminata*, Osa - *O.sativa*, Pam – *P. americana*, Peq - *P. equestris*, Ppe - *P. persica*, Vvi - *V. vinifera*.

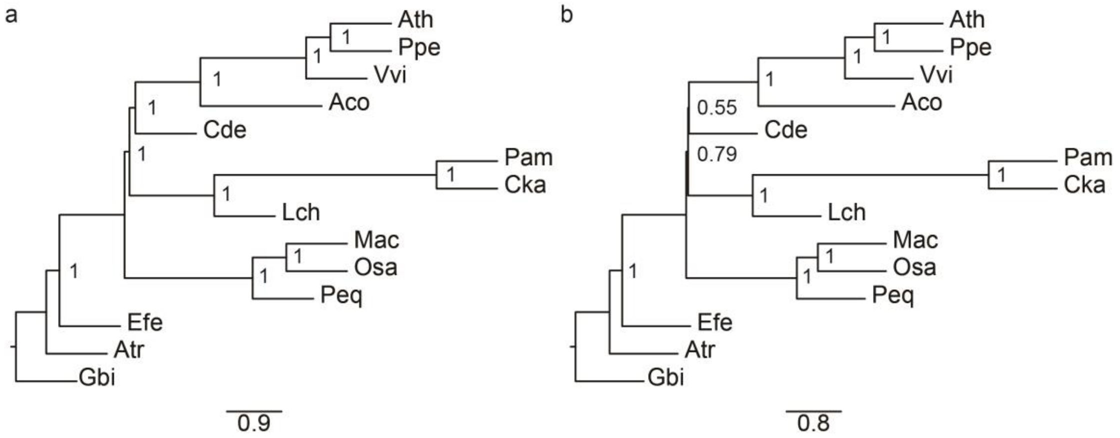

Supplementary Figure 13. ASTRAL tree inferred with the 1,374 SSCGs and removed the Bootstrap values less than 10. (a) Tree inferred with the SSCG-CDS dataset. (b) Tre inferred with the SSCG-Codon12 dataset. The corresponding relationship between abbreviations and full names are listing as follow: Aco - *A. coerulea*, Ath - *A. thaliana*, Atr - *A. trichopoda*, Cde - *C. demersum*, Cka - *C. kanehirae*, Efe - *E. ferox*, Gbi - *G. biloba*, Lch - *L. chinense*, Mac - *M. acuminata*, Osa - *O.sativa*, Pam – *P. americana*, Peq - *P. equestris*, Ppe - *P. persica*, Vvi - *V. vinifera*.

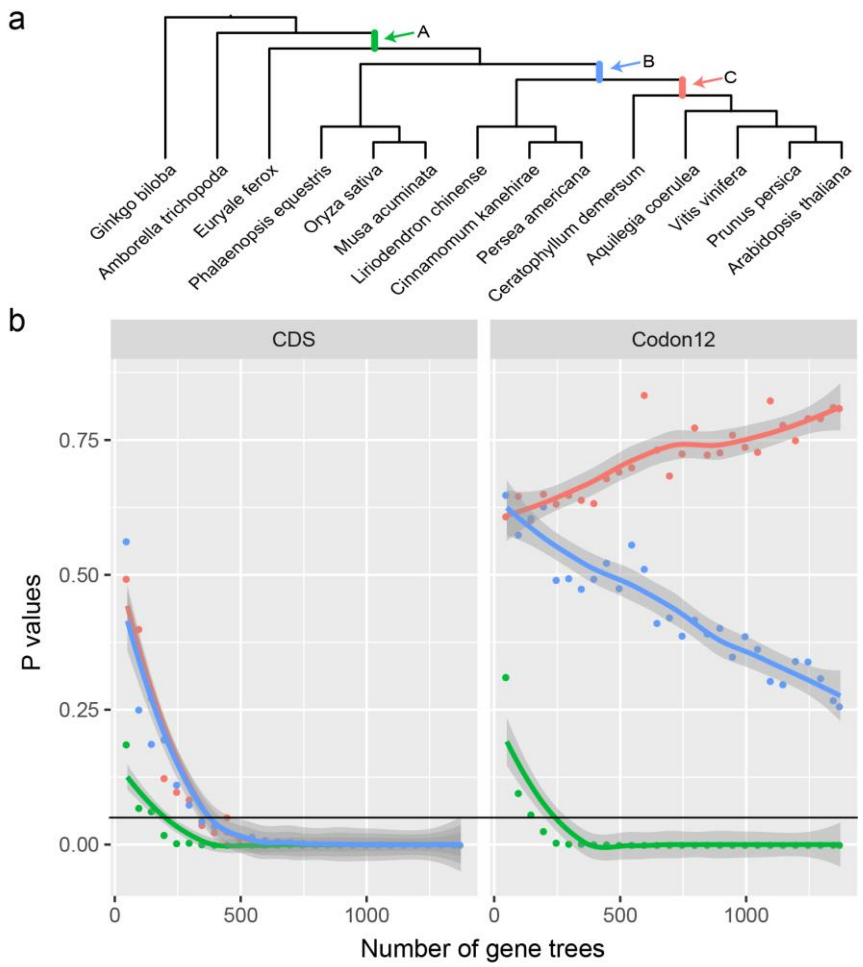

Supplementary Figure 14. Polytomy test results on the three not exactly supported (BS < 100 or PP < 1) branches among the coalescent / concatenation (1,374 SSCGs). (a) represented the topology of the identical nuclear DNA tree and the three branches were marked as green, blue and red. (b) represented the changing in *P*-values with respect to the number of genes for the selected branches in the corresponding species tree. We used ASTRAL species trees with the varying number of gene trees sampled uniformly (50, 100, 150 … 1,300 SSCG trees), and repeated 20 times. We show average p-values (y-axis) versus the number of gene trees (y-axis). The null hypothesis is that a branch in an estimated species tree should be replaced by a polytomy. The y axis represent the *P* values generated by the Chi-square test with the number of gene trees supporting the three alternative topologies, and the sample size n = the corresponding gene tree number at each points. The solid horizontal line shows *P*-value = 0.05. We could find that all the three branches were quickly reject the null hypothesis with the number of gene trees adding in the SSCG-CDS dataset. While, within SSCG-Codon12 dataset, only A branch could reject the null hypothesis, B branch need more number of gene trees and C branch was more complicated.

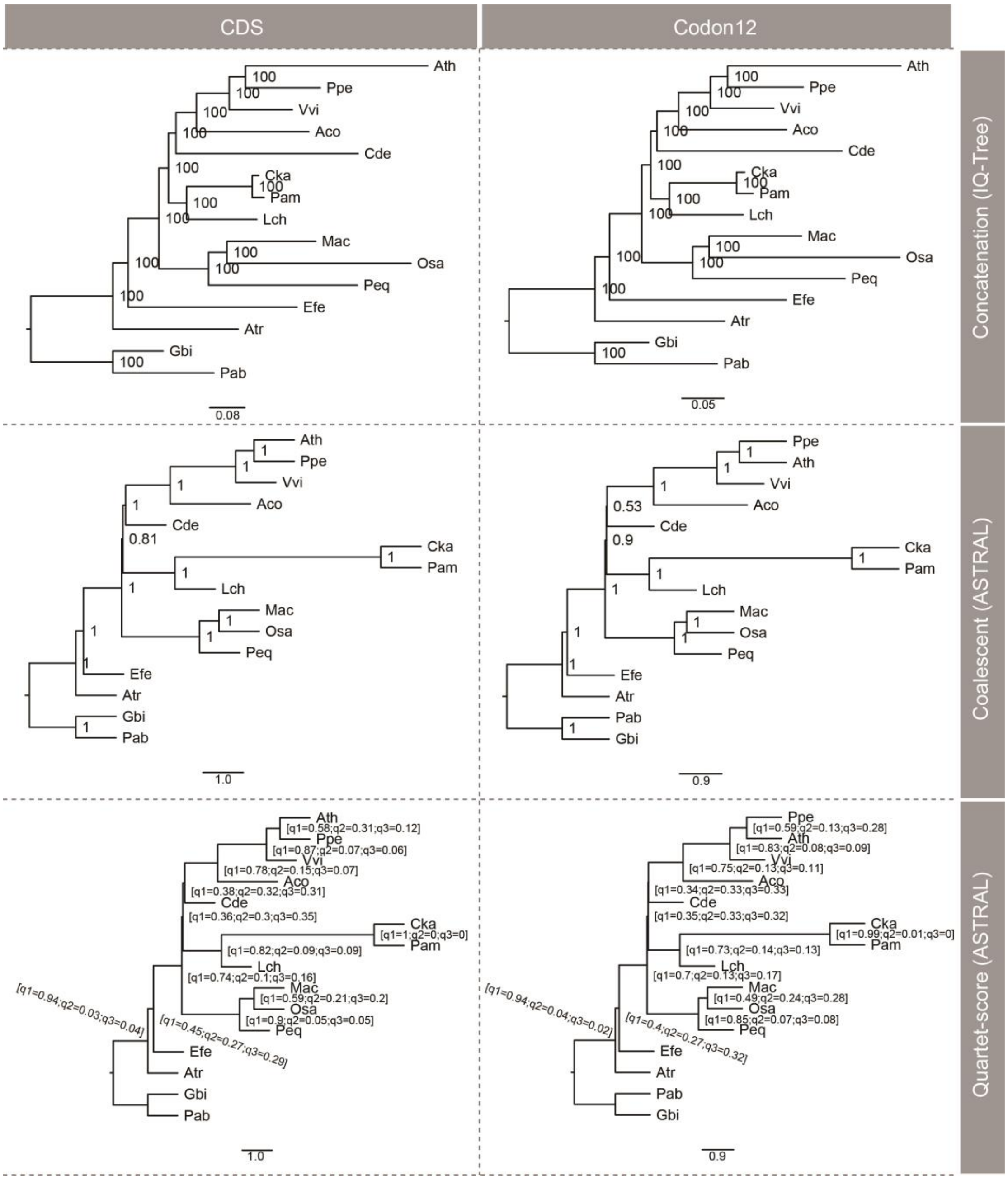

Supplementary Figure 15. Phylogenetic relationships inferred with the 2,302 OSCGs. The corresponding relationship between abbreviations and full names are listing as follow: Aco - *A. coerulea*, Ath - *A. thaliana*, Atr - *A. trichopoda*, Cde - *C. demersum*, Cka - *C. kanehirae*, Efe - *E. ferox*, Gbi - *G. biloba*, Lch - *L. chinense*, Mac - *M. acuminata*, Osa - *O.sativa*, Pam – *P. americana*, Pab - *P. abies*, Peq - *P. equestris*, Ppe - *P. persica*, Vvi - *V. vinifera*.

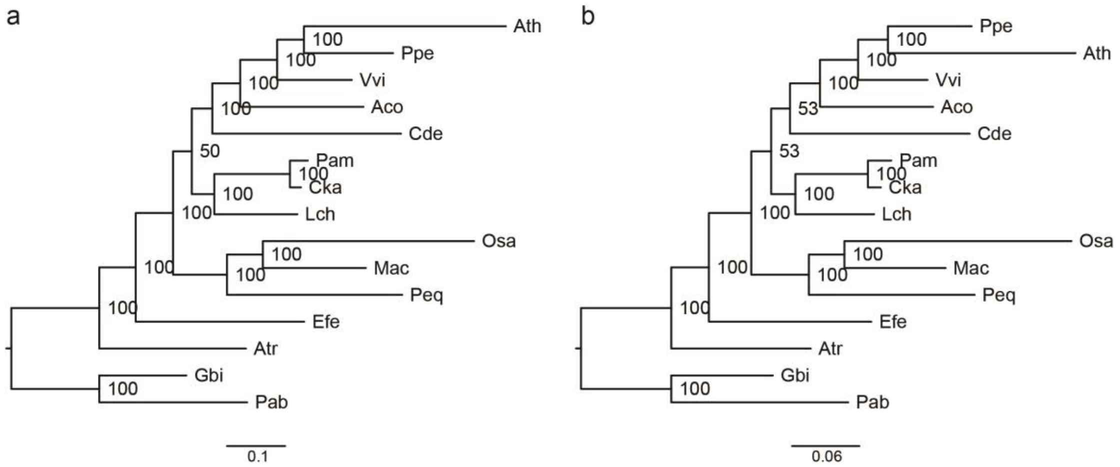

Supplementary Figure 16. Phylogenetic relationships inferred by STAG and the low-copy orthogroups (2,356 gene families). The tree topologies were estimated by all the 2,356 low copy gene family trees. The corresponding bootstrap were conculcated as follow: randomly selected one tree from the bootstrap trees of each gene family results and then running STAG to generate one bootstrap, and repeat 100 times. The two phylogenetic trees: (a) and (b) represent the CDS and Codon12, respectively. The corresponding relationship between abbreviations and full names are listing as follow: Aco - *A. coerulea*, Ath - *A. thaliana*, Atr - *A. trichopoda*, Cde - *C. demersum*, Cka - *C. kanehirae*, Efe - *E. ferox*, Gbi - *G. biloba*, Lch - *L. chinense*, Mac - *M. acuminata*, Osa - *O.sativa*, Pam – *P. americana*, Pab - *P. abies*, Peq - *P. equestris*, Ppe - *P. persica*, Vvi - *V. vinifera*.

a

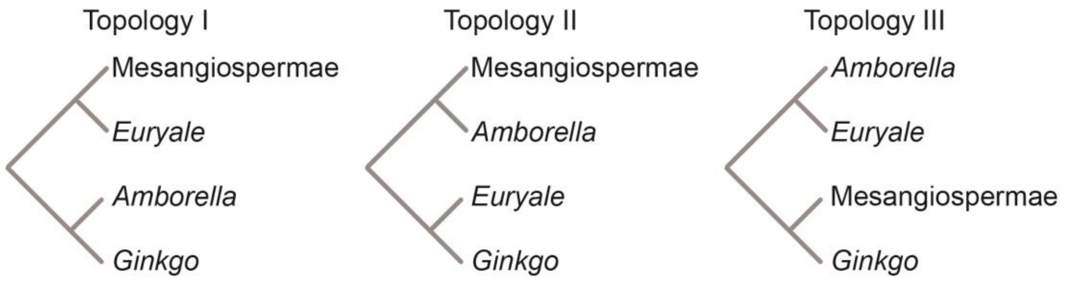

Dataset	Topology I	Topology II	Topology III	Chi-squared test
CDS	636	374	364	103.88 (*P*<2.2e-16)
Codon 12	612	356	406	80.40 (*P*<2.2e-16)

b

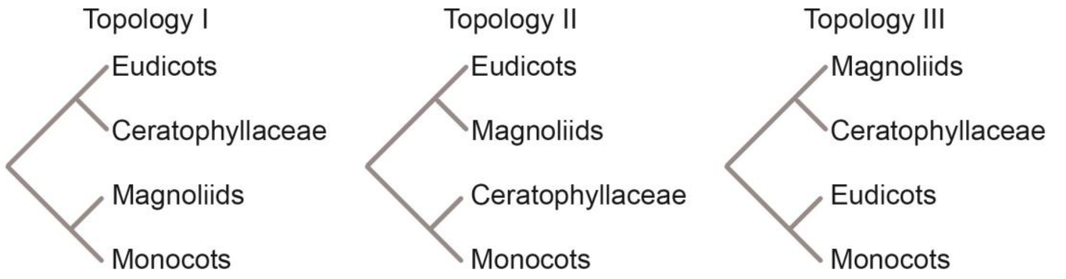

Dataset	Topology I	Topology II	Topology III	Chi-squared test
CDS	545	380	449	29.99 (*P*=3.08e-10)
Codon 12	470	452	452	0.40 (*P*=0.79)

Supplementary Figure 17. Summary of the gene tree count for supporting the alternative topologies (1,374 SSCGs). (a) Representing the three alternative topologies within the basal angiosperms. (b) The three topologies among the four Mesangiospermae groups. All the tree counts were calculated by ASTRAL with the parameter '-t=2'.

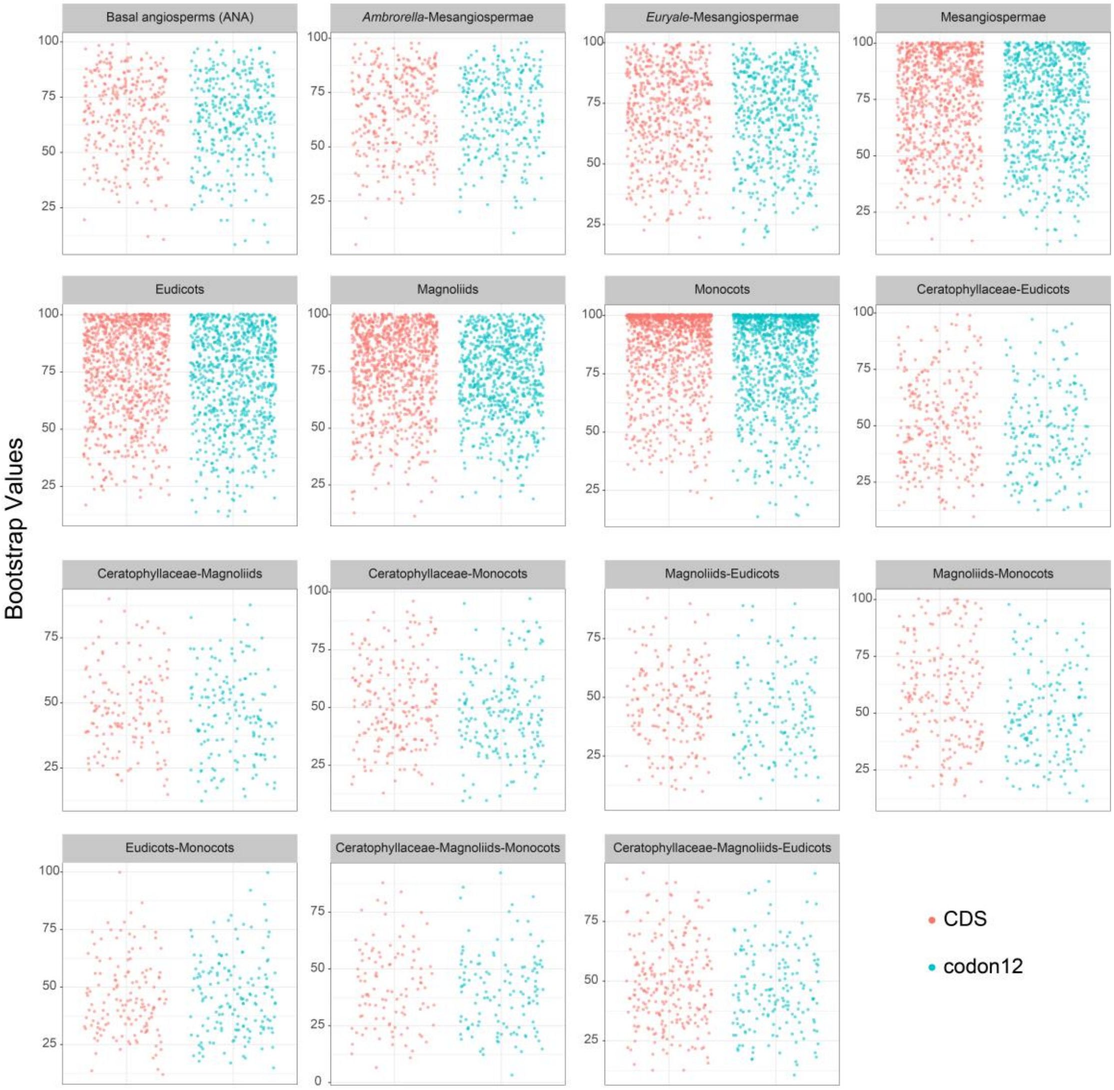

Supplementary Figure 18. The distribution of bootstrap values supporting for monophyletic group (1,374 SSCGs datasets).

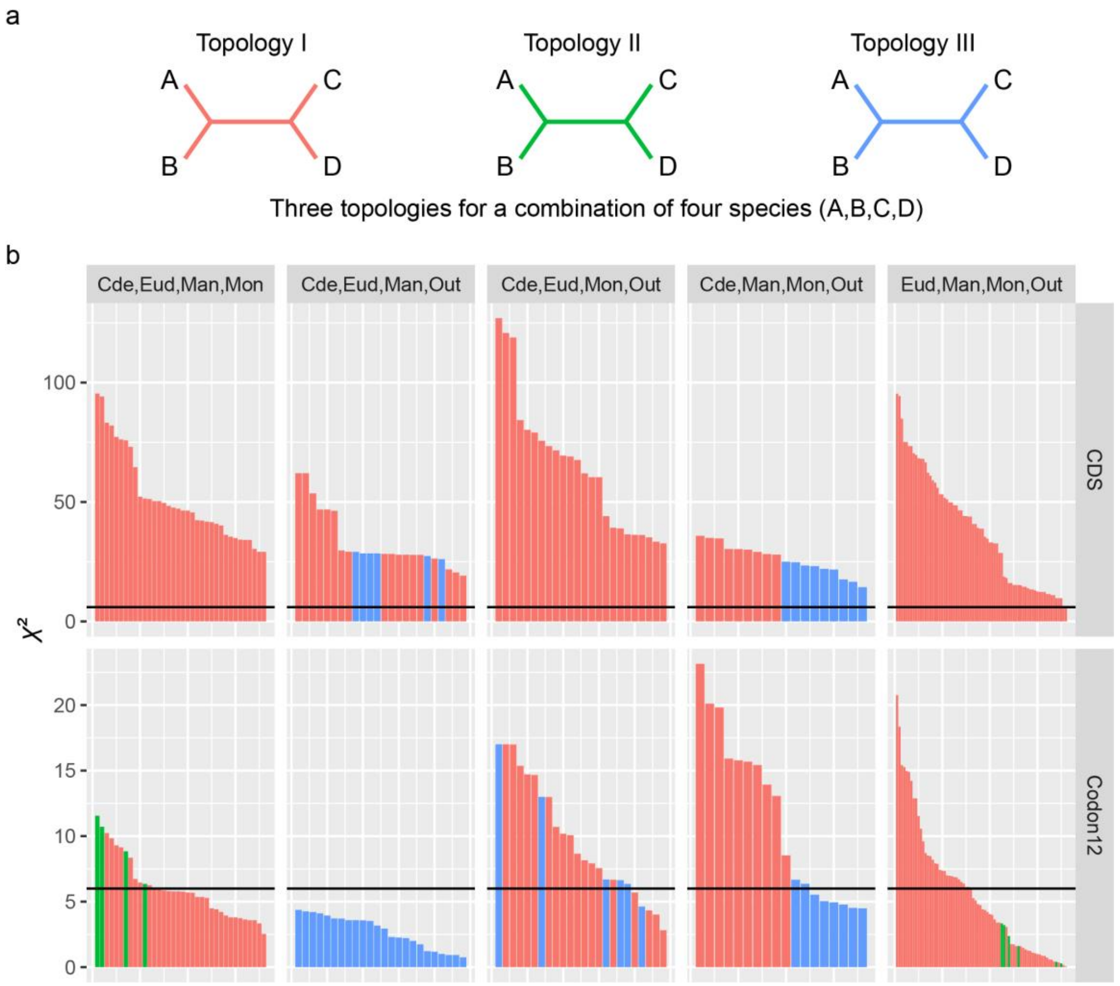

Supplementary Figure 19. Summary of the gene tree distribution of three topologies within the four Mesangiospermae groups (1,374 SSCGs). (a), The three possible topologies for the four species ((A, B), (C, D)), ((A, C), (B, D)) and ((A, D), (C, B)). (b), The corresponding quartet tree counting result. The y axis is the Chi-Squared χ^2 values calculated between the three topologies, and the n=the total number of observed trees for each four species combination. The line y=5.9915 represent the *P*=0.05 threshold. The x axis represents all the combination of four species from the corresponding four groups. Cde representing Ceratophyllaceae, Eud representing eudicots, Mag represent magnoliids, Mon represent monocots and Out represent the basal angiosperms and gymnosperms.

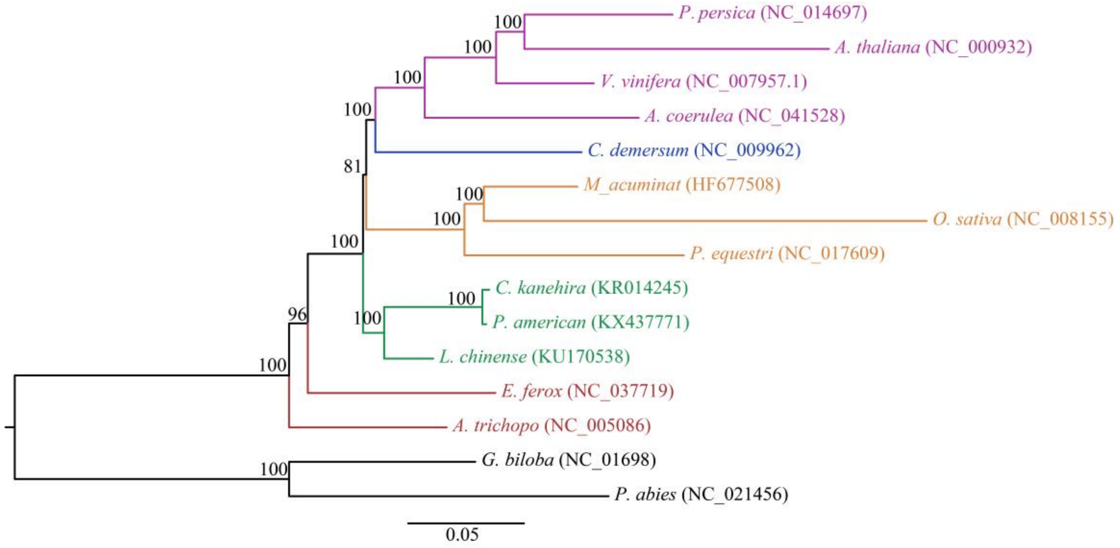

Supplementary Figure 20. The Maximum Likelihood (ML) tree inferred based on the 72-chloroplast gene' coding sequences from the 15 species.

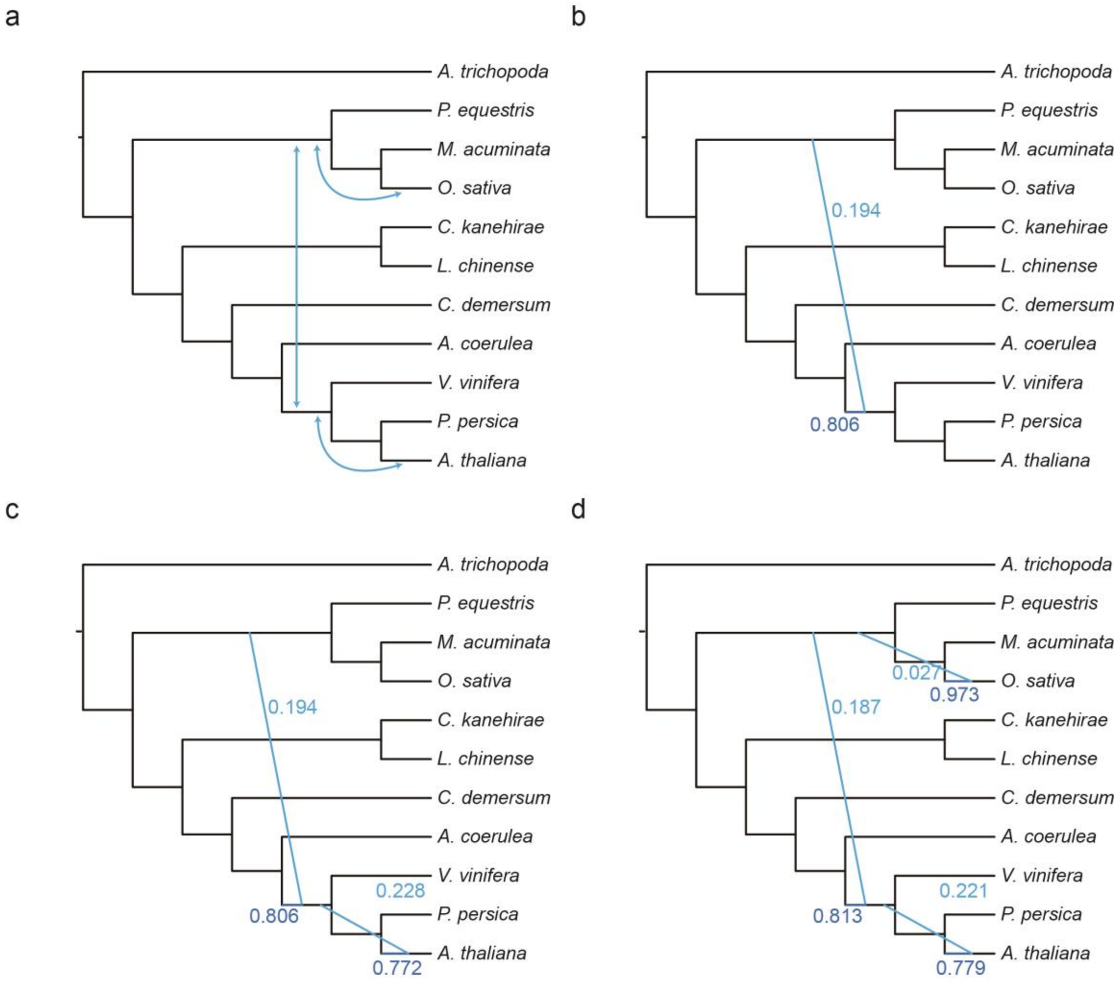

Supplementary Figure 21. The likely hybrid events among the 13 species that inferred by PhyloNetworks. The blue lines represent the predicted hybrid events and the numbers on the blue line represent the proportion of mixture. (a) Representing the simplified summary of the hybridizations. (b)-(d) Representing the number of hybridizations from 1-3.

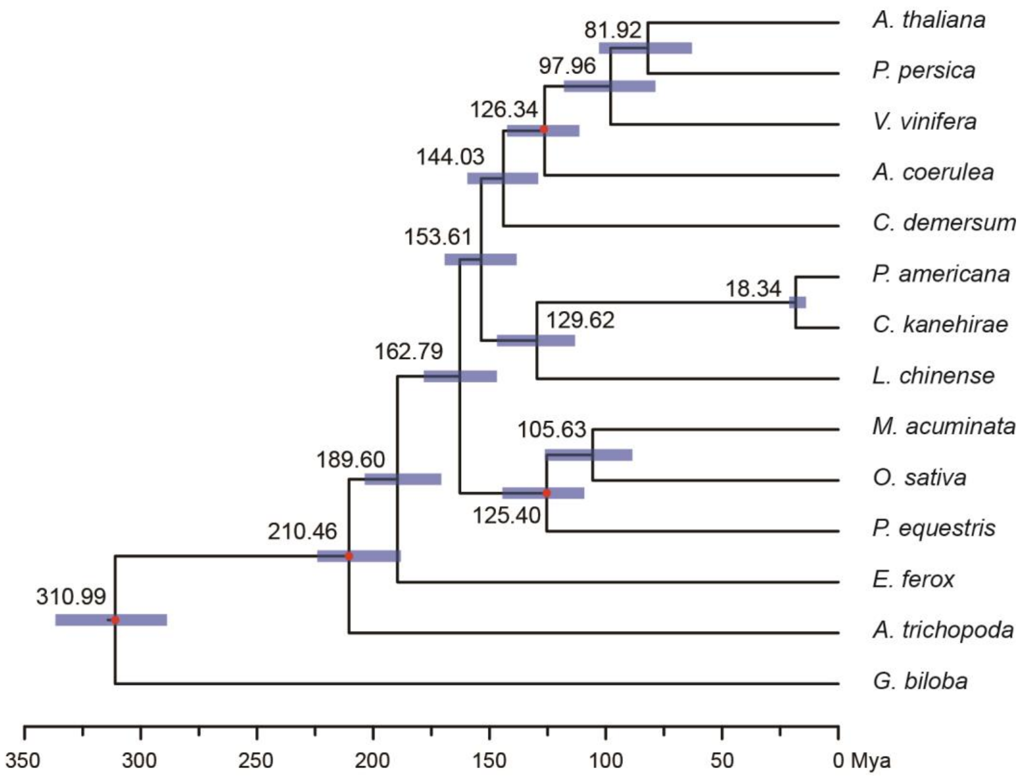

Supplementary Figure 22. Divergence time estimated among 14 species. This tree topology was generated by MCMCtree and the CDS sequences. The red dots correspond to calibration points as mentioned in the method. Divergence estimates (Mya, million years ago) are indicated above each node and the blue nodal bars show the 95% confidence intervals (n=20,000 independent samples).

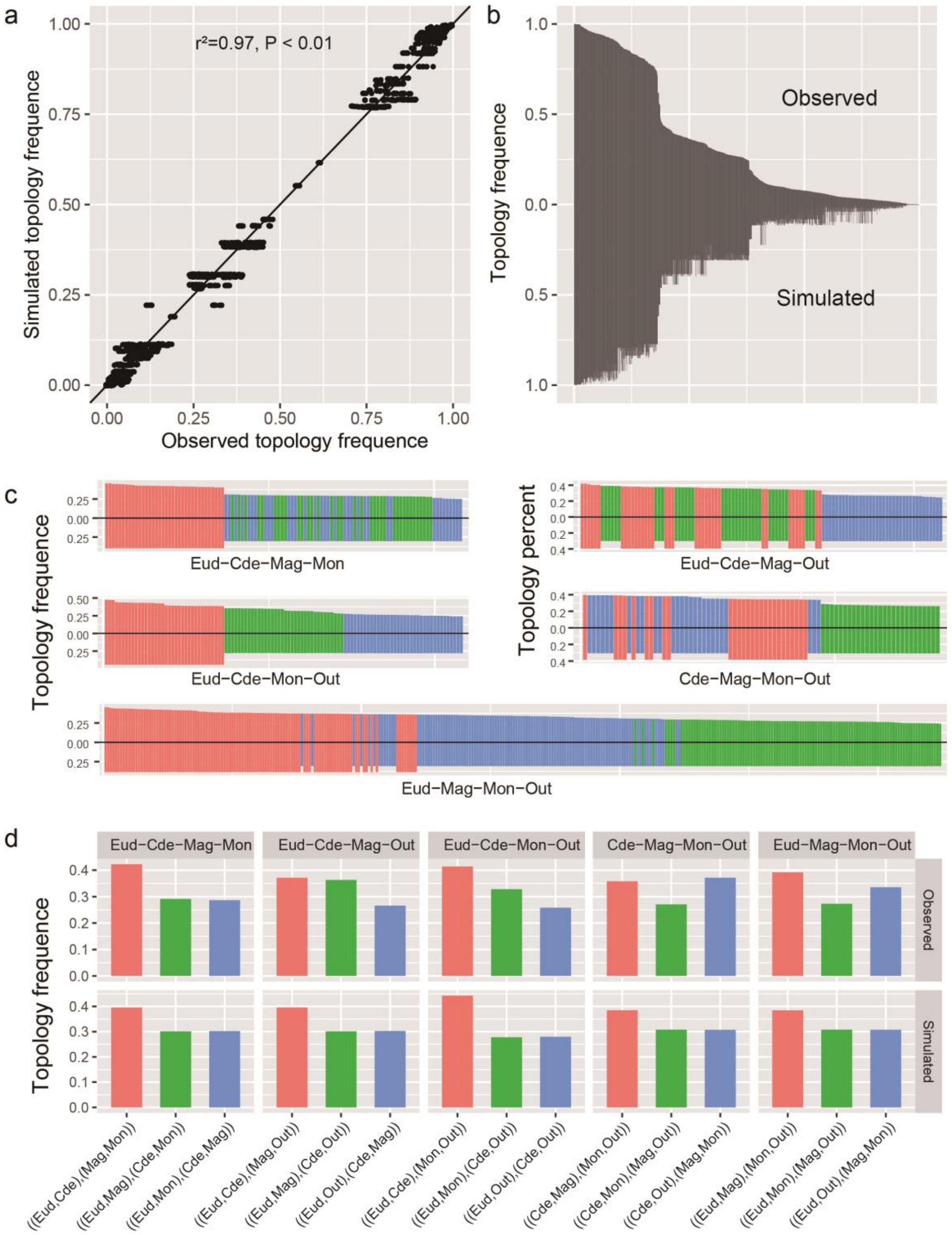

Supplementary Figure 23. Tree count numbers between observed CDS gene trees and the corresponding simulated trees within each 4 species. We simple the topology frequency within all the possible four species, and calculated the frequencies of each three topologies for each four species combination of all the 14 species. All the bar plot above the y=0 represent the observed frequencies and below y=0 represent the simulated frequencies. (a) Comparison of simulated frequencies based on coalescent probabilities of gene topologies of different classes with the corresponding observed frequencies. The correlation test was performed by the cor.test() function in R, with the sample size n = 3003 total possible topologies. (b) The frequencies of the CDS gene

trees and the simulated trees, and the order was according to the observed frequencies from large to small. (c) Only the four species come from different group (Eud: Eudicots, Cde: Ceratophyllaceae, Mag: magnoliids, Mon: monocots and Out: the basal angiosperms and gymnosperms) within (b) were showed. (d) The topology frequency estimated by Twisst software (https://github.com/simonhmartin/twisst.git) of each four group. Eud represent Eudicots. Cde represent Ceratophyllales. Mag represent Magnoliids. Mon represent Monocots. Out represent the basal angiosperms and gymnosperms. The three color represent the three topologies within one four species group. For example, a four species group is A, B, C and D and the different colors meaning: ed represent ((A,B),(C,D)), green represent ((A,C),(B,D)) and blue represent ((A,D),(B,C)).

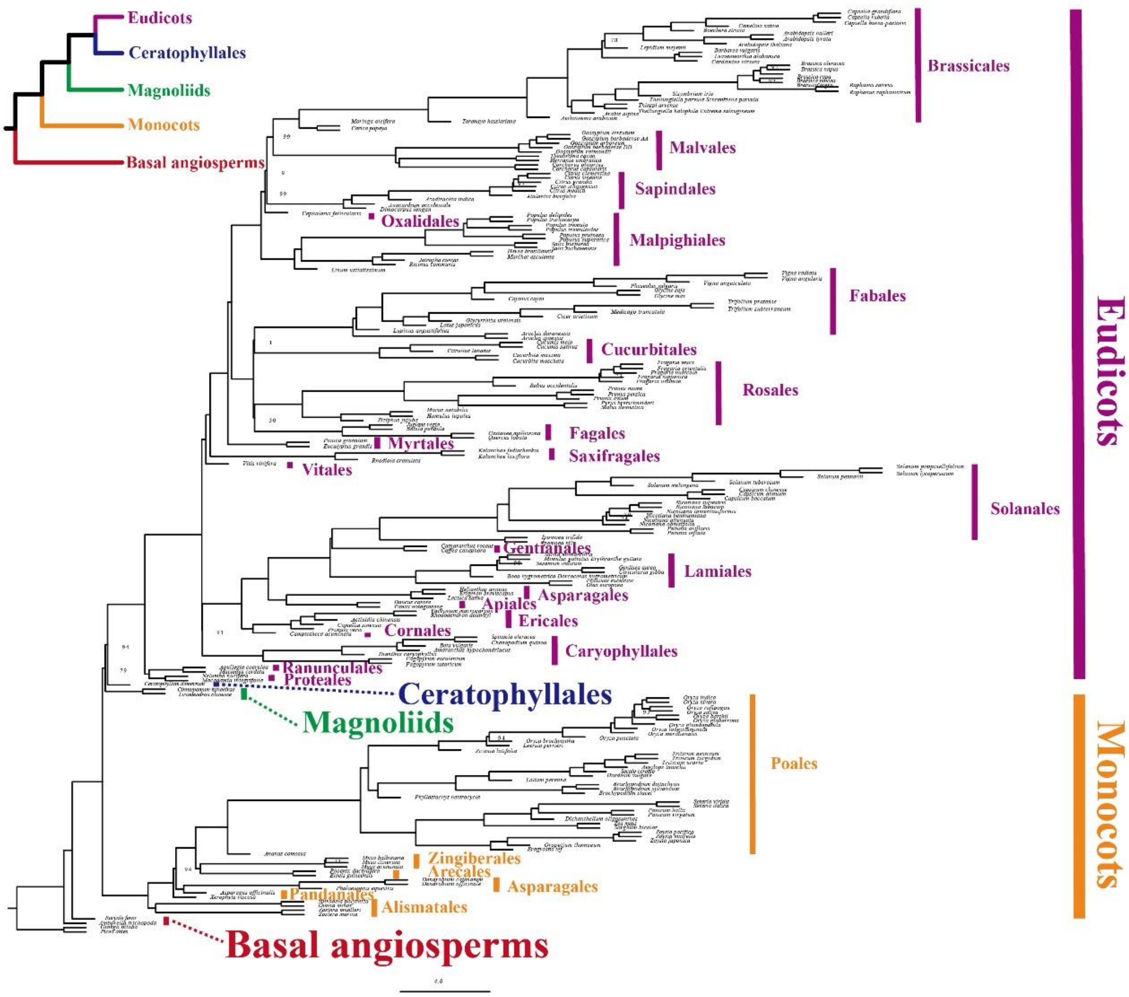

Supplementary Figure 24. Species tree inferred based on the CDS sequences from 612 mostly single-copy OGs by ASTRAL.

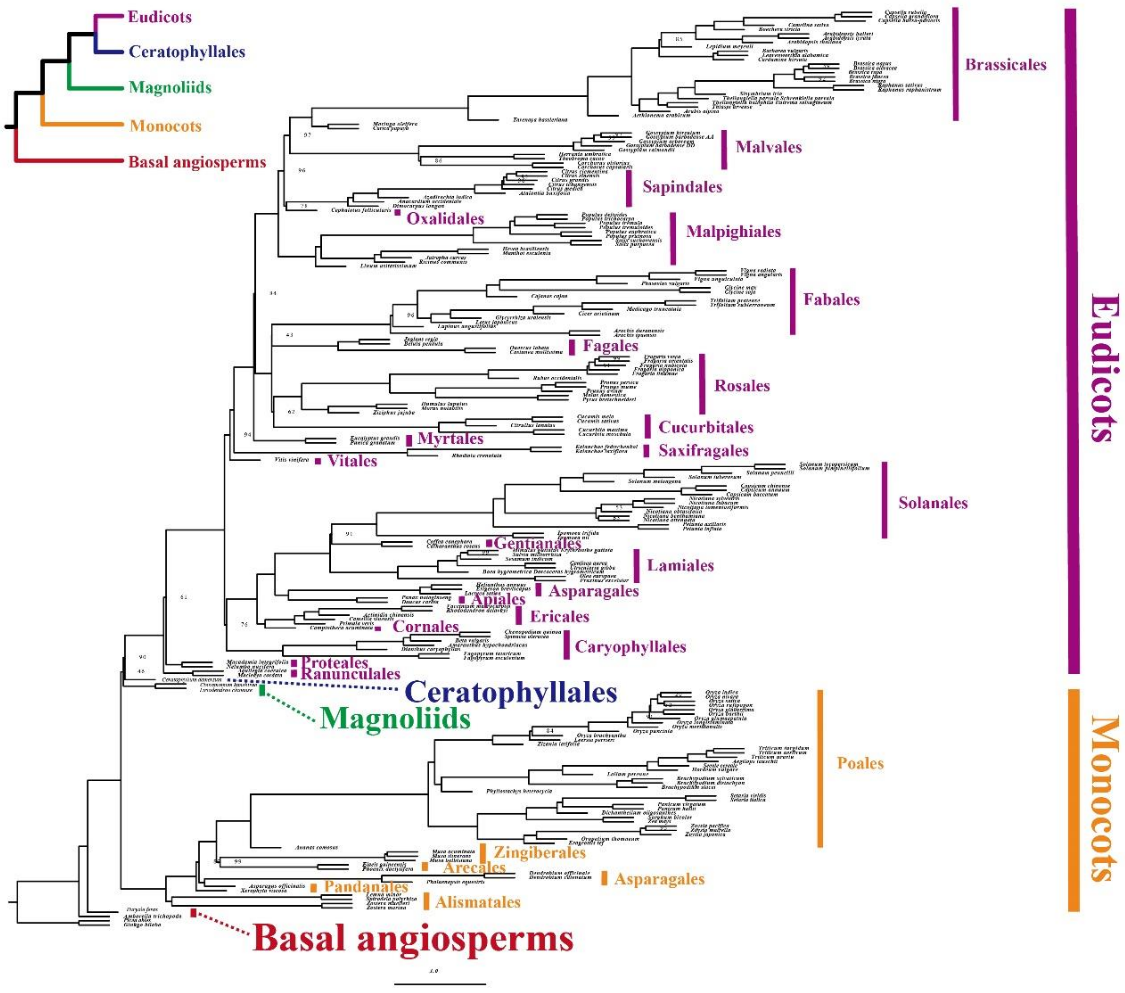

Supplementary Figure 25. Species tree inferred based on the Codon12 (1st and 2nd Codon) sequences from 612 mostly single-copy OGs by ASTRAL.

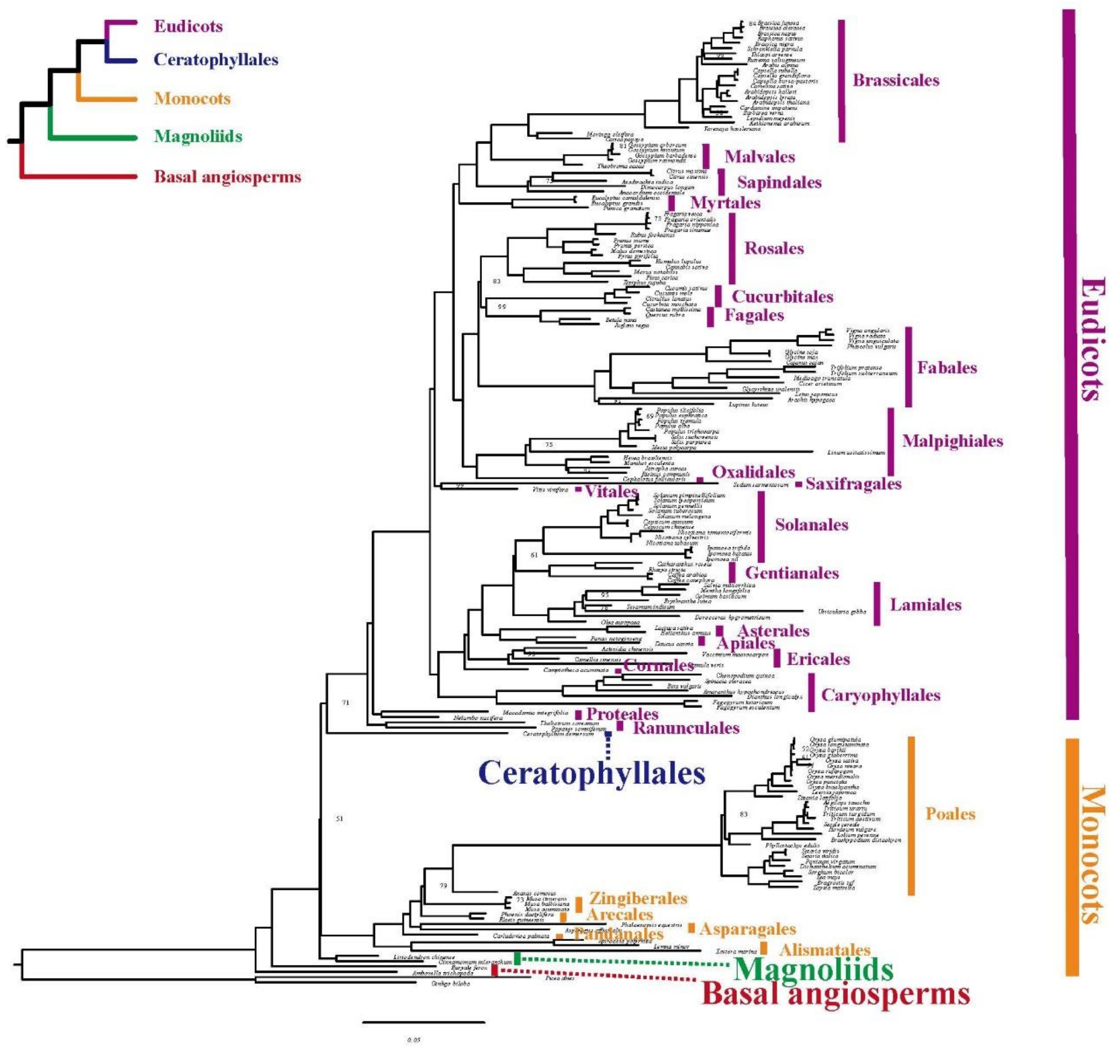

Supplementary Figure 26. The Maximum Likelihood (ML) tree inferred based on the 72 chloroplast gene' coding sequences from 213 species.

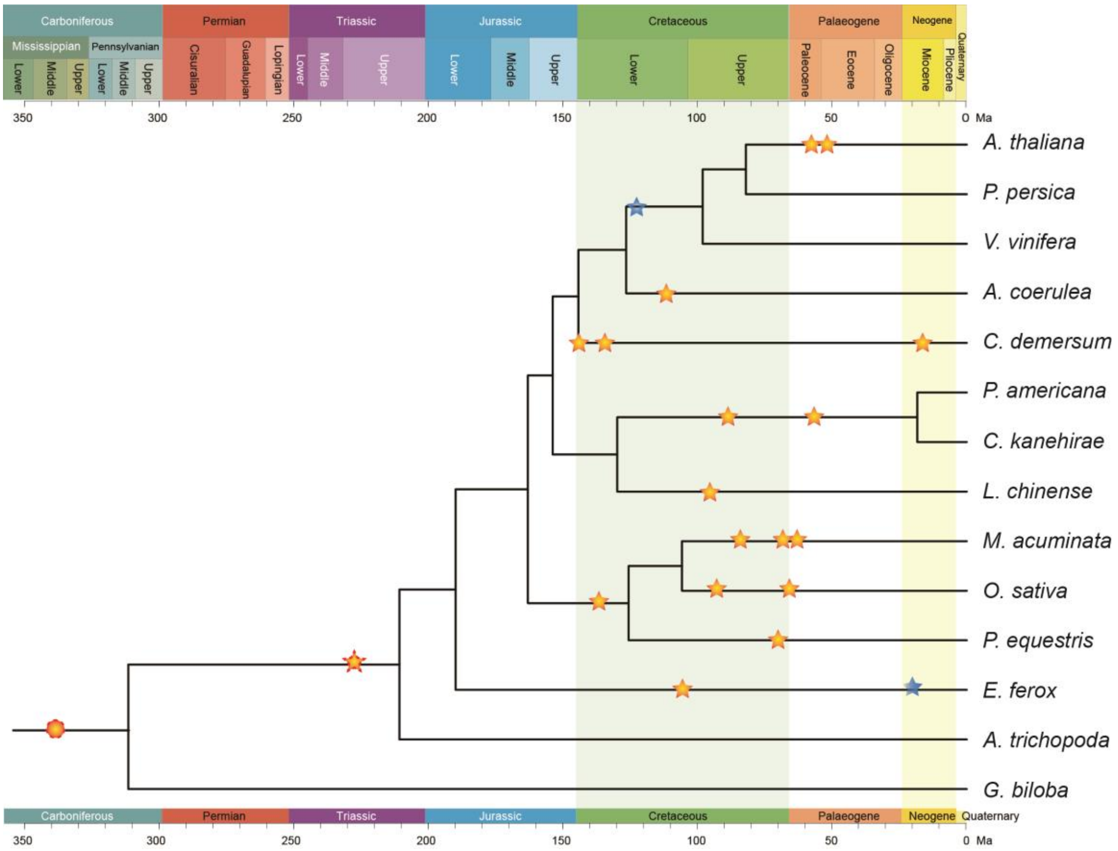

Supplementary Figure 27. Polyploidizations in the evolutionary histories of 14 species. The polyploidizations of four species (*C. demersum*, *L. chinense*, *C. kanehirae*, *E. ferox*) were determined in this paper while the other species were mainly referred from https://doi.org/10.1038/nrg.2017.26. Yellow stars represent the whole genome duplication (WGD), the blue stars represent the whole genome triplication (WGT) and the yellow circle represent the polyploid events is uncertain (WGD, WGT or even higher polyploid). The star or circle with edge marked by the red dash line represent the occurrence time is uncertain of this polyploid event.

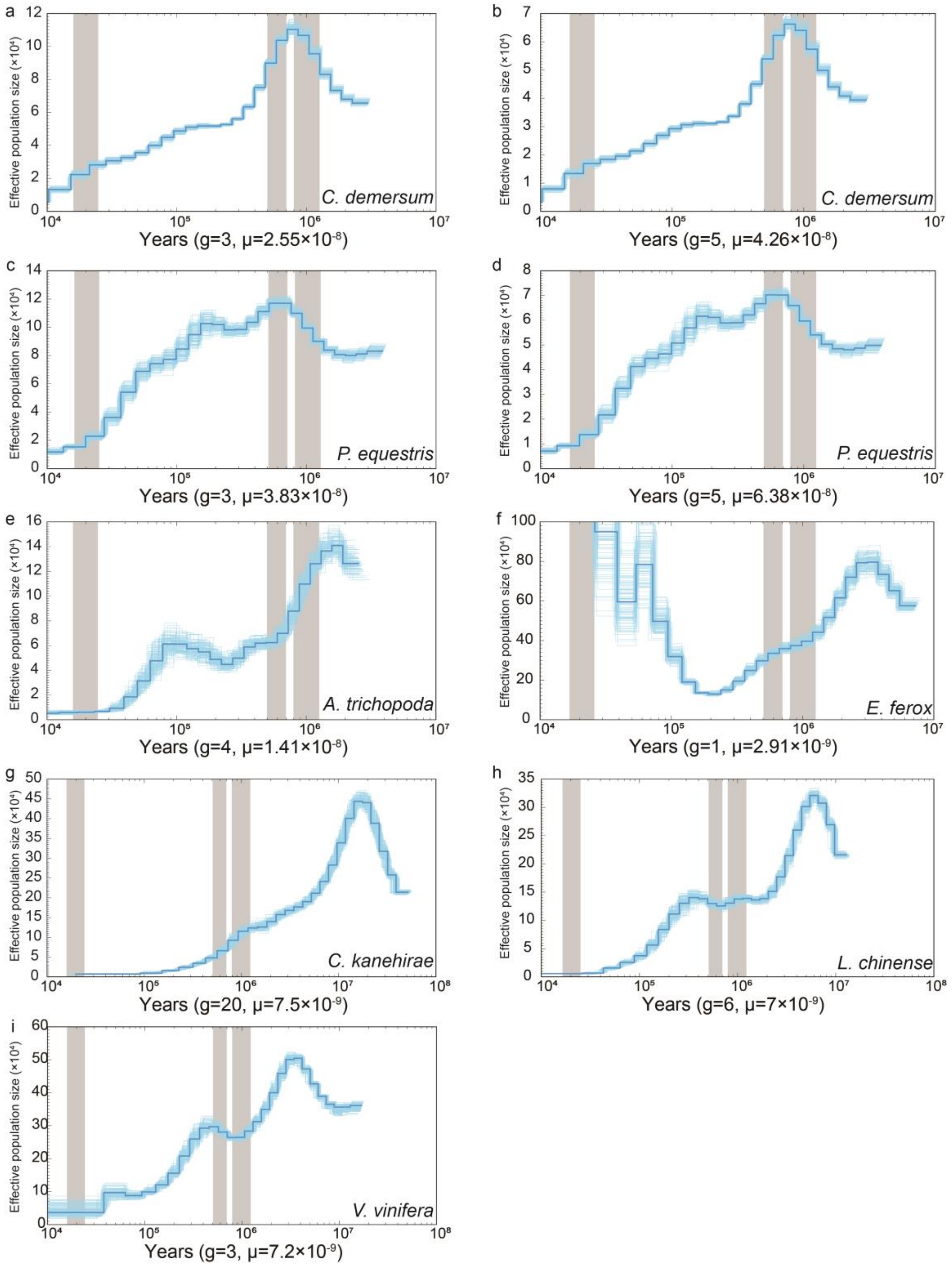

Supplementary Figure 28. Demographic history inferred by PSMC. The period of the Xixiabangma Glaciation (XG, 1,170–800 thousand years ago, kya), Naynayxungla Glaciation (NG, 780–500 kya) and the last glacial maximum (LGM, B20 kya) are shaded in grey. For the species *C. demersum*, *E. ferox* and *P. equestris*, mutation rates (per site per year) were estimated by r8s. We set the generation time of *E. ferox* to 1 year as it was an annual plant (f). While for the other two species we have tried the generation time of 3 and 5 years as they were perennial herbs (a-d). we found when the mutation rates (per site per year) were fixed, the trajectory of population

size changing were same and the generation time only effected the population size at each node. The generation time and mutation rates of the other four species were obtained from their published genome or resequencing paper (e, j-i). The Xixiabangma glaciation (1.17–0.8 Mya), Naynayxungla glaciation (0.78–0.5 Mya) and the last glacial maximum (~20 kya) are shaded in grey. The heterozygosity of *E. ferox* is very low (0.02%) and could be treated as single haploid genome because of the widespread artificial planting this species. We have added one more individual with the heterozygosity of 0.07%, and both the two individuals were used to construct the pseudodiploid genomes for the PSMC' analyses.

Supplementary Tables

Supplementary Table 1. Genome sizes estimated based on 17-mer statistics.

Species	Used Bases	Used Reads	K-mer Number	K-mer Depth	Genome Size (Mb)
Prickly waterlily	16,338,812,400	108,925,416	14,595,239,321	19	768.2
Hornwort	49,214,026,800	328,093,512	43,956,493,200	57	777.2

Supplementary Table 2. The total sequencing data for the two species.

Species	Data type	Platform	Library type	Number of reads	Total Bases (Gb)	Mean read length (bp)	Read N50 length (bp)
**prickly waterlily**	**Genome**	HiSeq2000 (raw)	Paired	324,302,586	48.65	150	-
		HiSeq2000 (clean)[1]	Paired	318,450,322	47.35	148	-
		ONT PromethION (raw)	Single	3,019,400	46.35	15,352	23,412
		ONT PromethION (clean)[1]	Single	1,788,662	31.68	17,710	24,518
		Hi-C (raw)	Paired	562,521,870	84.38	150	-
	**Trancriptome**	RNA-seq	Paired	124,580,456	18.69	150	-
**hornwort**	**Genome**	HiSeq2000 (raw)	Paired	328,093,512	49.21	150	-
		HiSeq2000 (clean)[1]	Paired	315,914,852	46.41	147	-
		ONT PromethION (raw)	Single	4,519,680	106.39	23,539	42,952
		ONT PromethION (clean)[1]	Single	2,371,436	80.51	33,949	50,861
		Hi-C (raw)	Paired	892,591,026	133.89	150	-
	**Trancriptome**	RNA-seq	Paired	38,969,088	5.85	150	-

[1]The clean data means the raw data after quality control. For HiSeq reads we used fastp software with those commands: -q 20 -5 -3; for long reads we just removed the reads with a mean quality score of 7.

Supplementary Table 3. Summary of two genome assemblies.

	Prickly waterlily		Hornwort	
	**Size (Mb)**	**Number**	**Size (Mb)**	**Number**
**N90**	1.41	149	0.25	560
**N50** [1]	4.75	44	1.56	136
**Longest**	17.51	-	9.03	-
**Total Contigs**	725.23	451	733.26	1,366
**Total number (≥10kb)**	-	447	-	1,365
**Total number (≥100kb)**	-	243	-	820
**Contig number (>= 1Mb)**	-	170	-	219
**GC content (%)**	-	36.45	-	31.98

[1]N50 refer to the size above which 50% of the total length of the sequence assembly can be found.

Supplementary Table 4. Summary of two genome chromosome leveled assemblies.

**Prickly waterlily**				**Hornwort**			
**Chromosome**	**Length**	**# of contigs**	**GC content**	**Chromosome**	**Length**	**# of contigs**	**GC content**
**Chr01**	36,127,098	12	36.40%	**Chr01**	131,128,993	154	31.76%
**Chr02**	33,214,131	12	36.35%	**Chr02**	102,323,025	135	31.68%
**Chr03**	32,085,092	13	36.85%	**Chr03**	96,924,528	121	31.72%
**Chr04**	30,354,375	16	36.37%	**Chr04**	81,130,177	119	31.62%
**Chr05**	30,301,737	19	36.62%	**Chr05**	57,145,602	93	31.72%
**Chr06**	29,562,656	19	36.12%	**Chr06**	50,865,708	79	31.55%
**Chr07**	29,178,413	14	37.07%	**Chr07**	40,329,338	65	31.65%
**Chr08**	28,391,438	12	35.97%	**Chr08**	36,680,977	58	31.69%
**Chr09**	26,655,583	15	35.99%	**Chr09**	33,314,896	58	31.67%
**Chr10**	26,604,565	16	36.03%	**Chr10**	31,803,598	73	31.76%
**Chr11**	25,703,169	13	36.39%	**Chr11**	25,714,425	48	31.67%
**Chr12**	25,325,963	17	36.20%	**Chr12**	16,430,251	35	31.49%
**Chr13**	24,897,859	10	36.97%	**Total**	703,791,518	1,038	31.68%
**Chr14**	24,782,706	19	36.69%				
**Chr15**	23,866,150	21	35.91%				
**Chr16**	23,493,034	17	36.54%				
**Chr17**	23,053,665	14	36.18%				
**Chr18**	22,747,766	9	35.97%				
**Chr19**	22,494,454	12	35.69%				
**Chr20**	22,322,537	9	36.97%				
**Chr21**	21,789,828	12	36.54%				
**Chr22**	21,469,475	11	36.77%				
**Chr23**	21,357,665	5	36.12%				
**Chr24**	20,198,942	7	36.45%				
**Chr25**	20,182,140	13	36.85%				
**Chr26**	19,815,768	2	36.96%				
**Chr27**	19,323,887	6	37.02%				
**Chr28**	18,973,550	5	36.54%				
**Chr29**	16,955,657	4	36.63%				
**Total**	721,229,303	35,400	36.44%				

Supplementary Table 5. Assessment of the two species genome assembly by BUSCO.

Description	Number of genes		% Percentage	
	Prickly waterlily	Hornwort	Prickly waterlily	Hornwort
Complete BUSCOs	1,233	1,196	85.7	83.1
Complete and single-copy BUSCOs	764	990	53.1	68.8
Complete and duplicated BUSCOs	469	206	32.6	14.3
Fragmented BUSCOs	40	51	2.8	3.5
Missing BUSCOs	147	193	11.5	13.4

Supplementary Table 6. Numbers of NGS reads mapped to the assembles.

	Total pairs (PE) reads	Pair end mapped reads	Pair end mapped ratio (%)	Single end mapped reads	Single end mapped ratio (%)	Total mapped reads	Total mapped ratio (%)
**Prickly waterlily**	318,853,617	308,313,098	96.69	162,799	0.05	308,879,192	96.87
**Hornwort**	323,088,340	266,431,704	82.46	1,377,948	0.42	274,983,140	85.11

Supplementary Table 7. Evaluation of completeness of the two genomes using data set of *de novo* assembled transcripts.

Data set	Length type	Number	Total length	Covered by assembly (%)	With >90% sequence in one scaffold		With >50% sequence in on scaffold	
					Number	Percentage (%)	Number	Percentage (%)
**Prickly waterlily**	>200 bp	161,944	148,574,288	94.23	143,101	88.36	151,952	93.83
	>500 bp	78,535	122,443,098	98.96	72,491	92.30	77,508	98.69
	>1 kb	45,133	99,023,091	99.69	42,108	93.30	44,939	99.57
**Hornwort**	>200 bp	186,160	88,551,535	86.08	150,614	80.91	158,678	85.24
	>500 bp	54,962	48,991,430	97.37	50,749	92.33	53,216	96.82
	>1 kb	15,359	21,561,387	99.18	14,425	93.92	15,151	98.65

Supplementary Table 8. Comparison of gene space of the prickly waterlily and hornwort genomes with other genomes.

Species	Total Genes	Average Gene Length[a] (bp)	Average CDS Length[a] (bp)	Average Exons per Gene[a]	Average Exon Length[a] (bp)	Average Intron Length[a] (bp)
A. trichopoda	27,313	5,607.1	944.9	4.1	232.7	1,523.5
E. ferox	40,297	6,174.7	1,231.6	5.7	217.7	938.4
M. acuminata	36,525	3,804.1	1,038.4	5.4	191.9	579.4
O. sativa	35,825	3,087.0	986.1	3.8	262.8	429.4
P. equestris	29,431	9,528.9	1,096.0	3.9	278.7	2,908.4
C. demersum	30,138	10,643.0	1,239.2	5.2	239.8	2,171.0
C. kanehirae	27,885	7,596.1	1,311.0	5.4	242.6	1,427.0
L. chinense	35,535	11,430.2	1,266.9	4.9	260.2	2,395.5
A. coerulea	30,023	3,577.2	1,122.7	4.6	244.1	468.3
V. vinifera	29,971	5,133.6	1,095.8	4.7	230.7	968.9
A. thaliana	27,655	2,368.3	1,201.6	5.1	237.5	159.5
P. persica	26,873	3,237.5	1,215.2	4.9	248.0	332.3

Supplementary Table 9. Summary of gene family clustering among the 14 genomes (including 12 angiosperms and 2 gymnosperms).

Species	Total genes	Genes in families	Unclustered genes	Families	Unique families	Genes per family	Maximum gene family size
A. coerulea	29,702	23,890	5,812	12,985	941	1.84	194
A. thaliana	27,307	23,394	3,913	12,247	816	1.91	138
A. trichopoda	26,814	19,132	7,682	12,243	958	1.56	178
C. demersum	29,717	21,160	8,557	11,083	1,016	1.91	30
C. kanehirae	27,853	23,506	4,347	12,124	522	1.94	131
E. ferox	40,197	31,481	8,716	11,745	1,139	2.68	85
G. biloba	27,310	11,426	15,884	9,205	246	1.24	13
L. chinense	35,264	31,294	3,970	12,291	756	2.55	793
M. acuminata	34,318	25,879	8,439	11,954	595	2.16	73
O. sativa	34,412	22,964	11,448	12,540	1,471	1.83	65
P. abies	22,159	18,003	4,156	8,539	1,017	2.11	498
P. equestris	28,892	20,392	8,500	11,789	878	1.73	434
P. persica	26,783	22,243	4,540	12,976	516	1.71	139
V. vinifera	28,284	22,203	6,081	12,918	658	1.72	118
**All**	419,012	316,967	102,045	28,710	-	11.04	-

Supplementary Table 10. Functional annotation of the predicted genes.

	Database	Prickly waterlily		Hornwort	
		**Number**	**Percent (%)**	**Number**	**Percent (%)**
**Total**		40,297	100	30,138	100
	**InterPro**	30,281	75.14	23,265	77.19
	**GO**	21,905	54.36	16,591	55.05
**Annotated**	**Swissprot**	28,216	70.02	21,484	71.29
	**TrEMBL**	34,131	84.70	26,835	89.04
	**KEGG**	17,169	42.60	13,425	44.55
**Unannotated**		5,793	14.38	3,081	10.22

Supplementary Table 11. Prediction of repetitive elements in the assembled prickly waterlily and hornwort genomes.

Type	Prickly waterlily		Hornwort	
	length (bp)	percentage of genome (%)	length (bp)	percentage of genome (%)
**Tandem repeats**	94,553	1.05	5,080,212	0.69
**satellites**	94,553	0.01	3,572,447	0.49
**simple repeat**	7,518,091	1.04	1,507,765	0.21
**Interspersed repeats**	270,402,304	37.29	457,483,862	62.39
**SINEs**	167,387	0.02	1,402,370	0.19
**LINEs**	551,798	0.08	159,078,812	21.69
**LTR elements**	118,221,353	16.30	145,252,899	19.81
Gypsy	105,831,755	14.59	96,912,734	13.22
Copia	12,058,578	1.66	18,839,836	2.57
**DNA transposons**	33,259,656	4.59	44,049,067	6.01
**unclassified**	118,202,110	16.30	107,700,714	14.69
**In total**	278,114,948	38.35	462,564,074	63.08

Supplementary Table 12. Kernel function analysis of Ks distribution related to duplication events within each genome and between genomes (after evolutionary rate correction).

Intragenomic/Intergenomic colinear gene pairs	Weight coefficient related to duplication event or speciation	Peak of Ks distribution (μ)	Deviation (σ)	Number of syntenic blocks
Vitis_Vitis	0.9544	1.0305	0.1104	143
Liriodendron-Liriodendron	0.8827	0.8070	0.0936	161
Euryale-Euryale-1[a]	0.5353	0.1420	0.0239	603
Euryale-Euryale-2[a]	0.4520	0.8407	0.0780	1,365
Ceratophyllum-Ceratophyllum-1[a]	0.4182	0.1162	0.0209	162
Ceratophyllum-Ceratophyllum-2[a]	0.3837	1.1362	0.1321	79
Ceratophyllum-Ceratophyllum-3[a]	0.1048	1.4061	0.0488	60
Cinnamomum-Cinnamomum-1[a]	0.3841	0.9443	0.0954	79
Cinnamomum-Cinnamomum-2[a]	0.1266	0.5956	0.0493	195
Avocado-Avocado-1[a]	0.3328	0.5956	0.0813	41
Avocado-Avocado-2[a]	0.2878	0.9443	0.1114	30
Amborella-Euryale	0.9483	1.6342	0.2149	427
Amborella-Oryza	0.9597	1.6392	0.2221	151
Amborella-Vitis	0.9526	1.6341	0.2022	259
Ceratophyllum-Oryza	0.8573	1.5014	0.2580	172
Cinnamomum-Oryza	0.8348	1.4702	0.1533	376
Ceratophyllum-Vitis	0.9525	1.4138	0.1802	401
Ceratophyllum-Cinnamomum	0.9186	1.4189	0.1634	518
Liriodendron-Vitis	0.9558	1.2768	0.1269	380
Cinnamomum-Vitis	0.9483	1.2720	0.1305	480
Liriodendron-Cinnamomum	0.9720	0.9453	0.1297	409
Cinnamomum-Avocado	0.3223	0.0801	0.0509	105

[a]Representing the whole genome polyploidization events occurred orders (from the old to recent).

Supplementary Table 13. Data on all 15 species used during the evolution analysis.

Species	Database	Number of genes	Number of original genes
G. biloba	PLAZA v4.0	27,310	30,404
P. abies	PLAZA v4.0	22,159	26,437
A. trichopoda	Ensembl Plants v42	26,814	27,313
E. ferox	-	40,197	40,297
M. acuminata	Ensembl Plants v42	34,318	36,525
O. sativa	Ensembl Plants v42	34,412	35,825
P. equestris	PLAZA v4.0	29,406	29,431
C. demersum	-	29,717	30,138
C. kanehirae	NCBI (ASBRC_Ckan_1.0)	27,853	27,885
L. chinense	NCBI (NJFU_Lchi_2.0)	35,264	35,269
A. coerulea	JGI v12	29,702	30,023
V. vinifera	Ensembl Plants v42	28,284	29,927
A. thaliana	Ensembl Plants v42	27,307	27,678
P. persica	Ensembl Plants v42	26,783	26,873
P. americana	CoGe	22,441	22,270

Supplementary Table 14. The gene length information of the 72 plastid genes among the 15 species.

Geneid	*A. coerulea*	*A. thaliana*	*A. trichopoda*	*C. demersum*	*C. kanehirae*	*E. ferox*	*G. biloba*	*L. chinense*	*M. acuminata*	*O. sativa*	*P. abies*	*P. americana*	*P. equestris*	*P. persica*	*V. vinifera*
accD	1494	1467	1623	1527	1512	1461	972	1467	1800	-	981	1512	1488	1476	1527
atpA	1524	1524	1524	1524	1524	1524	1533	1524	1524	1524	1524	1524	1524	1524	1524
atpB	1497	1497	1503	1497	1497	1497	1464	1497	1497	-	1479	1497	1497	1479	1497
atpE	402	399	405	402	405	405	417	405	408	414	414	405	405	402	402
atpF	555	555	555	555	555	555	570	555	555	-	555	555	555	555	558
atpH	246	246	246	246	246	246	246	246	246	246	246	246	246	246	246
atpI	744	750	747	744	744	747	747	744	744	744	747	744	744	744	744
ccsA	984	987	942	960	-	927	1020	966	990	966	963	951	966	969	969
cemA	681	690	690	690	690	690	705	690	690	-	786	690	690	690	690
clpP	-	591	609	609	609	609	600	606	621	651	591	609	612	588	591
matK	1530	1515	1506	1548	1524	1524	1539	1524	1536	1536	1548	1524	1341	1521	1509
ndhC	363	363	363	363	363	363	408	363	363	363	-	363	-	363	363
ndhD	1512	1503	1503	1503	1509	1503	1506	1500	1506	1503	-	1506	-	1503	1503
ndhE	306	306	306	306	306	306	303	306	306	306	-	306	-	306	306
ndhG	534	531	534	531	531	534	543	531	531	531	-	531	-	531	531
ndhH	1182	1182	1182	1182	1182	1182	1176	1182	1182	1182	-	1182	-	1182	1182
ndhI	540	519	543	513	543	540	543	543	543	543	-	543	-	519	504
ndhJ	477	477	477	477	477	477	477	477	480	480	-	477	-	477	477
ndhK	684	678	678	750	858	678	957	858	753	678	-	858	-	681	678
petA	969	963	963	963	963	969	963	963	963	963	960	963	963	963	963
petB	648	648	648	648	648	648	657	648	654	-	648	648	648	648	648
petD	528	483	483	483	477	513	474	489	483	483	537	489	492	483	483
petG	114	114	114	114	114	114	114	114	114	-	114	114	114	114	114
petL	96	96	-	96	96	96	108	96	96	-	102	96	96	96	96
petN	90	90	90	90	90	90	90	90	90	-	90	90	90	90	90

psaA	2253	2253	2253	2253	2253	2253	2253	2253	2253	2253	2253	2253	-	2253	2253
psaB	2205	2205	2205	2205	2205	2205	2205	2205	2205	2205	2205	2205	-	2205	2205
psaC	246	246	246	246	246	246	246	246	246	246	246	246	-	246	246
psaI	111	114	-	111	111	111	111	111	111	-	111	111	111	114	111
psaJ	135	135	129	135	135	135	135	135	129	-	129	135	135	135	132
psbA	1062	1062	1053	1062	1062	1062	1062	1062	1062	1062	1062	1062	1062	1062	1062
psbB	1527	1527	1527	1527	1527	1527	1527	1527	1527	1527	1527	1527	1527	1527	1527
psbC	1422	1422	1422	1386	1422	1422	1380	1422	1422	1422	1422	1422	1422	1422	1422
psbD	1062	1062	1062	1062	1062	1062	1008	1062	1062	1062	1062	1062	1062	1062	1062
psbE	252	252	252	252	252	252	252	252	252	252	252	252	252	252	252
psbF	120	120	120	120	120	120	120	120	120	120	120	120	120	120	120
psbH	222	222	222	222	222	222	228	222	222	231	228	222	222	225	240
psbI	111	111	111	111	162	111	162	111	111	111	111	111	111	111	111
psbJ	123	123	123	123	123	123	123	123	123	-	123	123	123	123	123
psbK	186	186	186	180	180	186	177	186	186	186	180	180	186	180	186
psbL	117	117	117	117	117	117	117	117	117	-	117	117	117	117	117
psbM	105	105	-	105	105	105	105	105	105	207	114	105	105	105	105
psbN	132	132	132	132	132	132	132	132	132	294	132	132	132	132	132
psbT	102	102	108	108	117	117	108	108	102	-	108	108	108	108	108
rbcL	1428	1440	1428	1428	1428	1428	1428	1431	1464	1455	1428	1428	1464	1428	1428
rpl14	369	369	369	369	369	369	369	369	369	372	369	369	369	369	369
rpl16	459	408	408	408	408	408	417	408	411	414	405	405	408	408	408
rpl2	825	825	830	822	822	822	828	819	816	315	831	822	822	819	825
rpl20	354	354	360	354	354	348	348	354	354	-	360	354	354	354	354
rpl22	546	483	375	429	-	378	393	-	390	450	429	-	360	426	489
rpl23	282	282	288	282	-	282	81	282	282	282	276	291	282	282	282
rpl32	-	159	174	174	174	159	210	174	174	180	213	174	174	159	174
rpl33	207	201	207	201	201	207	201	201	201	201	207	201	201	201	201
rpl36	114	114	114	114	114	114	114	114	114	-	114	114	114	114	114
rpoA	1050	990	1005	1014	1020	1026	1023	1014	1020	1014	1011	1020	1014	981	996

rpoB	3213	3219	3219	3213	3234	3216	3219	3213	3225	3228	3231	3183	3276	3213	3213
rpoC1	2043	2043	2043	2043	2052	2076	2055	2046	2061	2049	2091	2052	2043	2052	2049
rpoC2	4146	4131	4110	4173	4158	4197	4110	4149	4095	4542	3657	4167	4167	4107	4185
rps11	417	417	417	417	393	417	393	417	417	432	393	393	417	417	417
rps12	372	372	372	381	357	372	369	372	369	-	372	372	372	372	372
rps14	303	303	303	303	303	303	303	303	303	312	300	303	303	303	303
rps15	264	267	264	276	267	264	267	273	273	273	267	267	276	273	267
rps16	261	240	237	261	270	228	231	261	252	-	-	270	285	270	276
rps18	345	306	306	306	306	306	252	306	306	492	282	306	306	303	306
rps19	279	279	279	279	279	279	279	279	279	282	279	279	279	279	279
rps2	711	711	711	711	711	711	699	711	711	711	705	711	711	711	711
rps3	669	657	657	657	663	657	657	660	657	720	657	663	657	657	657
rps4	600	606	606	606	606	606	612	606	606	606	606	606	606	606	606
rps7	468	468	468	468	468	468	468	468	468	471	468	468	468	468	468
rps8	405	405	399	399	399	399	417	399	399	411	399	399	396	405	405
ycf3	507	507	507	507	507	507	513	507	507	-	513	507	507	507	507
ycf4	555	555	708	555	555	555	555	555	555	558	555	555	555	555	561

Supplementary Table 15. Data on the previous sequenced 211 species used during the phylogenetic tree analysis.

Order	Family	Species	Database	Version	Genbank Accession
Alismatales	Araceae	*Spirodela polyrhiza*	NCBI	ASM198140v1	GCA_001981405.1
Alismatales	Lemnaceae	*Lemna minor*	CoGe database	id 27408	-
Alismatales	Zosteraceae	*Zostera marina*	NCBI	Zosma_marina.v.2.1	GCA_001185155.1
Alismatales	Zosteraceae	*Zostera muelleri*	Seagrass Zmu Genome (http://appliedbioinformatics.com.au/index.php/Seagrass_Zmu_Genome)	Zostera muelleri	-
Amborellales	Amborellaceae	*Amborella trichopoda*	NCBI	AMTR1.0	GCA_000471905.1
Apiales	Apiaceae	*Daucus carota*	NCBI	ASM162521v1	GCA_001625215.1
Apiales	Araliaceae	*Panax notoginseng*	Panax notoginseng (http://www.plantkingdomgdb.com/panax_notoginseng/data/)	Panax notoginseng	-
Arecales	Arecaceae	*Elaeis guineensis*	NCBI	EG5	GCA_000442705.1
Arecales	Arecaceae	*Phoenix dactylifera*	NCBI	DPV01	GCA_000413155.1
Asparagales	Asparagaceae	*Asparagus officinalis*	NCBI	Aspof.V1	GCA_001876935.1
Asparagales	Orchidaceae	*Dendrobium catenatum*	NCBI	ASM160598v2	GCA_001605985.2
Asparagales	Orchidaceae	*Dendrobium officinale*	Herbal Medicine Omics Database (ftp://202.203.187.112:2222/genome/dendrobe/)	Dendrobium officinale	-
Asparagales	Orchidaceae	*Phalaenopsis equestris*	NCBI	ASM126359v1	GCA_001263595.1
Asterales	Asteraceae	*Erigeron breviscapus*	GigaDB (http://dx.doi.org/10.5524/100290)	Erigeron breviscapus	-
Asterales	Asteraceae	*Helianthus annuus*	NCBI	HanXRQr1.0	GCA_002127325.1
Asterales	Asteraceae	*Lactuca sativa*	NCBI	Lsat_Salinas_v7	GCA_002870075.1
Brassicales	Brassicaceae	*Aethionema arabicum*	NCBI	VEGI_AA_v_1.0	GCA_000411095.1
Brassicales	Brassicaceae	*Arabidopsis halleri*	NCBI	Ahal_1.0	GCA_000523005.1
Brassicales	Brassicaceae	*Arabidopsis lyrata*	NCBI	v.1.0	GCA_000004255.1
Brassicales	Brassicaceae	*Arabidopsis thaliana*	NCBI	TAIR10.1	GCA_000001735.2
Brassicales	Brassicaceae	*Arabis alpina*	NCBI	MPIPZ.v5	GCA_900128785.1
Brassicales	Brassicaceae	*Barbarea vulgaris*	NCBI	ASM192098v1	GCA_001920985.1
Brassicales	Brassicaceae	*Boechera stricta*	NCBI	Bstricta_278_v1	GCA_002079875.1
Brassicales	Brassicaceae	*Brassica juncea*	NCBI	Brassica_juncea_var_tumida_T84-66_v1	GCA_001687265.1
Brassicales	Brassicaceae	*Brassica napus*	NCBI	Bra_napus_v2.0	GCA_000686985.2
Brassicales	Brassicaceae	*Brassica nigra*	NCBI	ASM168289v1	GCA_001682895.1
Brassicales	Brassicaceae	*Brassica oleracea*	NCBI	BOL	GCA_000695525.1
Brassicales	Brassicaceae	*Brassica rapa*	NCBI	ASM30998v2	GCA_000309985.2
Brassicales	Brassicaceae	*Camelina sativa*	NCBI	Cs	GCA_000633955.1
Brassicales	Brassicaceae	*Capsella bursa-pastoris*	NCBI	C_bursa_pastoris_nuclear_genome_v1	GCA_001974645.1

Brassicales	Brassicaceae	*Capsella grandiflora*	JGI12	V1.1	-
Brassicales	Brassicaceae	*Capsella rubella*	NCBI	Caprub1_0	GCA_000375325.1
Brassicales	Brassicaceae	*Cardamine hirsuta*	Cardamine hirsuta genetic and genomic (http://chi.mpipz.mpg.de/assembly.html)	v1.0	-
Brassicales	Brassicaceae	*Leavenworthia alabamica*	NCBI	VEGI_LA_v_1.0	GCA_000411055.1
Brassicales	Brassicaceae	*Lepidium meyenii*	Maca Genome Hub	V1.0	-
Brassicales	Brassicaceae	*Raphanus raphanistrum*	NCBI	ASM76984v1	GCA_000769845.1
Brassicales	Brassicaceae	*Raphanus sativus*	NCBI	Rs1.0	GCA_000801105.2
Brassicales	Brassicaceae	*Sisymbrium irio*	NCBI	VEGI_SI_v_1.0	GCA_000411075.1
Brassicales	Brassicaceae	*Thellungiella halophila | Eutrema salsugineum*	NCBI	Eutsalg1_0	GCA_000478725.1
Brassicales	Brassicaceae	*Thellungiella parvula | Schrenkiella parvula*	NCBI	Eutrema_parvulum_v01	GCA_000218505.1
Brassicales	Brassicaceae	*Thlaspi arvense*	NCBI	T_arvense_v1	GCA_000956625.1
Brassicales	Caricaceae	*Carica papaya*	NCBI	ASM131004v1	GCA_001310045.1
Brassicales	Cleomaceae	*Tarenaya hassleriana*	NCBI	ASM46358v1	GCA_000463585.1
Brassicales	Moringaceae	*Moringa oleifera*	Herbal Medicine Omics Database (ftp://202.203.187.112:2222/genome/dendrobe/)	Moringa oleifera	-
Caryophyllales	Amaranthaceae	*Amaranthus hypochondriacus*	NCBI	AHP 1.0	GCA_000753965.1
Caryophyllales	Amaranthaceae	*Beta vulgaris*	Ensembl Plants v42	RefBeet-1.2.2	GCA_000511025.2
Caryophyllales	Amaranthaceae	*Chenopodium quinoa*	NCBI	ASM168347v1	GCA_001683475.1
Caryophyllales	Amaranthaceae	*Spinacia oleracea*	NCBI	ASM200726v1	GCA_002007265.1
Caryophyllales	Caryophyllaceae	*Dianthus caryophyllus*	NCBI	DCA_r1.0	GCA_000512335.1
Caryophyllales	Polygonaceae	*Fagopyrum esculentum*	NCBI	FES_r1.0	GCA_001661195.1
Caryophyllales	Polygonaceae	*Fagopyrum tataricum*	NCBI	Ft1.0	GCA_002319775.1
Cornales	Nyssaceae	*Camptotheca acuminata*	Medicinal Plant Genomics Resource (http://medicinalplantgenomics.msu.edu)	Camptotheca acuminata	-
Cucurbitales	Cucurbitaceae	*Citrullus lanatus*	NCBI	CiLa_1.0	GCA_000238415.1
Cucurbitales	Cucurbitaceae	*Cucumis melo*	NCBI	ASM31304v1	GCA_000313045.1
Cucurbitales	Cucurbitaceae	*Cucumis sativus*	NCBI	ASM407v2	GCA_000004075.2
Cucurbitales	Cucurbitaceae	*Cucurbita maxima*	NCBI	Cmax_1.0	GCA_002738345.1
Cucurbitales	Cucurbitaceae	*Cucurbita moschata*	NCBI	Cmos_1.0	GCA_002738365.1
Ericales	Actinidiaceae	*Actinidia chinensis*	NCBI	Kiwifruit_v1	GCA_000467755.1
Ericales	Ericaceae	*Rhododendron delavayi*	GigaDB (http://dx.doi.org/10.5524/100331)	Rhododendron delavayi	-

Ericales	Ericaceae	*Vaccinium macrocarpon*	NCBI	ASM77533v2	GCA_000775335.2
Ericales	Primulaceae	*Primula veris*	NCBI	ASM78844v1	GCA_000788445.1
Ericales	Theaceae	*Camellia sinensis*	NCBI	AHAU_CSS_2	GCA_004153795.2
Fabales	Fabaceae	*Arachis duranensis*	NCBI	Aradu1.1	GCA_000817695.2
Fabales	Fabaceae	*Arachis ipaensis*	NCBI	Araip1.1	GCA_000816755.2
Fabales	Fabaceae	*Cajanus cajan*	NCBI	C.cajan_V1.0	GCA_000340665.1
Fabales	Fabaceae	*Cicer arietinum*	NCBI	ASM33114v1	GCA_000331145.1
Fabales	Fabaceae	*Glycine max*	NCBI	Glycine_max_v2.1	GCA_000004515.4
Fabales	Fabaceae	*Glycine soja*	NCBI	glyso.PI483463.gnm1	GCA_002907465.1
Fabales	Fabaceae	*Glycyrrhiza uralensis*	Glycyrrhiza uralensis GDB (http://ngs-data-archive.psc.riken.jp/Gur-genome/)	v1.0	-
Fabales	Fabaceae	*Lotus japonicus*	NCBI	Lj3.0	GCA_000181115.2
Fabales	Fabaceae	*Lupinus angustifolius*	NCBI	LupAngTanjil_v1.0	GCA_001865875.1
Fabales	Fabaceae	*Medicago truncatula*	NCBI	MedtrA17_4.0	GCA_000219495.2
Fabales	Fabaceae	*Phaseolus vulgaris*	NCBI	PhaVulg1_0	GCA_000499845.1
Fabales	Fabaceae	*Trifolium pratense*	NCBI	Trifolium pratense genome v3	GCA_900292005.1
Fabales	Fabaceae	*Trifolium subterraneum*	NCBI	TSUd_r1.1	GCA_001742945.1
Fabales	Fabaceae	*Vigna angularis*	NCBI	Vigan1.1	GCA_001190045.1
Fabales	Fabaceae	*Vigna radiata*	NCBI	Vradiata_ver6	GCA_000741045.2
Fabales	Fabaceae	*Vigna unguiculata*	NCBI	ASM411807v1	GCA_004118075.1
Fagales	Betulaceae	*Betula pendula*	NCBI	Bpev01	GCA_900184695.1
Fagales	Fagaceae	*Castanea mollissima*	NCBI	ASM76360v1	GCA_000763605.1
Fagales	Fagaceae	*Quercus lobata*	NCBI	ValleyOak3.0	GCA_001633185.2
Fagales	Juglandaceae	*Juglans regia*	NCBI	wgs.5d	GCA_001411555.1
Gentianales	Apocynaceae	*Catharanthus roseus*	NCBI	ASM94934v1	GCA_000949345.1
Gentianales	Rubiaceae	*Coffea canephora*	NCBI	AUK_PRJEB4211_v1	GCA_900059795.1
Ginkgoales	Ginkgoaceae	*Ginkgo biloba*	PLAZA v4.0	-	-
Lamiales	Gesneriaceae	*Dorcoceras hygrometricum*	NCBI	Boea_hygrometrica.v1	GCA_001598015.1
Lamiales	Lamiaceae	*Salvia miltiorrhiza*	National Data Center of Traditional Chinese Medicine of China (http://www.ndctcm.org/shujukujieshao/)	Salvia miltiorrhiza	-
Lamiales	Lentibulariaceae	*Genlisea aurea*	NCBI	GenAur_1.0	GCA_000441915.1
Lamiales	Lentibulariaceae	*Utricularia gibba*	NCBI	U_gibba_v2	GCA_002189035.1
Lamiales	Oleaceae	*Fraxinus excelsior*	NCBI	BATG-0.5	GCA_900149125.1
Lamiales	Oleaceae	*Olea europaea*	NCBI	O_europaea_v1	GCA_002742605.1
Lamiales	Pedaliaceae	*Sesamum indicum*	NCBI	S_indicum_v1.0	GCA_000512975.1
Lamiales	Phrymaceae	*Mimulus guttatus* | *Erythranthe guttata*	NCBI	Mimgu1_0	GCA_000504015.1

Laurales	Lauraceae	*Cinnamomum kanehirae*	NCBI	ASBRC_Ckan_1.0	GCA_003546025.1
magnoliales	magnoliaceae	*Liriodendron chinense*	NCBI	NJFU_Lchi_2.0	GCA_003013855.2
Malpighiales	Euphorbiaceae	*Hevea brasiliensis*	NCBI	ASM165405v1	GCA_001654055.1
Malpighiales	Euphorbiaceae	*Jatropha curcas*	NCBI	JatCur_1.0	GCA_000696525.1
Malpighiales	Euphorbiaceae	*Manihot esculenta*	NCBI	Manihot esculenta v6	GCA_001659605.1
Malpighiales	Euphorbiaceae	*Ricinus communis*	NCBI	JCVI_RCG_1.1	GCA_000151685.2
Malpighiales	Linaceae	*Linum usitatissimum*	NCBI	ASM22429v2	GCA_000224295.2
Malpighiales	Salicaceae	*Populus deltoides*	JGI12	Populus deltoides WV94 v2.1	-
Malpighiales	Salicaceae	*Populus euphratica*	NCBI	PopEup_1.0	NC_024747.1
Malpighiales	Salicaceae	*Populus pruinosa*	GigaDB (http://dx.doi.org/10.5524/100319)	Populus pruinosa	-
Malpighiales	Salicaceae	*Populus tremula*	v3 PopGenIE (http://popgenie.org/)	v1.1	-
Malpighiales	Salicaceae	*Populus tremuloides*	v3 PopGenIE (http://popgenie.org/)	v1.1	-
Malpighiales	Salicaceae	*Populus trichocarpa*	NCBI	Pop_tri_v3	GCA_000002775.3
Malpighiales	Salicaceae	*Salix purpurea*	JGI12	Salix purpurea v1.0	-
Malpighiales	Salicaceae	*Salix suchowensis*	JGI GOLD	Gp0096061	https://gold.jgi.doe.gov/project?id=96061
Malvales	Malvaceae	*Corchorus capsularis*	NCBI	CCACVL1_1.0	GCA_001974805.1
Malvales	Malvaceae	*Corchorus olitorius*	NCBI	COLO4_1.0	GCA_001974825.1
Malvales	Malvaceae	*Gossypium arboreum*	NCBI	Gossypium_arboreum_v1.0	GCA_000612285.2
Malvales	Malvaceae	*Gossypium barbadense*	NCBI	GbV1.0	GCA_001856525.1
Malvales	Malvaceae	*Gossypium barbadense AA/DD*	COTTONGEN (https://www.cottongen.org/)	3-79_HAU v1	-
Malvales	Malvaceae	*Gossypium hirsutum*	NCBI	ASM250434v1	GCA_002504345.1
Malvales	Malvaceae	*Gossypium raimondii*	NCBI	Gr_v1.0	GCA_000331045.1
Malvales	Malvaceae	*Theobroma cacao*	NCBI	Criollo_cocoa_genome_V2	GCA_000208745.2
Malvales	Sterculiaceae	*Herrania umbratica*	NCBI	ASM216827v2	GCA_002168275.2
Myrtales	Lythraceae	*Punica granatum*	NCBI	ASM220158v1	GCA_002201585.1
Myrtales	Myrtaceae	*Eucalyptus grandis*	NCBI	Egrandis1_0	GCA_000612305.1
Oxalidales	Cephalotaceae	*Cephalotus follicularis*	NCBI	Cfol_1.0	GCA_001972305.1
Pandanales	Velloziaceae	*Xerophyta viscosa*	NCBI	ASM207613v1	GCA_002076135.1
Pinales	Pinaceae	*Picea abies*	NCBI	Pabies01	GCA_900067695.1
Poales	Bromeliaceae	*Ananas comosus*	NCBI	ASM154086v1	GCA_001540865.1
Poales	Poaceae	*Aegilops tauschii*	NCBI	ASM34733v1	GCA_000347335.1
Poales	Poaceae	*Brachypodium distachyon*	NCBI	Brachypodium_distachyon_v3.0	GCA_000005505.4
Poales	Poaceae	*Brachypodium stacei*	JGI12	Brachypodium stacei v1.1	
Poales	Poaceae	*Brachypodium sylvaticum*	JGI12	Brachypodium sylvaticum v1.1	
Poales	Poaceae	*Dichanthelium oligosanthes*	NCBI	ASM163321v2	GCA_001633215.2
Poales	Poaceae	*Eragrostis tef*	NCBI	ASM97063v1	GCA_000970635.1

Poales	Poaceae	*Hordeum vulgare*	NCBI	Hulless_Barley_ass.V2	GCA_004114815.1
Poales	Poaceae	*Leersia perrieri*	NCBI	Lperr_V1.4	GCA_000325765.3
Poales	Poaceae	*Lolium perenne*	NCBI	ASM173568v1	GCA_001735685.1
Poales	Poaceae	*Oropetium thomaeum*	NCBI	Oropetium_genomic_20141112	GCA_001182835.1
Poales	Poaceae	*Oryza barthii*	NCBI	O.barthii_v1.3	GCA_000182155.3
Poales	Poaceae	*Oryza brachyantha*	NCBI	Oryza_brachyantha.v1.4b	GCA_000231095.2
Poales	Poaceae	*Oryza glaberrima*	NCBI	Oryza_glaberrima_V1	GCA_000147395.2
Poales	Poaceae	*Oryza glumaepatula*	NCBI	Oryza_glumaepatula_v1.5	GCA_000576495.1
Poales	Poaceae	*Oryza indica*	Ensembl Plants v42	ASM465v1	-
Poales	Poaceae	*Oryza longistaminata*	NCBI	ASM151433v2	GCA_001514335.2
Poales	Poaceae	*Oryza meridionalis*	NCBI	Oryza_meridionalis_v1.3	GCA_000338895.2
Poales	Poaceae	*Oryza nivara*	NCBI	Oryza_nivara_v1.0	GCA_000576065.1
Poales	Poaceae	*Oryza punctata*	NCBI	Oryza_punctata_v1.2	GCA_000573905.1
Poales	Poaceae	*Oryza rufipogon*	NCBI	OR_W1943	GCA_000817225.1
Poales	Poaceae	*Oryza sativa*	NCBI	IRGSP-1.0	GCA_001433935.1
Poales	Poaceae	*Panicum hallii*	NCBI	PHallii_v3.1	GCA_002211085.2
Poales	Poaceae	*Panicum virgatum*	JGI12	Panicum virgatum v1.1	-
Poales	Poaceae	*Phyllostachys heterocycla*	National Center For Gene Research (http://server.ncgr.ac.cn/bamboo/down.php)	v1.0	-
Poales	Poaceae	*Secale cereale*	NCBI	Rye_Lo7_WGS_contigs	GCA_900079665.1
Poales	Poaceae	*Setaria italica*	NCBI	Setaria_italica_v2.0	GCA_000263155.2
Poales	Poaceae	*Setaria viridis*	NCBI	Setaria_viridis_v2.0	GCA_005286985.1
Poales	Poaceae	*Sorghum bicolor*	NCBI	Sorghum_bicolor_NCBIv3	GCA_000003195.3
Poales	Poaceae	*Triticum aestivum*	NCBI	iwgsc_refseqv1.0	GCA_900519105.1
Poales	Poaceae	*Triticum turgidum*	NCBI	WEWSeq v.1.0	GCA_002162155.1
Poales	Poaceae	*Triticum urartu*	NCBI	Tu2.0	GCA_003073215.1
Poales	Poaceae	*Zea mays*	NCBI	B73 RefGen_v4	GCA_000005005.6
Poales	Poaceae	*Zizania latifolia*	NCBI	Zizania_latifolia_v01	GCA_000418225.1
Poales	Poaceae	*Zoysia japonica*	NCBI	ASM160227v1	GCA_001602275.1
Poales	Poaceae	*Zoysia matrella*	NCBI	ASM160229v1	GCA_001602295.1
Poales	Poaceae	*Zoysia pacifica*	NCBI	ASM160231v1	GCA_001602315.1
Proteales	Nelumbonaceae	*Nelumbo nucifera*	NCBI	ASM303368v1	GCA_003033685.1
Proteales	Proteaceae	*Macadamia integrifolia*	NCBI	Macadamia Integrifolia v2	GCA_900631585.1
Ranunculales	Papaveraceae	*Macleaya cordata*	NCBI	MC_HNAU_1.0	GCA_002174775.1
Ranunculales	Ranunculaceae	*Aquilegia coerulea*	NCBI	Aquilegia_coerulea_v1	GCA_002738505.1
Rosales	Cannabaceae	*Humulus lupulus*	NCBI	hl_SW_version_1.0.fasta	GCA_000831365.1
Rosales	Moraceae	*Morus notabilis*	NCBI	ASM41409v2	GCA_000414095.2
Rosales	Rhamnaceae	*Ziziphus jujuba*	NCBI	ZizJuj_1.1	GCA_000826755.1
Rosales	Rosaceae	*Fragaria iinumae*	NCBI	FII_r1.1	GCA_000511975.1
Rosales	Rosaceae	*Fragaria nipponica*	NCBI	FNI_r1.1	GCA_000512025.1
Rosales	Rosaceae	*Fragaria nubicola*	NCBI	FNU_r1.1	GCA_000511995.1
Rosales	Rosaceae	*Fragaria orientalis*	NCBI	FOR_r1.1	GCA_000517285.1

Rosales	Rosaceae	*Fragaria vesca*	NCBI	FraVesHawaii_1.0	GCA_000184155.1
Rosales	Rosaceae	*Malus domestica*	NCBI	ASM211411v1	GCA_002114115.1
Rosales	Rosaceae	*Prunus avium*	NCBI	PAV_r1.0	GCA_002207925.1
Rosales	Rosaceae	*Prunus mume*	NCBI	P.mume_V1.0	GCA_000346735.1
Rosales	Rosaceae	*Prunus persica*	NCBI	Prunus_persica_NCBIv2	GCA_000346465.2
Rosales	Rosaceae	*Pyrus bretschneideri*	NCBI	Pbr_v1.0	GCA_000315295.1
Rosales	Rosaceae	*Rubus occidentalis*	Genome Database for Rosaceae	Rubus occidentalis Whole Genome Assembly v1.0 & Annotation v1	https://www.rosaceae.org/analysis/200
Sapindales	Anacardiaceae	*Anacardium occidentale*	JGI12	v0.9	-
Sapindales	Meliaceae	*Azadirachta indica*	NCBI	AzaInd2.1	GCA_000439995.3
Sapindales	Rutaceae	*Atalantia buxifolia*	CITRUS GENOME DATABASE	Atalantia buxifolia	-
Sapindales	Rutaceae	*Citrus clementina*	NCBI	Citrus_clementina_v1.0	GCA_000493195.1
Sapindales	Rutaceae	*Citrus grandis*	NCBI	ASM200692v1	GCA_002006925.1
Sapindales	Rutaceae	*Citrus ichangensis*	NCBI	ASM201397v2	GCA_002013975.2
Sapindales	Rutaceae	*Citrus medica*	NCBI	C_medica_denovo_2	GCA_002013955.2
Sapindales	Rutaceae	*Citrus sinensis*	NCBI	Csi_valencia_1.0	GCA_000317415.1
Sapindales	Sapindaceae	*Dimocarpus longan*	gigaDB (http://dx.doi.org/10.5524/100276)	Erigeron breviscapus	-
Saxifragales	Crassulaceae	*Kalanchoe fedtschenkoi*	NCBI	K_fedtschenkoi_M2_v1	GCA_002312845.1
Saxifragales	Crassulaceae	*Kalanchoe laxiflora*	JGI12	Kalanchoe laxiflora v1.1	-
Saxifragales	Crassulaceae	*Rhodiola crenulata*	GigaDB (http://dx.doi.org/10.5524/100301)	Rhodiola crenulata	-
Solanales	Convolvulaceae	*Ipomoea nil*	NCBI	Asagao_1.1	GCA_001879475.1
Solanales	Convolvulaceae	*Ipomoea trifida*	NCBI	ASM357666v1	GCA_003576665.1
Solanales	Solanaceae	*Capsicum annuum*	NCBI	Pepper Zunla 1 Ref_v1.0	GCA_000710875.1
Solanales	Solanaceae	*Capsicum baccatum*	NCBI	ASM227188v2	GCA_002271885.2
Solanales	Solanaceae	*Capsicum chinense*	NCBI	ASM227189v2	GCA_002271895.2
Solanales	Solanaceae	*Nicotiana attenuata*	NCBI	NIATTr2	GCA_001879085.1
Solanales	Solanaceae	*Nicotiana benthamiana*	NCBI	Ni_ben	GCA_000723945.1
Solanales	Solanaceae	*Nicotiana obtusifolia*	NCBI	NIOBT.version3	GCA_002018475.1
Solanales	Solanaceae	*Nicotiana sylvestris*	NCBI	Nsyl	GCA_000393655.1
Solanales	Solanaceae	*Nicotiana tabacum*	NCBI	Ntab-TN90	GCA_000715135.1
Solanales	Solanaceae	*Nicotiana tomentosiformis*	NCBI	Ntom_v01	GCA_000390325.2
Solanales	Solanaceae	*Petunia axillaris*	Sol Genomics Network	Petunia axillaris draft genome sequence v1.6.2	-
Solanales	Solanaceae	*Petunia inflata*	Sol Genomics Network	Petunia inflata draft genome sequence v1.0.1	-
Solanales	Solanaceae	*Solanum lycopersicum*	NCBI	SL3.0	GCA_000188115.3
Solanales	Solanaceae	*Solanum melongena*	Ensembl Plants v42	SolTub_3.0	-

Solanales	Solanaceae	*Solanum pennellii*	NCBI	SPENNV200	GCA_001406875.2
Solanales	Solanaceae	*Solanum pimpinellifolium*	NCBI	KAUST_Spi-LA0480_1.0	GCA_003660305.1
Solanales	Solanaceae	*Solanum tuberosum*	NCBI	SolTub_3.0	GCA_000226075.1
Vitales	Vitaceae	*Vitis vinifera*	NCBI	12X	GCA_000003745.2
Zingiberales	Musaceae	*Musa acuminata*	NCBI	ASM31385v2	GCA_000313855.2
Zingiberales	Musaceae	*Musa balbisiana*	Banana Genome Hub (https://banana-genome-hub.southgreen.fr/)	Musa balbisiana	-
Zingiberales	Musaceae	*Musa itinerans*	NCBI	ASM164941v1	GCA_001649415.1

Supplementary Table 16. Gene ontology enrichment of the 1,139 unique gene families (containing 3,390 genes) in Prickly waterlily based on the hypergeometric test.

GO	Type	Go Terms	Number of enriched genes	Number of genes in background	Adjusted P-value
GO:0000160	biological_process	phosphorelay signal transduction system	70	145	0.00E+00
GO:0006355	biological_process	regulation of transcription, DNA-templated	163	889	0.00E+00
GO:0007165	biological_process	signal transduction	87	295	0.00E+00
GO:0009889	biological_process	regulation of biosynthetic process	165	905	0.00E+00
GO:0010468	biological_process	regulation of gene expression	166	923	0.00E+00
GO:0010556	biological_process	regulation of macromolecule biosynthetic process	165	905	0.00E+00
GO:0019219	biological_process	regulation of nucleobase-containing compound metabolic process	163	904	0.00E+00
GO:0019222	biological_process	regulation of metabolic process	166	958	0.00E+00
GO:0031323	biological_process	regulation of cellular metabolic process	165	926	0.00E+00
GO:0031326	biological_process	regulation of cellular biosynthetic process	165	905	0.00E+00
GO:0050789	biological_process	regulation of biological process	229	1435	0.00E+00
GO:0050794	biological_process	regulation of cellular process	228	1396	0.00E+00
GO:0050896	biological_process	response to stimulus	140	731	0.00E+00
GO:0051171	biological_process	regulation of nitrogen compound metabolic process	163	904	0.00E+00
GO:0051252	biological_process	regulation of RNA metabolic process	163	895	0.00E+00
GO:0051716	biological_process	cellular response to stimulus	101	462	0.00E+00
GO:0060255	biological_process	regulation of macromolecule metabolic process	166	946	0.00E+00
GO:0065007	biological_process	biological regulation	232	1474	0.00E+00
GO:0080090	biological_process	regulation of primary metabolic process	165	926	0.00E+00
GO:2000112	biological_process	regulation of cellular macromolecule biosynthetic process	165	905	0.00E+00
GO:2001141	biological_process	regulation of RNA biosynthetic process	163	889	0.00E+00
GO:0006979	biological_process	response to oxidative stress	34	151	6.26E-08
GO:0006950	biological_process	response to stress	54	370	1.34E-05
GO:0006313	biological_process	transposition, DNA-mediated	7	13	1.01E-03
GO:0032196	biological_process	transposition	7	13	1.01E-03
GO:0006432	biological_process	phenylalanyl-tRNA aminoacylation	6	10	2.06E-03
GO:0007155	biological_process	cell adhesion	5	7	3.40E-03
GO:0022610	biological_process	biological adhesion	5	7	3.40E-03
GO:0006528	biological_process	asparagine metabolic process	6	11	4.26E-03
GO:0006529	biological_process	asparagine biosynthetic process	6	11	4.26E-03
GO:0006259	biological_process	DNA metabolic process	42	330	9.67E-03
GO:0006310	biological_process	DNA recombination	12	55	3.48E-02
GO:0005576	cellular_component	extracellular region	20	84	8.73E-05
GO:0009279	cellular_component	cell outer membrane	6	11	4.26E-03

GO:0005615	cellular_component	extracellular space	5	9	1.82E-02
GO:0044421	cellular_component	extracellular region part	5	9	1.82E-02
GO:0044462	cellular_component	external encapsulating structure part	6	14	2.32E-02
GO:0000155	molecular_function	phosphorelay sensor kinase activity	59	95	0.00E+00
GO:0001071	molecular_function	nucleic acid binding transcription factor activity	100	497	0.00E+00
GO:0003676	molecular_function	nucleic acid binding	323	2890	0.00E+00
GO:0003677	molecular_function	DNA binding	252	1545	0.00E+00
GO:0003700	molecular_function	transcription factor activity, sequence-specific DNA binding	100	497	0.00E+00
GO:0004673	molecular_function	protein histidine kinase activity	59	95	0.00E+00
GO:0004871	molecular_function	signal transducer activity	69	142	0.00E+00
GO:0004872	molecular_function	receptor activity	69	126	0.00E+00
GO:0016775	molecular_function	phosphotransferase activity, nitrogenous group as acceptor	59	95	0.00E+00
GO:0038023	molecular_function	signaling receptor activity	69	126	0.00E+00
GO:0043565	molecular_function	sequence-specific DNA binding	63	275	0.00E+00
GO:0060089	molecular_function	molecular transducer activity	69	142	0.00E+00
GO:0046983	molecular_function	protein dimerization activity	62	361	1.90E-09
GO:0005488	molecular_function	binding	783	9945	1.83E-08
GO:0004601	molecular_function	peroxidase activity	35	159	6.72E-08
GO:0016684	molecular_function	oxidoreductase activity, acting on peroxide as acceptor	35	159	6.72E-08
GO:0046906	molecular_function	tetrapyrrole binding	57	356	1.98E-07
GO:0020037	molecular_function	heme binding	54	344	1.08E-06
GO:0016209	molecular_function	antioxidant activity	35	188	6.62E-06
GO:0097159	molecular_function	organic cyclic compound binding	475	5729	1.53E-05
GO:1901363	molecular_function	heterocyclic compound binding	475	5729	1.53E-05
GO:0004888	molecular_function	transmembrane signaling receptor activity	10	25	3.41E-04
GO:0004803	molecular_function	transposase activity	7	13	1.01E-03
GO:0008237	molecular_function	metallopeptidase activity	23	126	1.71E-03
GO:0004826	molecular_function	phenylalanine-tRNA ligase activity	6	10	2.06E-03
GO:0008484	molecular_function	sulfuric ester hydrolase activity	5	7	3.40E-03
GO:0004066	molecular_function	asparagine synthase (glutamine-hydrolyzing) activity	6	11	4.26E-03
GO:0003674	molecular_function	molecular_function	1063	14928	4.90E-03
GO:0004970	molecular_function	ionotropic glutamate receptor activity	7	16	5.62E-03
GO:0005230	molecular_function	extracellular ligand-gated ion channel activity	7	16	5.62E-03
GO:0008066	molecular_function	glutamate receptor activity	7	16	5.62E-03
GO:0016884	molecular_function	carbon-nitrogen ligase activity, with glutamine as amido-N-donor	9	28	8.30E-03
GO:0015276	molecular_function	ligand-gated ion channel activity	7	17	8.98E-03
GO:0022834	molecular_function	ligand-gated channel activity	7	17	8.98E-03
GO:0030246	molecular_function	carbohydrate binding	21	133	3.41E-02
GO:0000049	molecular_function	tRNA binding	7	21	4.21E-02
GO:0000990	molecular_function	transcription factor activity, core RNA polymerase binding	7	21	4.21E-02
GO:0000996	molecular_function	core DNA-dependent RNA polymerase binding promoter specificity activity	7	21	4.21E-02

GO:0016987	molecular_function	sigma factor activity	7	21	4.21E-02

Supplementary Table 17. Gene ontology enrichment of the 1,016 unique gene families (containing 2,681) in rigid Hornwort based on the hypergeometric test.

GO	Type	Go Terms	Number of enriched genes	Number of genes in background	Adjusted P-value
GO:0006355	biological_process	regulation of transcription, DNA-templated	140	1305	0.00E+00
GO:0009889	biological_process	regulation of biosynthetic process	140	1328	0.00E+00
GO:0010468	biological_process	regulation of gene expression	140	1344	0.00E+00
GO:0010556	biological_process	regulation of macromolecule biosynthetic process	140	1328	0.00E+00
GO:0019219	biological_process	regulation of nucleobase-containing compound metabolic process	140	1320	0.00E+00
GO:0031323	biological_process	regulation of cellular metabolic process	140	1353	0.00E+00
GO:0031326	biological_process	regulation of cellular biosynthetic process	140	1328	0.00E+00
GO:0050789	biological_process	regulation of biological process	200	2005	0.00E+00
GO:0050794	biological_process	regulation of cellular process	189	1948	0.00E+00
GO:0051171	biological_process	regulation of nitrogen compound metabolic process	140	1320	0.00E+00
GO:0051252	biological_process	regulation of RNA metabolic process	140	1307	0.00E+00
GO:0060255	biological_process	regulation of macromolecule metabolic process	140	1370	0.00E+00
GO:0065007	biological_process	biological regulation	206	2064	0.00E+00
GO:0080090	biological_process	regulation of primary metabolic process	140	1353	0.00E+00
GO:2000112	biological_process	regulation of cellular macromolecule biosynthetic process	140	1328	0.00E+00
GO:2001141	biological_process	regulation of RNA biosynthetic process	140	1305	0.00E+00
GO:0019222	biological_process	regulation of metabolic process	140	1390	2.55E-14
GO:0040008	biological_process	regulation of growth	10	10	9.37E-12
GO:0045926	biological_process	negative regulation of growth	10	10	9.37E-12
GO:0050896	biological_process	response to stimulus	83	909	4.99E-06
GO:0071554	biological_process	cell wall organization or biogenesis	29	201	2.38E-05
GO:0042545	biological_process	cell wall modification	16	77	1.00E-04
GO:0006597	biological_process	spermine biosynthetic process	7	13	1.06E-04
GO:0008215	biological_process	spermine metabolic process	7	13	1.06E-04
GO:0010411	biological_process	xyloglucan metabolic process	13	60	6.47E-04
GO:0006952	biological_process	defense response	12	52	7.39E-04
GO:0042546	biological_process	cell wall biogenesis	13	65	1.64E-03
GO:0010383	biological_process	cell wall polysaccharide metabolic process	13	66	1.95E-03
GO:0010410	biological_process	hemicellulose metabolic process	13	66	1.95E-03
GO:0008216	biological_process	spermidine metabolic process	7	20	3.51E-03
GO:0008295	biological_process	spermidine biosynthetic process	7	20	3.51E-03
GO:0044085	biological_process	cellular component biogenesis	17	113	4.40E-03
GO:0006979	biological_process	response to oxidative stress	22	174	5.57E-03
GO:0007165	biological_process	signal transduction	36	371	1.03E-02
GO:0009309	biological_process	amine biosynthetic process	9	41	1.61E-02

GO:0042401	biological_process	cellular biogenic amine biosynthetic process	9	41	1.61E-02
GO:0048519	biological_process	negative regulation of biological process	10	52	2.23E-02
GO:0006596	biological_process	polyamine biosynthetic process	7	26	2.30E-02
GO:0044036	biological_process	cell wall macromolecule metabolic process	13	85	2.96E-02
GO:0006334	biological_process	nucleosome assembly	9	45	3.41E-02
GO:0034728	biological_process	nucleosome organization	9	46	4.05E-02
GO:0006595	biological_process	polyamine metabolic process	7	29	4.79E-02
GO:0005618	cellular_component	cell wall	14	68	5.39E-04
GO:0030312	cellular_component	external encapsulating structure	14	68	5.39E-04
GO:0005667	cellular_component	transcription factor complex	10	42	3.25E-03
GO:0000786	cellular_component	nucleosome	15	98	1.01E-02
GO:0032993	cellular_component	protein-DNA complex	15	98	1.01E-02
GO:0048046	cellular_component	apoplast	14	92	1.83E-02
GO:0001071	molecular_function	nucleic acid binding transcription factor activity	99	706	0.00E+00
GO:0003677	molecular_function	DNA binding	220	2147	0.00E+00
GO:0003700	molecular_function	transcription factor activity, sequence-specific DNA binding	99	706	0.00E+00
GO:0043531	molecular_function	ADP binding	40	131	0.00E+00
GO:0004857	molecular_function	enzyme inhibitor activity	36	160	1.33E-12
GO:0003676	molecular_function	nucleic acid binding	275	3825	9.26E-10
GO:0030234	molecular_function	enzyme regulator activity	44	302	1.31E-08
GO:0046983	molecular_function	protein dimerization activity	63	554	6.65E-08
GO:0004869	molecular_function	cysteine-type endopeptidase inhibitor activity	10	21	2.01E-06
GO:0043565	molecular_function	sequence-specific DNA binding	43	377	3.36E-05
GO:0005516	molecular_function	calmodulin binding	10	29	7.95E-05
GO:0030599	molecular_function	pectinesterase activity	16	77	1.00E-04
GO:0004014	molecular_function	adenosylmethionine decarboxylase activity	7	13	1.06E-04
GO:0004506	molecular_function	squalene monooxygenase activity	7	14	2.02E-04
GO:0016762	molecular_function	xyloglucan:xyloglucosyl transferase activity	14	68	5.39E-04
GO:0004601	molecular_function	peroxidase activity	24	183	1.39E-03
GO:0016684	molecular_function	oxidoreductase activity, acting on peroxide as acceptor	24	183	1.39E-03
GO:0004866	molecular_function	endopeptidase inhibitor activity	10	39	1.61E-03
GO:0030414	molecular_function	peptidase inhibitor activity	10	39	1.61E-03
GO:0061134	molecular_function	peptidase regulator activity	10	39	1.61E-03
GO:0061135	molecular_function	endopeptidase regulator activity	10	39	1.61E-03
GO:0016209	molecular_function	antioxidant activity	26	213	2.23E-03
GO:0008171	molecular_function	O-methyltransferase activity	10	43	4.05E-03
GO:0097159	molecular_function	organic cyclic compound binding	440	7723	8.29E-03
GO:1901363	molecular_function	heterocyclic compound binding	440	7723	8.29E-03
GO:0005488	molecular_function	binding	727	13601	1.64E-02
GO:0020037	molecular_function	heme binding	44	499	1.70E-02
GO:0046906	molecular_function	tetrapyrrole binding	44	500	1.78E-02
GO:0005094	molecular_function	Rho GDP-dissociation inhibitor activity	5	13	3.06E-02
GO:0052689	molecular_function	carboxylic ester hydrolase activity	16	125	4.99E-02

Supplementary Table 18. Gene ontology enrichment of the 6,501 expanded gene families (containing 22,722 genes) in Prickly waterlily based on the hypergeometric test.

GO	Type	Go Terms	Number of enriched genes	Number of genes in background	Adjusted P-value
GO:0006464	biological_process	cellular protein modification process	616	1316	1.14E-13
GO:0036211	biological_process	protein modification process	616	1316	1.14E-13
GO:0044267	biological_process	cellular protein metabolic process	834	1860	3.13E-13
GO:0006468	biological_process	protein phosphorylation	438	918	8.59E-11
GO:0043412	biological_process	macromolecule modification	628	1426	8.22E-08
GO:0016310	biological_process	phosphorylation	450	990	2.56E-07
GO:0006793	biological_process	phosphorus metabolic process	482	1072	4.05E-07
GO:0006796	biological_process	phosphate-containing compound metabolic process	482	1072	4.05E-07
GO:0019538	biological_process	protein metabolic process	989	2381	3.19E-06
GO:0009250	biological_process	glucan biosynthetic process	41	58	2.85E-05
GO:0034637	biological_process	cellular carbohydrate biosynthetic process	62	100	4.17E-05
GO:0030244	biological_process	cellulose biosynthetic process	23	29	7.34E-04
GO:0009314	biological_process	response to radiation	12	12	1.37E-03
GO:0009416	biological_process	response to light stimulus	12	12	1.37E-03
GO:0042545	biological_process	cell wall modification	44	71	2.59E-03
GO:0033692	biological_process	cellular polysaccharide biosynthetic process	41	65	2.77E-03
GO:0000271	biological_process	polysaccharide biosynthetic process	42	68	4.62E-03
GO:0030243	biological_process	cellulose metabolic process	23	31	5.22E-03
GO:0044260	biological_process	cellular macromolecule metabolic process	1189	2999	6.48E-03
GO:0006544	biological_process	glycine metabolic process	16	20	2.19E-02
GO:0009581	biological_process	detection of external stimulus	9	9	2.86E-02
GO:0009582	biological_process	detection of abiotic stimulus	9	9	2.86E-02
GO:0009583	biological_process	detection of light stimulus	9	9	2.86E-02
GO:0009584	biological_process	detection of visible light	9	9	2.86E-02
GO:0018298	biological_process	protein-chromophore linkage	9	9	2.86E-02
GO:0051606	biological_process	detection of stimulus	9	9	2.86E-02
GO:0044262	biological_process	cellular carbohydrate metabolic process	81	159	2.97E-02
GO:0006414	biological_process	translational elongation	26	39	2.99E-02
GO:0005984	biological_process	disaccharide metabolic process	31	49	3.00E-02
GO:0016051	biological_process	carbohydrate biosynthetic process	68	130	3.71E-02
GO:0006888	biological_process	ER to Golgi vesicle-mediated transport	15	19	4.86E-02
GO:0000159	cellular_component	protein phosphatase type 2A complex	21	23	1.64E-05
GO:0030117	cellular_component	membrane coat	39	54	2.29E-05
GO:0008287	cellular_component	protein serine/threonine phosphatase complex	21	24	8.56E-05
GO:0043227	cellular_component	membrane-bounded organelle	267	594	2.03E-03

GO:0043231	cellular_component	intracellular membrane-bounded organelle	267	594	2.03E-03
GO:0043226	cellular_component	organelle	434	1024	6.76E-03
GO:0043229	cellular_component	intracellular organelle	434	1024	6.76E-03
GO:0044433	cellular_component	cytoplasmic vesicle part	26	37	7.37E-03
GO:0044464	cellular_component	cell part	902	2253	1.69E-02
GO:0044424	cellular_component	intracellular part	836	2082	2.10E-02
GO:0030120	cellular_component	vesicle coat	22	31	2.43E-02
GO:0030234	molecular_function	enzyme regulator activity	144	246	2.53E-10
GO:0035251	molecular_function	UDP-glucosyltransferase activity	47	67	4.97E-06
GO:0016301	molecular_function	kinase activity	550	1261	6.18E-06
GO:0004672	molecular_function	protein kinase activity	454	1021	6.96E-06
GO:0046527	molecular_function	glucosyltransferase activity	47	68	1.04E-05
GO:0005488	molecular_function	binding	3779	9945	1.93E-05
GO:0016773	molecular_function	phosphotransferase activity, alcohol group as acceptor	513	1184	5.28E-05
GO:0004857	molecular_function	enzyme inhibitor activity	71	123	2.86E-04
GO:0016759	molecular_function	cellulose synthase activity	23	29	7.34E-04
GO:0016760	molecular_function	cellulose synthase (UDP-forming) activity	23	29	7.34E-04
GO:0019208	molecular_function	phosphatase regulator activity	23	29	7.34E-04
GO:0030599	molecular_function	pectinesterase activity	44	71	2.59E-03
GO:0019888	molecular_function	protein phosphatase regulator activity	21	27	3.52E-03
GO:0052689	molecular_function	carboxylic ester hydrolase activity	65	117	4.52E-03
GO:0008289	molecular_function	lipid binding	50	85	5.28E-03
GO:0005509	molecular_function	calcium ion binding	123	252	8.27E-03
GO:0004702	molecular_function	receptor signaling protein serine/threonine kinase activity	14	16	9.51E-03
GO:0004707	molecular_function	MAP kinase activity	14	16	9.51E-03
GO:0005057	molecular_function	receptor signaling protein activity	14	16	9.51E-03
GO:0097159	molecular_function	organic cyclic compound binding	2202	5729	1.01E-02
GO:1901363	molecular_function	heterocyclic compound binding	2202	5729	1.01E-02
GO:0003723	molecular_function	RNA binding	248	559	1.26E-02
GO:0003676	molecular_function	nucleic acid binding	1144	2890	1.30E-02
GO:0008194	molecular_function	UDP-glycosyltransferase activity	62	114	1.65E-02
GO:0005516	molecular_function	calmodulin binding	16	20	2.19E-02
GO:0016307	molecular_function	phosphatidylinositol phosphate kinase activity	16	20	2.19E-02
GO:0004743	molecular_function	pyruvate kinase activity	18	24	3.43E-02
GO:0030955	molecular_function	potassium ion binding	18	24	3.43E-02
GO:0031420	molecular_function	alkali metal ion binding	18	24	3.43E-02
GO:0016772	molecular_function	transferase activity, transferring phosphorus-containing groups	605	1483	3.48E-02

Supplementary Table 19. Gene ontology enrichment of the 3,473 expanded gene families (9,535) in rigid Hornwort based on the hypergeometric test.

GO	Type	Go Terms	Number of enriched genes	Number of genes in background	Adjusted P-value
GO:0031323	biological_process	regulation of cellular metabolic process	1052	1353	7.48E-13
GO:0080090	biological_process	regulation of primary metabolic process	1050	1353	2.04E-12
GO:0019222	biological_process	regulation of metabolic process	1076	1390	3.29E-12
GO:0044267	biological_process	cellular protein metabolic process	2036	2727	3.39E-12
GO:0051252	biological_process	regulation of RNA metabolic process	1015	1307	4.39E-12
GO:0009889	biological_process	regulation of biosynthetic process	1030	1328	5.08E-12
GO:0010556	biological_process	regulation of macromolecule biosynthetic process	1030	1328	5.08E-12
GO:0031326	biological_process	regulation of cellular biosynthetic process	1030	1328	5.08E-12
GO:2000112	biological_process	regulation of cellular macromolecule biosynthetic process	1030	1328	5.08E-12
GO:0006355	biological_process	regulation of transcription, DNA-templated	1013	1305	5.73E-12
GO:2001141	biological_process	regulation of RNA biosynthetic process	1013	1305	5.73E-12
GO:0019219	biological_process	regulation of nucleobase-containing compound metabolic process	1023	1320	9.12E-12
GO:0051171	biological_process	regulation of nitrogen compound metabolic process	1023	1320	9.12E-12
GO:0006793	biological_process	phosphorus metabolic process	1298	1703	2.66E-11
GO:0006796	biological_process	phosphate-containing compound metabolic process	1298	1703	2.66E-11
GO:0060255	biological_process	regulation of macromolecule metabolic process	1057	1370	2.88E-11
GO:0010468	biological_process	regulation of gene expression	1038	1344	3.05E-11
GO:0019538	biological_process	protein metabolic process	2459	3338	8.88E-11
GO:0016310	biological_process	phosphorylation	1221	1604	3.00E-10
GO:0006468	biological_process	protein phosphorylation	1176	1550	3.86E-09
GO:0006464	biological_process	cellular protein modification process	1537	2065	5.54E-08
GO:0036211	biological_process	protein modification process	1537	2065	5.54E-08
GO:0051649	biological_process	establishment of localization in cell	324	399	1.78E-06
GO:0006886	biological_process	intracellular protein transport	210	249	2.38E-06
GO:0015031	biological_process	protein transport	261	317	4.66E-06
GO:0045184	biological_process	establishment of protein localization	261	317	4.66E-06
GO:0046907	biological_process	intracellular transport	284	353	8.62E-05
GO:0015672	biological_process	monovalent inorganic cation transport	131	152	1.36E-04
GO:0043412	biological_process	macromolecule modification	1568	2157	9.47E-04
GO:0016192	biological_process	vesicle-mediated transport	223	277	1.58E-03
GO:0009056	biological_process	catabolic process	409	533	4.25E-03
GO:0006739	biological_process	NADP metabolic process	28	28	1.05E-02
GO:0009698	biological_process	phenylpropanoid metabolic process	24	24	4.82E-02
GO:0009808	biological_process	lignin metabolic process	24	24	4.82E-02
GO:0046271	biological_process	phenylpropanoid catabolic process	24	24	4.82E-02
GO:0046274	biological_process	lignin catabolic process	24	24	4.82E-02

GO:0005575	cellular_component	cellular_component	3636	4983	1.99E-13
GO:0044464	cellular_component	cell part	2124	2907	7.03E-07
GO:0044444	cellular_component	cytoplasmic part	840	1103	1.28E-06
GO:0044424	cellular_component	intracellular part	1960	2684	4.54E-06
GO:0030117	cellular_component	membrane coat	65	68	1.37E-05
GO:0044433	cellular_component	cytoplasmic vesicle part	45	46	2.45E-04
GO:0043234	cellular_component	protein complex	658	873	8.91E-04
GO:0030120	cellular_component	vesicle coat	39	40	2.11E-03
GO:0016020	cellular_component	membrane	940	1274	3.29E-03
GO:0043226	cellular_component	organelle	1022	1392	5.14E-03
GO:0043229	cellular_component	intracellular organelle	1022	1392	5.14E-03
GO:0043227	cellular_component	membrane-bounded organelle	653	877	1.40E-02
GO:0043231	cellular_component	intracellular membrane-bounded organelle	653	877	1.40E-02
GO:0003676	molecular_function	nucleic acid binding	2828	3825	0.00E+00
GO:0097159	molecular_function	organic cyclic compound binding	5583	7723	0.00E+00
GO:1901363	molecular_function	heterocyclic compound binding	5583	7723	0.00E+00
GO:0035639	molecular_function	purine ribonucleoside triphosphate binding	2338	3172	2.85E-10
GO:0005524	molecular_function	ATP binding	2059	2778	3.08E-10
GO:0004672	molecular_function	protein kinase activity	1209	1588	3.67E-10
GO:0016301	molecular_function	kinase activity	1395	1848	5.14E-10
GO:0016773	molecular_function	phosphotransferase activity, alcohol group as acceptor	1339	1774	1.63E-09
GO:0003677	molecular_function	DNA binding	1589	2147	4.16E-07
GO:0016772	molecular_function	transferase activity, transferring phosphorus-containing groups	1527	2077	1.68E-05
GO:0005488	molecular_function	binding	9452	13601	7.14E-04
GO:0019843	molecular_function	rRNA binding	34	34	1.06E-03
GO:0003723	molecular_function	RNA binding	478	625	1.51E-03
GO:0001071	molecular_function	nucleic acid binding transcription factor activity	535	706	2.77E-03
GO:0003700	molecular_function	transcription factor activity, sequence-specific DNA binding	535	706	2.77E-03
GO:0036094	molecular_function	small molecule binding	2676	3753	4.04E-03
GO:0005200	molecular_function	structural constituent of cytoskeleton	30	30	4.89E-03
GO:0016740	molecular_function	transferase activity	2546	3568	5.11E-03
GO:0000166	molecular_function	nucleotide binding	2553	3582	8.25E-03
GO:1901265	molecular_function	nucleoside phosphate binding	2553	3582	8.25E-03
GO:0017076	molecular_function	purine nucleotide binding	2374	3325	8.55E-03
GO:0032553	molecular_function	ribonucleotide binding	2358	3307	1.57E-02
GO:0032555	molecular_function	purine ribonucleotide binding	2358	3307	1.57E-02
GO:0030554	molecular_function	adenyl nucleotide binding	2085	2920	2.95E-02
GO:0032559	molecular_function	adenyl ribonucleotide binding	2079	2913	3.60E-02
GO:0004190	molecular_function	aspartic-type endopeptidase activity	76	88	3.67E-02
GO:0070001	molecular_function	aspartic-type peptidase activity	76	88	3.67E-02
GO:0043565	molecular_function	sequence-specific DNA binding	291	377	3.70E-02
GO:0052716	molecular_function	hydroquinone:oxygen oxidoreductase activity	24	24	4.82E-02

Supplementary Table 20. Gene ontology enrichment of the 5,927 contracted gene families (472) in prickly waterlily based on the hypergeometric test.

GO	Type	Go Terms	Number of enriched genes	Number of genes in background	Adjusted P-value
GO:0008037	biological_process	cell recognition	19	32	0.00E+00
GO:0048544	biological_process	recognition of pollen	19	32	0.00E+00
GO:0048610	biological_process	obsolete cellular process involved in reproduction	19	37	0.00E+00
GO:0022414	biological_process	reproductive process	19	42	0.00E+00
GO:0044702	biological_process	single organism reproductive process	19	42	0.00E+00
GO:0044699	biological_process	single-organism process	19	80	7.11E-15
GO:0070588	biological_process	calcium ion transmembrane transport	7	7	1.78E-11
GO:0055085	biological_process	transmembrane transport	51	940	5.31E-11
GO:0006816	biological_process	calcium ion transport	7	16	1.78E-07
GO:0010215	biological_process	cellulose microfibril organization	5	7	1.17E-06
GO:0016049	biological_process	cell growth	5	7	1.17E-06
GO:0030198	biological_process	extracellular matrix organization	5	7	1.17E-06
GO:0040007	biological_process	growth	5	7	1.17E-06
GO:0043062	biological_process	extracellular structure organization	5	7	1.17E-06
GO:0008150	biological_process	biological_process	260	12288	6.26E-04
GO:0070838	biological_process	divalent metal ion transport	7	48	6.98E-04
GO:0072511	biological_process	divalent inorganic cation transport	7	48	6.98E-04
GO:0006810	biological_process	transport	55	1738	8.09E-04
GO:0051234	biological_process	establishment of localization	55	1738	8.09E-04
GO:0055114	biological_process	oxidation-reduction process	49	1554	2.56E-03
GO:0005975	biological_process	carbohydrate metabolic process	37	1069	3.31E-03
GO:0016020	cellular_component	membrane	54	1274	1.70E-08
GO:0031224	cellular_component	intrinsic component of membrane	44	1058	9.48E-07
GO:0031225	cellular_component	anchored component of membrane	5	12	6.39E-06
GO:0016021	cellular_component	integral component of membrane	39	1046	6.52E-05
GO:0044425	cellular_component	membrane part	44	1365	5.81E-04
GO:0004888	molecular_function	transmembrane signaling receptor activity	17	22	0.00E+00
GO:0004970	molecular_function	ionotropic glutamate receptor activity	16	18	0.00E+00
GO:0005230	molecular_function	extracellular ligand-gated ion channel activity	16	18	0.00E+00
GO:0008066	molecular_function	glutamate receptor activity	16	18	0.00E+00
GO:0015276	molecular_function	ligand-gated ion channel activity	16	18	0.00E+00
GO:0022834	molecular_function	ligand-gated channel activity	16	18	0.00E+00
GO:0004872	molecular_function	receptor activity	17	64	6.75E-14
GO:0038023	molecular_function	signaling receptor activity	17	64	6.75E-14
GO:0022836	molecular_function	gated channel activity	16	56	1.35E-13
GO:0004674	molecular_function	protein serine/threonine kinase activity	19	97	6.08E-13
GO:0003824	molecular_function	catalytic activity	242	9317	7.26E-13
GO:0004871	molecular_function	signal transducer activity	17	86	1.38E-11

GO:0060089	molecular_function	molecular transducer activity	17	86	1.38E-11
GO:0005216	molecular_function	ion channel activity	16	79	4.79E-11
GO:0022838	molecular_function	substrate-specific channel activity	16	79	4.79E-11
GO:0005388	molecular_function	calcium-transporting ATPase activity	7	8	3.34E-10
GO:0004553	molecular_function	hydrolase activity, hydrolyzing O-glycosyl compounds	34	489	1.30E-09
GO:0022857	molecular_function	transmembrane transporter activity	49	949	2.09E-09
GO:0016798	molecular_function	hydrolase activity, acting on glycosyl bonds	34	518	6.14E-09
GO:0015267	molecular_function	channel activity	17	128	1.07E-08
GO:0022803	molecular_function	passive transmembrane transporter activity	17	128	1.07E-08
GO:0005215	molecular_function	transporter activity	49	1015	2.13E-08
GO:0015075	molecular_function	ion transmembrane transporter activity	30	440	3.38E-08
GO:0022891	molecular_function	substrate-specific transmembrane transporter activity	30	463	1.13E-07
GO:0015085	molecular_function	calcium ion transmembrane transporter activity	7	17	7.06E-07
GO:0022892	molecular_function	substrate-specific transporter activity	30	513	1.19E-06
GO:0015662	molecular_function	ATPase activity, coupled to transmembrane movement of ions, phosphorylative mechanism	7	21	3.97E-06
GO:0003690	molecular_function	double-stranded DNA binding	9	50	1.71E-05
GO:0019829	molecular_function	cation-transporting ATPase activity	7	29	4.71E-05
GO:0043566	molecular_function	structure-specific DNA binding	9	68	2.53E-04
GO:0016758	molecular_function	transferase activity, transferring hexosyl groups	23	431	2.77E-04
GO:0042625	molecular_function	ATPase coupled ion transmembrane transporter activity	7	42	6.66E-04
GO:0045735	molecular_function	nutrient reservoir activity	5	17	7.05E-04
GO:0072509	molecular_function	divalent inorganic cation transmembrane transporter activity	7	49	1.90E-03
GO:0016491	molecular_function	oxidoreductase activity	53	1669	2.50E-03
GO:0030246	molecular_function	carbohydrate binding	12	163	2.97E-03
GO:0071949	molecular_function	FAD binding	7	56	4.62E-03
GO:0015298	molecular_function	solute:cation antiporter activity	6	44	9.54E-03
GO:0015299	molecular_function	solute:proton antiporter activity	6	44	9.54E-03
GO:0005516	molecular_function	calmodulin binding	5	29	1.13E-02
GO:0022804	molecular_function	active transmembrane transporter activity	15	283	1.47E-02
GO:0022890	molecular_function	inorganic cation transmembrane transporter activity	14	253	1.53E-02
GO:0016740	molecular_function	transferase activity	89	3568	4.12E-02

Supplementary Table 21. Gene ontology enrichment of the 5,024 contracted gene families (containing 1,332 genes) in rigid Hornwort based on the hypergeometric test.

GO	Type	Go Terms	Number of enriched genes	Number of genes in background	Adjusted P-value
GO:0055085	biological_process	transmembrane transport	139	763	0.00E+00
GO:0006810	biological_process	transport	178	1366	0.00E+00
GO:0051234	biological_process	establishment of localization	178	1366	0.00E+00
GO:0015914	biological_process	phospholipid transport	10	12	4.12E-09
GO:0006813	biological_process	potassium ion transport	16	38	2.67E-08
GO:0006869	biological_process	lipid transport	12	23	1.98E-07
GO:0006855	biological_process	drug transmembrane transport	14	33	2.96E-07
GO:0015893	biological_process	drug transport	14	33	2.96E-07
GO:0006468	biological_process	protein phosphorylation	104	918	4.64E-07
GO:0071804	biological_process	cellular potassium ion transport	11	24	5.13E-06
GO:0071805	biological_process	potassium ion transmembrane transport	11	24	5.13E-06
GO:0006811	biological_process	ion transport	53	383	5.99E-06
GO:0016310	biological_process	phosphorylation	106	990	6.36E-06
GO:0006559	biological_process	L-phenylalanine catabolic process	7	9	8.69E-06
GO:0006793	biological_process	phosphorus metabolic process	111	1072	1.75E-05
GO:0006796	biological_process	phosphate-containing compound metabolic process	111	1072	1.75E-05
GO:0006820	biological_process	anion transport	15	50	1.94E-05
GO:0008150	biological_process	biological_process	693	9594	2.65E-05
GO:0071702	biological_process	organic substance transport	15	52	3.42E-05
GO:0019538	biological_process	protein metabolic process	205	2381	2.22E-04
GO:0006464	biological_process	cellular protein modification process	124	1316	4.63E-04
GO:0036211	biological_process	protein modification process	124	1316	4.63E-04
GO:0030001	biological_process	metal ion transport	28	183	9.80E-04
GO:0008272	biological_process	sulfate transport	7	15	1.10E-03
GO:0009719	biological_process	response to endogenous stimulus	14	60	1.22E-03
GO:0009725	biological_process	response to hormone	14	60	1.22E-03
GO:0010033	biological_process	response to organic substance	14	60	1.22E-03
GO:0009074	biological_process	aromatic amino acid family catabolic process	7	17	2.97E-03
GO:0015672	biological_process	monovalent inorganic cation transport	21	128	3.90E-03
GO:0043412	biological_process	macromolecule modification	127	1426	4.97E-03
GO:0015698	biological_process	inorganic anion transport	11	46	7.40E-03
GO:0008037	biological_process	cell recognition	6	14	8.12E-03
GO:0048544	biological_process	recognition of pollen	6	14	8.12E-03

GO:0034220	biological_process	ion transmembrane transport	20	127	1.03E-02
GO:0006857	biological_process	oligopeptide transport	6	15	1.28E-02
GO:0006558	biological_process	L-phenylalanine metabolic process	7	21	1.41E-02
GO:0015833	biological_process	peptide transport	6	16	1.94E-02
GO:0048610	biological_process	obsolete cellular process involved in reproduction	6	16	1.94E-02
GO:0044267	biological_process	cellular protein metabolic process	155	1860	2.07E-02
GO:0042221	biological_process	response to chemical	15	86	2.13E-02
GO:0006508	biological_process	proteolysis	52	507	3.43E-02
GO:0016020	cellular_component	membrane	146	1008	0.00E+00
GO:0005575	cellular_component	cellular_component	316	3901	1.13E-05
GO:0031224	cellular_component	intrinsic component of membrane	89	890	1.82E-04
GO:0016021	cellular_component	integral component of membrane	87	876	2.93E-04
GO:0005743	cellular_component	mitochondrial inner membrane	5	17	3.50E-02
GO:0019866	cellular_component	organelle inner membrane	5	17	3.50E-02
GO:0005215	molecular_function	transporter activity	149	813	0.00E+00
GO:0022857	molecular_function	transmembrane transporter activity	134	753	0.00E+00
GO:0043531	molecular_function	ADP binding	20	31	0.00E+00
GO:0022892	molecular_function	substrate-specific transporter activity	70	398	9.11E-13
GO:0022804	molecular_function	active transmembrane transporter activity	43	212	1.27E-09
GO:0004012	molecular_function	phospholipid-translocating ATPase activity	10	11	1.29E-09
GO:0005548	molecular_function	phospholipid transporter activity	10	11	1.29E-09
GO:0015075	molecular_function	ion transmembrane transporter activity	57	336	1.36E-09
GO:0015291	molecular_function	secondary active transmembrane transporter activity	27	95	2.84E-09
GO:0015079	molecular_function	potassium ion transmembrane transporter activity	16	34	5.98E-09
GO:0046873	molecular_function	metal ion transmembrane transporter activity	27	98	6.27E-09
GO:0005319	molecular_function	lipid transporter activity	10	12	7.29E-09
GO:0022891	molecular_function	substrate-specific transmembrane transporter activity	58	361	8.51E-09
GO:0043492	molecular_function	ATPase activity, coupled to movement of substances	27	109	8.67E-08
GO:0004553	molecular_function	hydrolase activity, hydrolyzing O-glycosyl compounds	56	379	4.38E-07
GO:0032559	molecular_function	adenyl ribonucleotide binding	203	2153	4.93E-07
GO:0015238	molecular_function	drug transmembrane transporter activity	14	33	5.24E-07
GO:0090484	molecular_function	drug transporter activity	14	33	5.24E-07
GO:0030554	molecular_function	adenyl nucleotide binding	203	2160	6.43E-07
GO:0016798	molecular_function	hydrolase activity, acting on glycosyl bonds	56	400	3.06E-06
GO:0005216	molecular_function	ion channel activity	16	49	3.63E-06
GO:0022838	molecular_function	substrate-specific channel activity	16	49	3.63E-06

GO:0004252	molecular_function	serine-type endopeptidase activity	29	147	5.39E-06
GO:0016787	molecular_function	hydrolase activity	218	2425	6.33E-06
GO:0010333	molecular_function	terpene synthase activity	6	6	7.50E-06
GO:0032553	molecular_function	ribonucleotide binding	217	2443	1.78E-05
GO:0032555	molecular_function	purine ribonucleotide binding	217	2443	1.78E-05
GO:0000287	molecular_function	magnesium ion binding	24	113	1.80E-05
GO:0017076	molecular_function	purine nucleotide binding	217	2456	2.69E-05
GO:0004672	molecular_function	protein kinase activity	107	1021	2.87E-05
GO:0016887	molecular_function	ATPase activity	46	322	2.94E-05
GO:0003824	molecular_function	catalytic activity	571	7665	4.09E-05
GO:0016841	molecular_function	ammonia-lyase activity	8	14	6.48E-05
GO:0033926	molecular_function	glycopeptide alpha-N-acetylgalactosaminidase activity	7	11	1.26E-04
GO:0000166	molecular_function	nucleotide binding	229	2675	1.35E-04
GO:1901265	molecular_function	nucleoside phosphate binding	229	2675	1.35E-04
GO:0008236	molecular_function	serine-type peptidase activity	34	219	1.59E-04
GO:0017171	molecular_function	serine hydrolase activity	34	219	1.59E-04
GO:0042623	molecular_function	ATPase activity, coupled	28	163	1.76E-04
GO:0008271	molecular_function	secondary active sulfate transmembrane transporter activity	6	8	1.88E-04
GO:0022836	molecular_function	gated channel activity	12	37	2.07E-04
GO:0016773	molecular_function	phosphotransferase activity, alcohol group as acceptor	116	1184	2.68E-04
GO:0015267	molecular_function	channel activity	18	84	5.40E-04
GO:0022803	molecular_function	passive transmembrane transporter activity	18	84	5.40E-04
GO:0016840	molecular_function	carbon-nitrogen lyase activity	10	28	5.61E-04
GO:0036094	molecular_function	small molecule binding	237	2849	7.27E-04
GO:0008509	molecular_function	anion transmembrane transporter activity	13	48	7.45E-04
GO:0005524	molecular_function	ATP binding	183	2118	1.21E-03
GO:0015103	molecular_function	inorganic anion transmembrane transporter activity	12	44	1.55E-03
GO:0005506	molecular_function	iron ion binding	32	222	1.56E-03
GO:0015116	molecular_function	sulfate transmembrane transporter activity	7	15	1.95E-03
GO:0005249	molecular_function	voltage-gated potassium channel activity	5	7	2.21E-03
GO:0022843	molecular_function	voltage-gated cation channel activity	5	7	2.21E-03
GO:0015297	molecular_function	antiporter activity	16	76	2.27E-03
GO:0016301	molecular_function	kinase activity	117	1261	3.03E-03
GO:0022890	molecular_function	inorganic cation transmembrane transporter activity	30	211	3.73E-03
GO:0016705	molecular_function	oxidoreductase activity, acting on paired donors, with incorporation or reduction of molecular oxygen	28	193	4.67E-03

GO:0008324	molecular_function	cation transmembrane transporter activity	35	267	4.96E-03
GO:0004175	molecular_function	endopeptidase activity	38	314	1.36E-02
GO:0035639	molecular_function	purine ribonucleoside triphosphate binding	197	2408	1.43E-02
GO:0016838	molecular_function	carbon-oxygen lyase activity, acting on phosphates	6	14	1.44E-02
GO:0016820	molecular_function	hydrolase activity, acting on acid anhydrides, catalyzing transmembrane movement of substances	17	98	1.67E-02
GO:0042626	molecular_function	ATPase activity, coupled to transmembrane movement of substances	17	98	1.67E-02
GO:0004674	molecular_function	protein serine/threonine kinase activity	12	59	3.37E-02
GO:0004970	molecular_function	ionotropic glutamate receptor activity	6	16	3.43E-02
GO:0005230	molecular_function	extracellular ligand-gated ion channel activity	6	16	3.43E-02
GO:0008066	molecular_function	glutamate receptor activity	6	16	3.43E-02
GO:0017111	molecular_function	nucleoside-triphosphatase activity	65	659	3.89E-02
GO:0016772	molecular_function	transferase activity, transferring phosphorus-containing groups	127	1483	4.57E-02

Supplementary Table 22. Summary of the data source and heterozygosity rate of 7 species.

Species	Data source	Accession number	Total base (G)	Mapping depth (×)	Heterozygosity (%)
A. trichopoda	NCBI	SRR954305<br>SRR954306<br>SRR954307	64.7	55.5	0.18
E. ferox	-	-	47.3	61.8	0.02
	-	-	137.4	148	0.07
P. equestris	NCBI	SRR5809592	121.4	61.5	0.84
L. chinense	NCBI	SRR6876702	27.9	13.7	0.30
C. kanehirae	NCBI	SRR7416916	103.1	57.3	0.46
C. demersum	-	-	46.4	45.8	0.62
V. vinifera	NCBI	SRR8506855	30.1	48.19	0.93

Supplementary References

1. De Bie, T., Cristianini, N., Demuth, J. P. & Hahn, M. W. CAFE: a computational tool for the study of gene family evolution. *Bioinformatics* **22**, 1269-1271, doi:10.1093/bioinformatics/btl097 (2006).
2. Sayyari, E. & Mirarab, S. Testing for Polytomies in Phylogenetic Species Trees Using Quartet Frequencies. *Genes (Basel)* **9**, doi:10.3390/genes9030132 (2018).
3. Chaw, S.-M. *et al.* Stout camphor tree genome fills gaps in understanding of flowering plant genome evolution. *Nature plants* **5**, 63 (2019).
4. Chen, J. *et al.* Liriodendron genome sheds light on angiosperm phylogeny and species–pair differentiation. *Nature plants* **5**, 18 (2019).
5. Rendon-Anaya, M. *et al.* The avocado genome informs deep angiosperm phylogeny, highlights introgressive hybridization, and reveals pathogen-influenced gene space adaptation. *Proc Natl Acad Sci U S A* **116**, 17081-17089, doi:10.1073/pnas.1822129116 (2019).
6. Hu, L. S. *et al.* The chromosome-scale reference genome of black pepper provides insight into piperine biosynthesis. *Nature Communications* **10**, doi:ARTN 470210.1038/s41467-019-12607-6 (2019).
7. Solis-Lemus, C., Bastide, P. & Ane, C. PhyloNetworks: A Package for Phylogenetic Networks. *Mol Biol Evol* **34**, 3292-3298, doi:10.1093/molbev/msx235 (2017).
8. Wang, K. *et al.* Incomplete lineage sorting rather than hybridization explains the inconsistent phylogeny of the wisent. *Communications biology* **1**, 169 (2018).
9. Estep, M. C. *et al.* Allopolyploidy, diversification, and the Miocene grassland expansion. *Proc Natl Acad Sci U S A* **111**, 15149-15154, doi:10.1073/pnas.1404177111 (2014).
10. Wu, S., Han, B. & Jiao, Y. Genetic Contribution of Paleopolyploidy to Adaptive Evolution in Angiosperms. *Mol Plant*, doi:10.1016/j.molp.2019.10.012 (2019).
11. Fawcett, J. A., Maere, S. & Van de Peer, Y. Plants with double genomes might have had a better chance to survive the Cretaceous-Tertiary extinction event. *P Natl Acad Sci USA* **106**, 5737-5742, doi:10.1073/pnas.0900906106 (2009).
12. Clark, J. W. & Donoghue, P. C. J. Whole-Genome Duplication and Plant Macroevolution. *Trends Plant Sci* **23**, 933-945, doi:10.1016/j.tplants.2018.07.006 (2018).
13. Chase, M. W. *et al.* An update of the Angiosperm Phylogeny Group classification for the orders and families of flowering plants: APG IV. *Botanical Journal of the Linnean Society* **181**, 1-20 (2016).
14. Moore, M. J., Bell, C. D., Soltis, P. S. & Soltis, D. E. Using plastid genome-scale data to resolve enigmatic relationships among basal angiosperms. *Proceedings of the National Academy of Sciences* **104**, 19363-19368 (2007).
15. Moore, M. J., Soltis, P. S., Bell, C. D., Burleigh, J. G. & Soltis, D. E. Phylogenetic analysis of 83 plastid genes further resolves the early diversification of eudicots. *P Natl Acad Sci USA* **107**, 4623-4628, doi:10.1073/pnas.0907801107 (2010).
16. Moore, M. J. *et al.* Phylogenetic Analysis of the Plastid Inverted Repeat for 244 Species: Insights into Deeper-Level Angiosperm Relationships from a Long, Slowly Evolving Sequence Region. *Int J Plant Sci* **172**, 541-558, doi:10.1086/658923 (2011).
17. Yin-Long, Q. *et al.* Angiosperm phylogeny inferred from sequences of four mitochondrial genes. *Journal of Systematics and Evolution* **48**, 391-425 (2010).

18 Endress, P. K. & Doyle, J. A. Reconstructing the ancestral angiosperm flower and its initial specializations. *American Journal of Botany* **96**, 22-66 (2009).

19 Zhang, N., Zeng, L., Shan, H. & Ma, H. Highly conserved low-copy nuclear genes as effective markers for phylogenetic analyses in angiosperms. *New Phytologist* **195**, 923-937 (2012).

20 Barkman, T. J. *et al.* Independent and combined analyses of sequences from all three genomic compartments converge on the root of flowering plant phylogeny. *P Natl Acad Sci USA* **97**, 13166-13171, doi:DOI 10.1073/pnas.220427497 (2000).

21 Qiu, Y. L. *et al.* Phylogenetic analyses of basal angiosperms based on nine plastid, mitochondrial, and nuclear genes. *Int J Plant Sci* **166**, 815-842, doi:Doi 10.1086/431800 (2005).

22 Qiu, Y.-L. *et al.* The earliest angiosperms: evidence from mitochondrial, plastid and nuclear genomes. *Nature* **402**, 404 (1999).

23 Zanis, M. J., Soltis, D. E., Soltis, P. S., Mathews, S. & Donoghue, M. J. The root of the angiosperms revisited. *Proc Natl Acad Sci U S A* **99**, 6848-6853, doi:10.1073/pnas.092136399 (2002).

24 Hilu, K. W. *et al.* Angiosperm phylogeny based on matK sequence information. *American Journal of Botany* **90**, 1758-1776, doi:DOI 10.3732/ajb.90.12.1758 (2003).

25 Qiu, Y. L. *et al.* Reconstructing the basal angiosperm phylogeny: evaluating information content of mitochondrial genes. *Taxon* **55**, 837-856, doi:Doi 10.2307/25065680 (2006).

26 Goremykin, V. V. *et al.* The Evolutionary Root of Flowering Plants. *Syst Biol* **62**, 50-61, doi:10.1093/sysbio/sys070 (2013).
