# Supplementary material for: Prickly waterlily and rigid hornwort genomes shed light on early angiosperm evolution
Source: Nat Plants. 2020 Feb 24;6(3):215–22. doi: 10.1038/s41477-020-0594-6 (PMC8075997; doi:10.1038/s41477-020-0594-6)
Supplement: Supplementary file 2 — Reporting Summary [file 41477_2020_594_MOESM2_ESM.pdf]

nature research

Corresponding author(s): Jianquan Liu

Last updated by author(s): Dec 25, 2019

Reporting Summary

Nature Research wishes to improve the reproducibility of the work that we publish. This form provides structure for consistency and transparency in reporting. For further information on Nature Research policies, see Authors & Referees and the Editorial Policy Checklist.

Statistics

For all statistical analyses, confirm that the following items are present in the figure legend, table legend, main text, or Methods section.

n/a	Confirmed	
☐	☒	The exact sample size (*n*) for each experimental group/condition, given as a discrete number and unit of measurement
☐	☒	A statement on whether measurements were taken from distinct samples or whether the same sample was measured repeatedly
☐	☒	The statistical test(s) used AND whether they are one- or two-sided<br>*Only common tests should be described solely by name; describe more complex techniques in the Methods section.*
☒	☐	A description of all covariates tested
☐	☒	A description of any assumptions or corrections, such as tests of normality and adjustment for multiple comparisons
☒	☐	A full description of the statistical parameters including central tendency (e.g. means) or other basic estimates (e.g. regression coefficient) AND variation (e.g. standard deviation) or associated estimates of uncertainty (e.g. confidence intervals)
☐	☒	For null hypothesis testing, the test statistic (e.g. *F*, *t*, *r*) with confidence intervals, effect sizes, degrees of freedom and *P* value noted<br>*Give P values as exact values whenever suitable.*
☐	☒	For Bayesian analysis, information on the choice of priors and Markov chain Monte Carlo settings
☒	☐	For hierarchical and complex designs, identification of the appropriate level for tests and full reporting of outcomes
☐	☒	Estimates of effect sizes (e.g. Cohen's *d*, Pearson's *r*), indicating how they were calculated

Our web collection on statistics for biologists contains articles on many of the points above.

Software and code

Policy information about availability of computer code

Data collection	No software was used to collect the data.
Data analysis	A lot of software were used for data analysis in this paper.<br>Genome size estimation: SOAPec v2.0.1.<br>Genome assembly: Canu v1.7, Pilon v1.22, HiC-Pro v2.10.0 and LACHESIS (https://github.com/shendurelab/LACHESIS).<br>Genome assessment: bwa v0.7.12-r1039, Trinity v2.8.4 and BLAT v35.<br>Genome annotation: Tandem Repeats Finder v4.04, RepeatMasker v4.0.7, RepeatModeler v1.0.11, TBLASTN v2.3.0, BLASTP v2.3.0, InterProScan, tRNAscan-SE v1.3.1, BLASTN v2.3.0, PASA v2.3.3, AUGUSTUS v3.2.3 and EvidenceModeler v1.1.1.<br>Polyploidization analysis: MCScan v0.8, PAML v4.9h and MATLAB.<br>Phylogenetic analyses: SonicParanoid, MAFFT v7.402, PAL2NAL v14, IQ-TREE v1.6.9, ASTRAL v5.6.1, OrthoMCL v2.0.9, STAG v1.0.0, MMseqs2 v7-4e23d and MCL v14-137.<br>Divergence time estimation: MCMCTree in the PAML package v4.9h.<br>Inferring hybridization and ILS: PhyloNetworks v0.0.0 and Phybase v1.5.<br>Demographic inference: bwa v0.7.17-r1188, SAMtools v1.6 and PSMC v0.6.4-r49.

For manuscripts utilizing custom algorithms or software that are central to the research but not yet described in published literature, software must be made available to editors/reviewers. We strongly encourage code deposition in a community repository (e.g. GitHub). See the Nature Research guidelines for submitting code & software for further information.

Data

Policy information about availability of data

All manuscripts must include a data availability statement. This statement should provide the following information, where applicable:

- Accession codes, unique identifiers, or web links for publicly available datasets
- A list of figures that have associated raw data
- A description of any restrictions on data availability

All the raw sequence reads used in this study have been deposited in NCBI under the BioProject accession numbers PRJNA552436 (E. ferox) and PRJNA552433 (C. demersum). The assemblies and annotations are available from the CoGe comparative genomics platform: https://genomevolution.org/CoGe/GenomeInfo.pl?gid=56574 (E. ferox chromosome assembly), https://genomevolution.org/CoGe/GenomeInfo.pl?gid=56571 (E. ferox contig assembly), https://genomevolution.org/CoGe/GenomeInfo.pl?gid=56572 (C. demersum chromosome assembly) and https://genomevolution.org/CoGe/GenomeInfo.pl?gid=56569 (C. demersum contig assembly). The custom scripts have deposited in GitHub (https://github.com/yongzhiyang2012/Euryale_ferox_and_Ceratophyllum_demersum_genome_analysis).

Field-specific reporting

Please select the one below that is the best fit for your research. If you are not sure, read the appropriate sections before making your selection.

☒ Life sciences ☐ Behavioural & social sciences ☐ Ecological, evolutionary & environmental sciences

For a reference copy of the document with all sections, see nature.com/documents/nr-reporting-summary-flat.pdf

Life sciences study design

All studies must disclose on these points even when the disclosure is negative.

Sample size	One Prickly waterlily and one rigid hornwort individuals were selected containing the sufficiently fresh samples to the genome sequencing. As the very low heterozygosity detected within Prickly waterlily, another individual was needed and sequenced for the PSMC analysis.
Data exclusions	For the long reads, we have removed the reads with a mean quality score < 7. For the short Illumina reads, the following criteria were performed to filter the low quality reads: (i) containing more than 5% unidentified nucleotides, (ii) more than 65% of bases with a Phred quality score < 7, and (iii) more than 10 bp adapter sequences (allowing 2 bp mismatches)
Replication	No replication in this manuscript.
Randomization	No randomization in this manuscript as the genome assembly, annotation and comparison no needed randomization. For the phylogeny analysis we used all the identified single copy genes or low copy genes, not underling a randomization.
Blinding	The two genome were sequenced and assembled with no blinding as data were not allocated into groups.

Reporting for specific materials, systems and methods

We require information from authors about some types of materials, experimental systems and methods used in many studies. Here, indicate whether each material, system or method listed is relevant to your study. If you are not sure if a list item applies to your research, read the appropriate section before selecting a response.

Materials & experimental systems

n/a	Involved in the study
☒	☐ Antibodies
☒	☐ Eukaryotic cell lines
☒	☐ Palaeontology
☒	☐ Animals and other organisms
☒	☐ Human research participants
☒	☐ Clinical data

Methods

n/a	Involved in the study
☒	☐ ChIP-seq
☒	☐ Flow cytometry
☒	☐ MRI-based neuroimaging
